# Supplementary material for: Upregulated ECM genes and increased synaptic activity in Parkinson’s human DA neurons with PINK1/ PRKN mutations
Source: NPJ Parkinsons Dis. 2024 May 18;10:103. doi: 10.1038/s41531-024-00715-0 (PMC11102563; doi:10.1038/s41531-024-00715-0)
Supplement: Supplementary file 1 — Supplementary file [file 41531_2024_715_MOESM1_ESM.pdf]

Supplementary Figure Legends

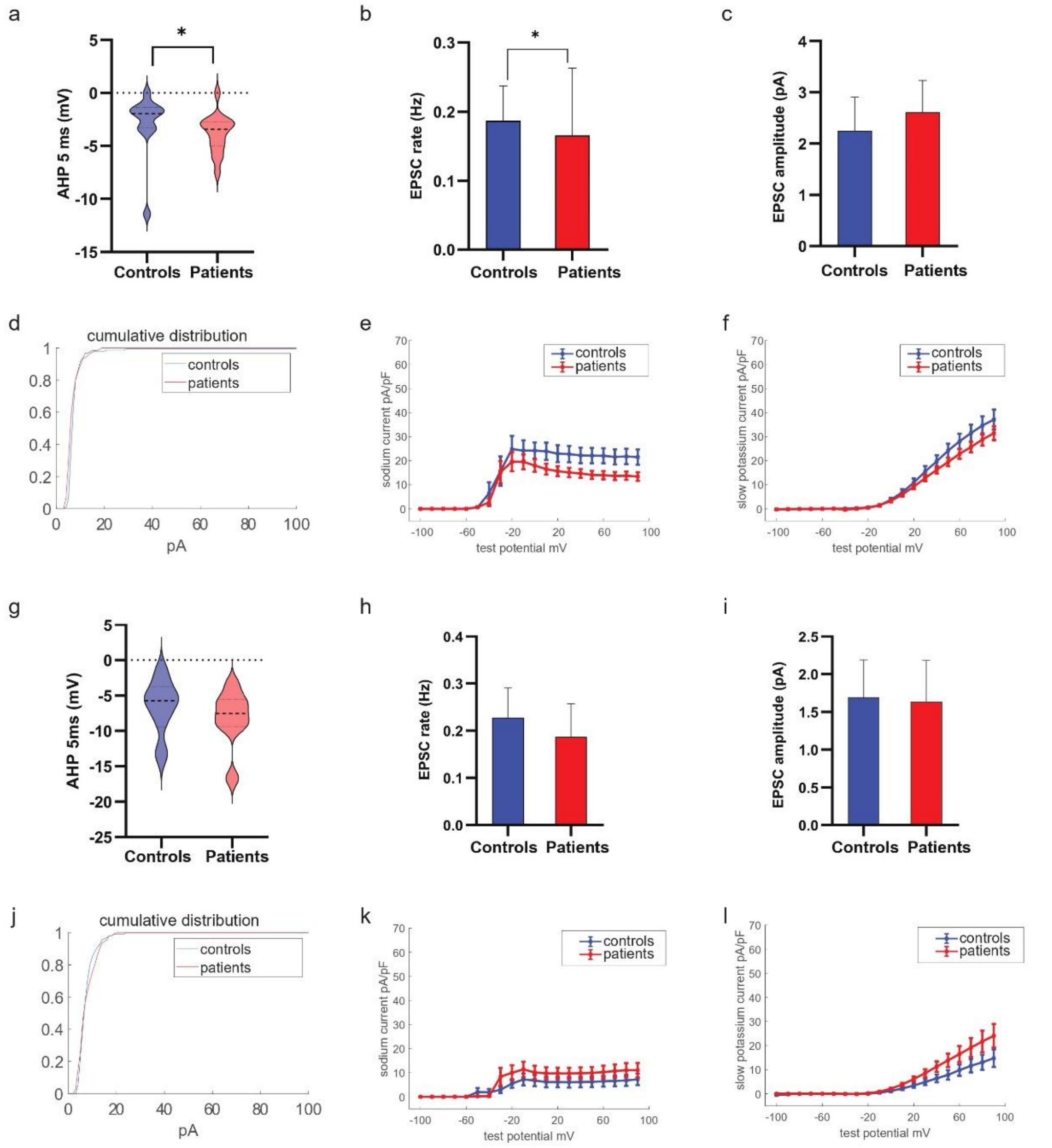

Supplementary figure 1

Hippocampal *PINK1* and *PRKN* mutant neuron's synaptic activity was not different compared to controls during early and post-maturation periods. (a-f). During the 2-4 weeks post differentiation period (first time period) of hippocampal neurons, (a) the 5 ms AHP was significantly reduced in *PINK1* and *PRKN* mutant neurons. (b) The EPSC rate was decreased in *PINK1* and *PRKN* mutant neurons compared to healthy controls. (c) The EPSC amplitude was not significantly different between *PINK1* and *PRKN* mutant neurons and control neurons. (d) The cumulative distribution of EPSC amplitude showed that EPSC amplitude distribution was similar for *PINK1* and *PRKN* mutant and control neurons. Initially, (e) the sodium and (f) the slow potassium currents were significantly decreased in *PINK1* and *PRKN* mutant neurons compared to control neurons. g-l. During the 8-11 weeks post differentiation period (the third time point) in hippocampal neurons (g), the 5 ms AHP was not significantly different in *PINK1* and

PRKN mutant neurons. (h) The EPSC rate was not significantly different between *PINK1* and *PRKN* mutant and control neurons. (i) Also, the EPSC amplitude was not significantly different between *PINK1* and *PRKN* mutant and control neurons. (j) The cumulative distribution of EPSC amplitudes showed that EPSC amplitudes were similar for *PINK1* and *PRKN* mutant and control neurons. In the later phase (third-time point), (k) the sodium and (l) the slow potassium currents became significantly larger in *PINK1* and *PRKN* mutant neurons compared to control neurons. Unless otherwise noted, the error bars in this and the following figures indicate the standard error. Asterisks in this and the subsequent figures denote statistical significance as indicated by the following codes: * $p<0.05$.

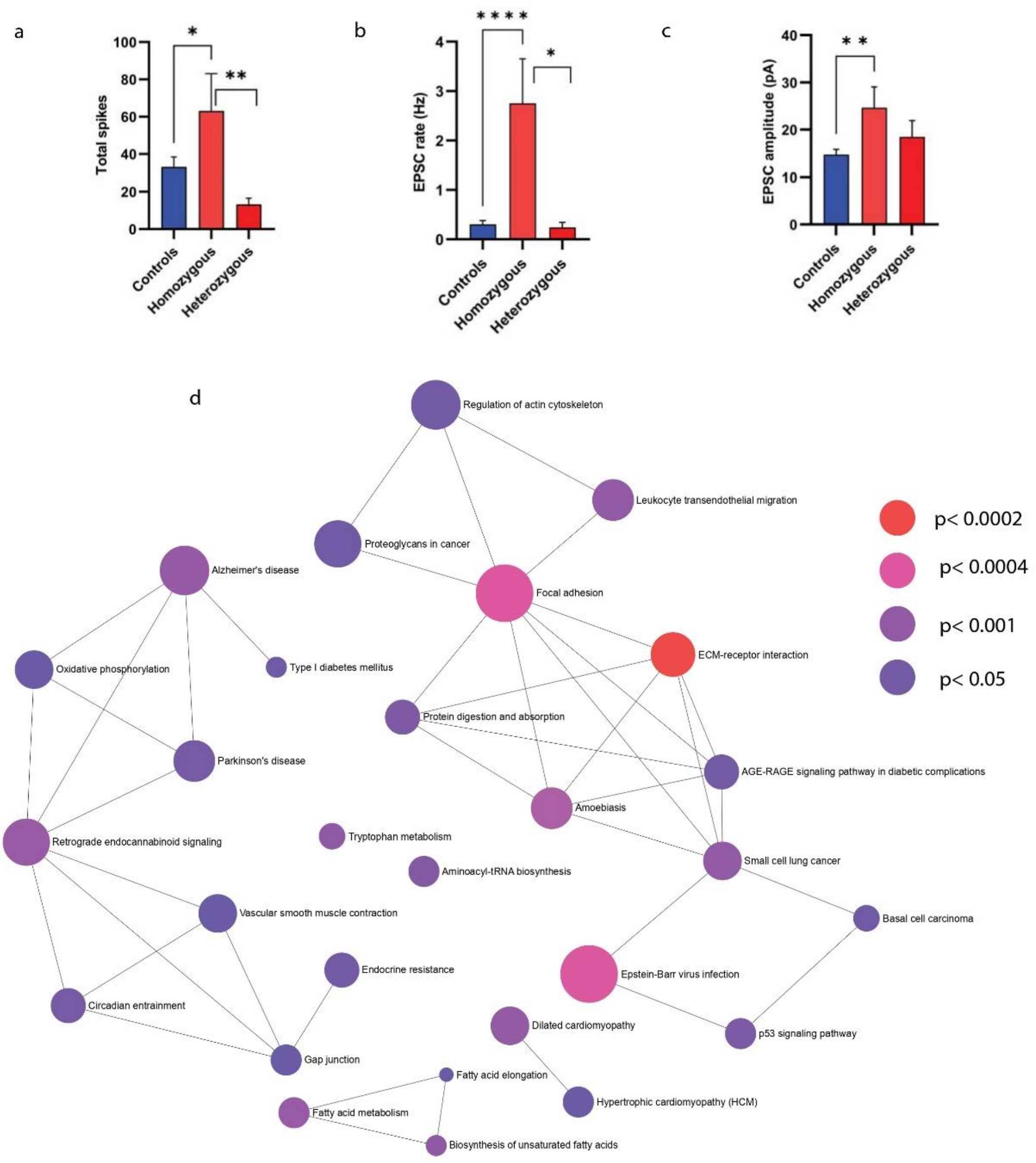

Supplementary figure 2

DA neurons derived from patients with homozygous *PINK1* and *PRKN* mutations showed a strong increase in synaptic activity. (a) The total evoked number of spikes in DA neurons with homozygous *PINK1* and *PRKN* mutations were significantly increased compared to control neurons and neurons with heterozygous *PINK1* and *PRKN* mutations. (b) The EPSC rate in DA neurons with the homozygous *PINK1* mutation was significantly increased compared to control neurons and neurons from the heterozygous *PINK1* mutant patient with the heterozygous *PINK1* mutation. (c) Also, the EPSC amplitude was significantly increased in the homozygous *PINK1* mutant neurons compared to the control and the heterozygous *PINK1* mutant lines. (d) Signaling network analysis with the top enriched KEGG pathways for the homozygous *PINK1* and *PRKN* patient's-derived neurons compared to the controls shows that the top dysregulated pathways for this double mutation are "Epstein Barr virus infection," "ECM receptor interaction," and "Focal adhesion."

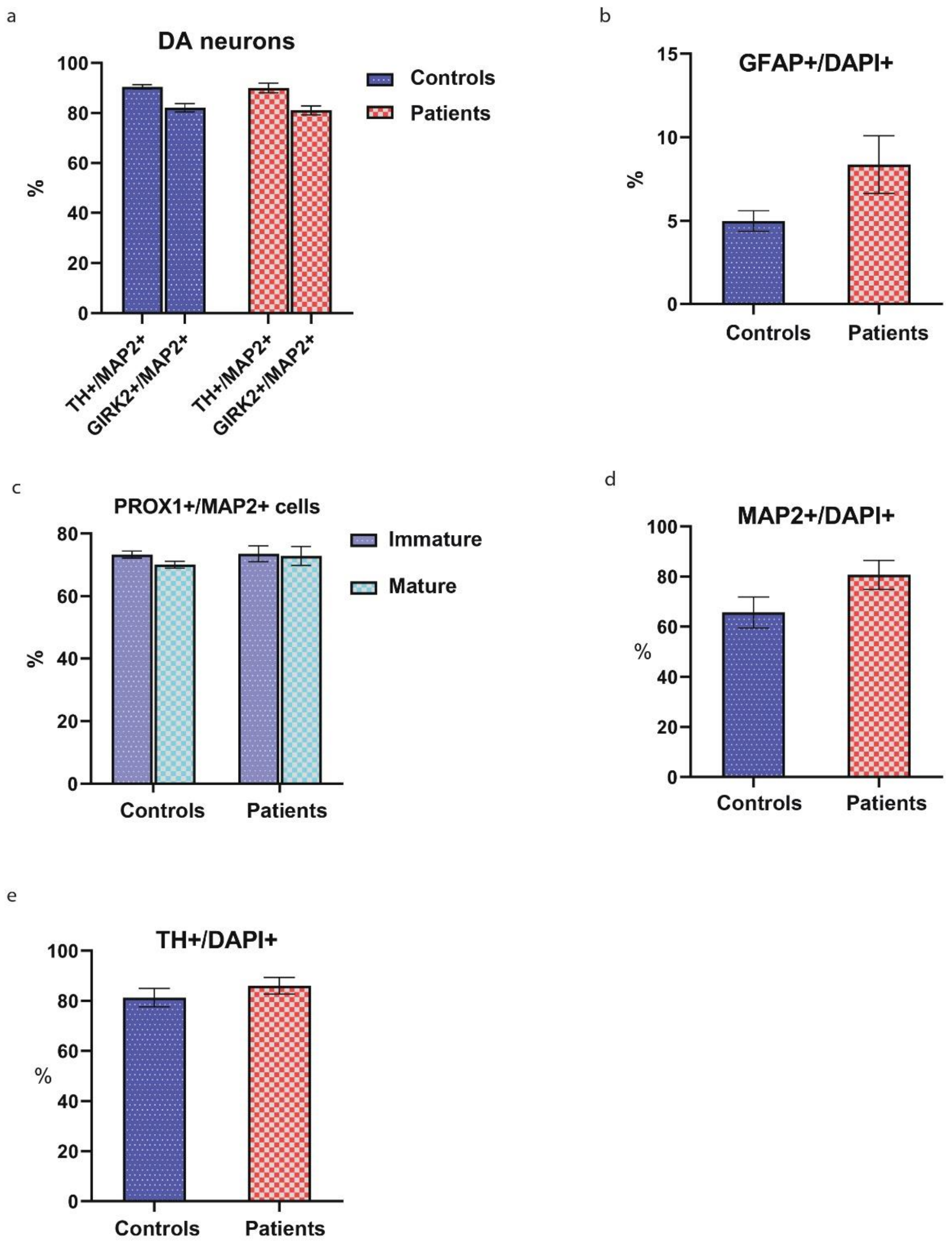

Supplementary figure 3

Quantification of cell types in DA and hippocampal cultures. (a) TH+/MAP2+ neurons were quantified in the cultures derived from controls and *PINK1* and *PRKN* mutants to quantify the percentage of DA neurons within the culture. In the control cultures, these neurons represented 90.5±2 (std) % of the population, while in *PINK1* and *PRKN* mutant cultures, the percentage was slightly less, at 90±4 (std) %. Likewise, for GIRK2+/MAP2+ neurons, they constituted 82± 4 (std) % in control cultures and 81± 4 (std) % in *PINK1* and *PRKN* mutant cultures. (b) In addition to neurons, GFAP+ astrocytes were also quantified, showing a slight increase in the *PINK1* and *PRKN* mutant cultures (not significant). These cells made up 5 ± 1

(std) % in control cultures and 7 ± 4.5 % (std) in *PINK1* and *PRKN* mutant cultures. (c) Hippocampal neuronal cultures consisted of PROX1+ neurons. We quantified the percentage of PROX1+/MAP2+ neurons in the culture. During the immature phase (first-time point), these neurons made up 73± 2.5% (std) in control cultures and 74 ± 6% (std) in *PINK1* and *PRKN* mutant cultures. In the mature phase (second-time point), these neurons constituted 70 ± 2. (std)% of control cultures and 73± 7% (std) of *PINK1* and *PRKN* mutant cultures. The error bars in this figure indicate the standard error. (d) The percentage of MAP2+/DAPI+ and (e) TH+/DAPI+ neurons were quantified in DA neuron cultures derived from controls and *PINK1* and *PRKN* mutants. In control neurons, 66±12% DAPI positive cells expressed MAP2, and 81±7% DAPI positive cells expressed TH. In *PINK1* and *PRKN* mutant DA cultures, 81±12% DAPI positive cells expressed MAP2, and 86±7% DAPI positive cells expressed TH.

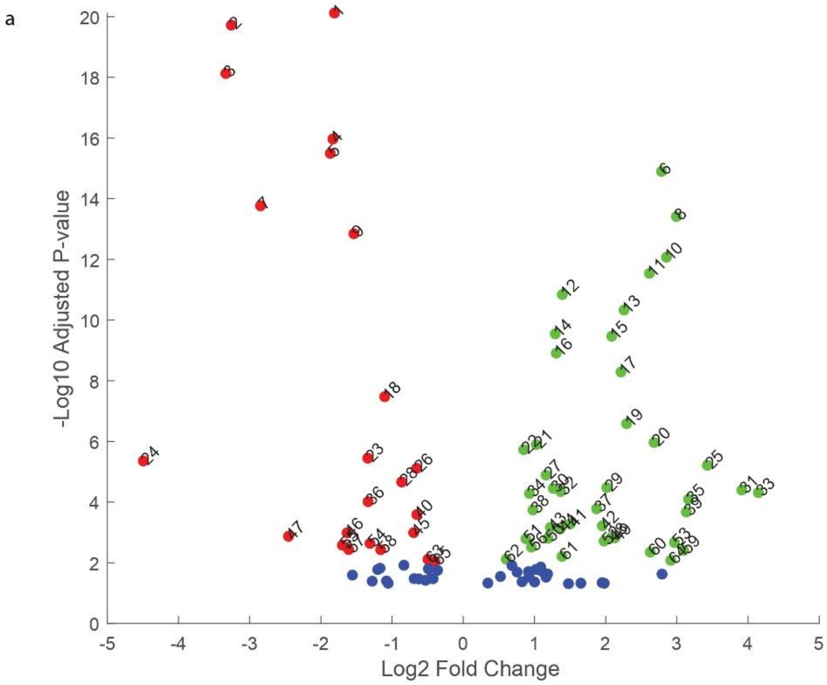

Supplementary figure 4

(a) The entire set of significantly dysregulated Mitochondria genes (matched with the MitoCarta Human 3.0 gene set) in *PINK1* and *PRKN* mutant DA neurons compared to healthy controls is plotted as a volcano plot. We identified 97 mitochondrial genes within our set of DEGs. Among these, 21 genes were downregulated, and 42 were upregulated with a threshold of 1.1 log fold change (FDR<0.05).

Labeled genes are as follows:

'1: C12orf65' '2: HADHA' '3: IDH3A' '4: SLC25A26' '5: TMEM186' '6: MTHFD2' '7: POLG' '8: PTRH1' '9: NDUFB9' '10: MTERF2' '11: MTERF4' '12: SPRYD4' '13: MT-ND1' '14: BID' '15: MT-ND2' '16: POLDIP2' '17: ACAD8' '18: PC' '19: UQCC2' '20: DLD' '21: MALSU1' '22: COX11' '23: DNAJC11' '24: METAP1D' '25: D2HGDH' '26: PCCB' '27: AFG3L2' '28: LYRM2' '29: DIABLO' '30: KMO' '31: SPTLC2' '32: CBR4' '33: CYP11A1' '34: TTC19' '35: GFM1' '36: ACADVL' '37: COA1' '38: NDUFS7' '39: NUDT8' '40: PTRH2' '41: GFER' '42: COA3' '43:

MTFR1' '44: MAVS' '45: NAXE' '46: DHRS7B' '47: PDK3' '48: AMT' '49: NDUFV1' '50: MT-CYB' '51: MT-ND3' '52: LACTB' '53: SLC8B1' '54: GRPEL1' '55: PRORP' '56: HTRA2' '57: RECQL4' '58: NDUFC1' '59: MGST1' '60: RDH14' '61: SLC25A3' '62: GLRX2' '63: NIT1' '64: GATB' '65: GLYCTK'

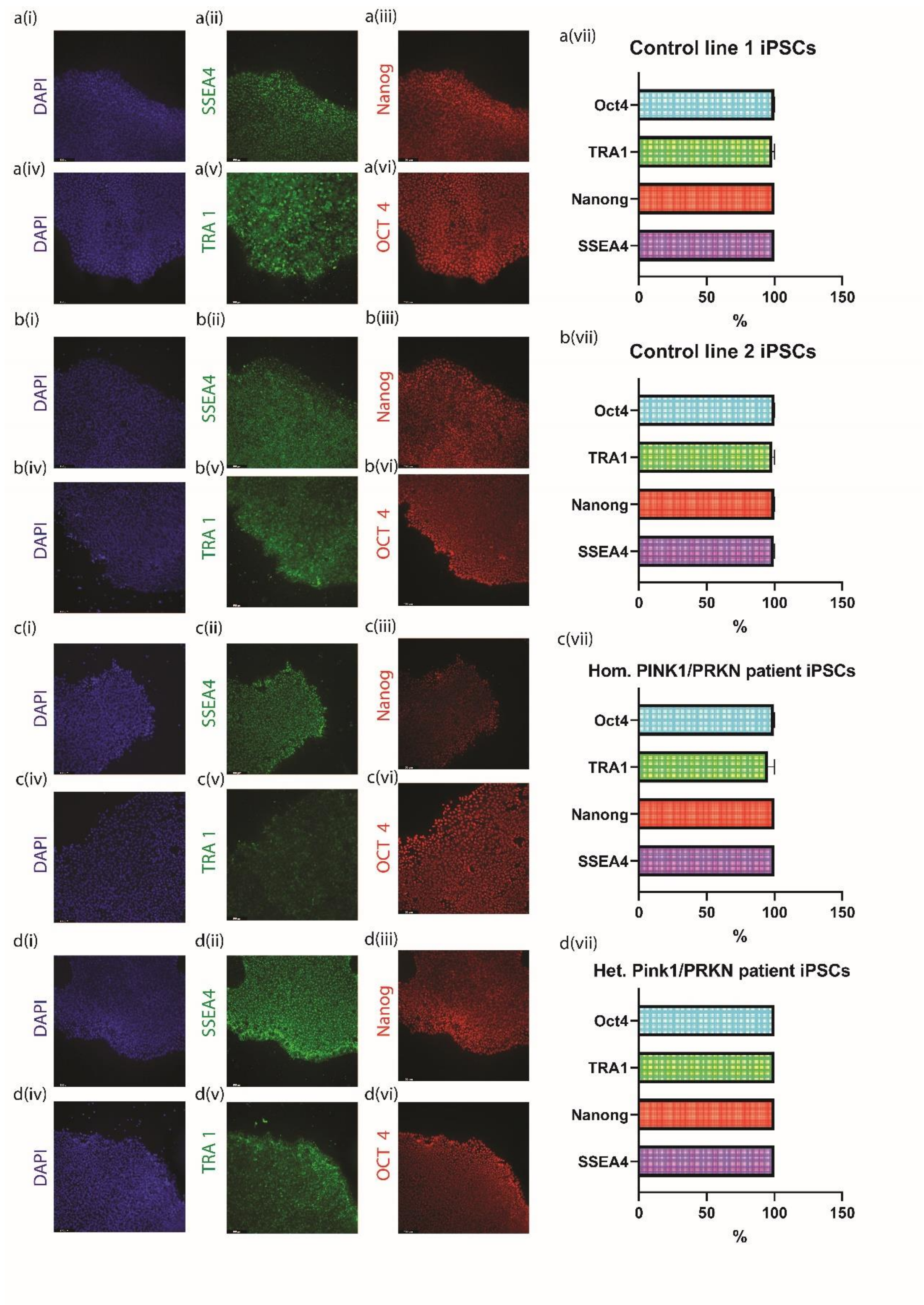

Supplementary figure 5

Assessment and quantification of Pluripotency Markers in hiPSC lines in control and mutant iPSC lines. Control line 1 is shown with DAPI nuclear staining (a(i),a(iv)), surface marker SSEA4 (a(ii)), and pluripotency transcription factors Nanog (a(iii)), TRA-1(a(v)), and Oct4 (a(vi)). Control line 2 is represented by similar staining patterns (b(i)-b(vi)). Homozygous *PINK1* and *PRKN* mutant iPSCs are shown in (c(i)-c(vi)), and Heterozygous *PINK1* and *PRKN* mutant iPSCs are depicted in (d(i)-d(vi)), demonstrating similar pluripotency marker expression. The quantitative analysis of pluripotency markers Oct4, TRA1, Nanog, and SSEA4 is presented for Control line 1 (a(vii)), Control line 2 (b(vii)), Homozygous *PINK1* and *PRKN* mutant iPSCs (c(vii)), and Heterozygous *PINK1* and *PRKN* mutant iPSCs (d(vii)). The histograms indicate a high percentage of positive cells for each marker, indicating a pluripotent status. The data is presented as mean ± SEM.

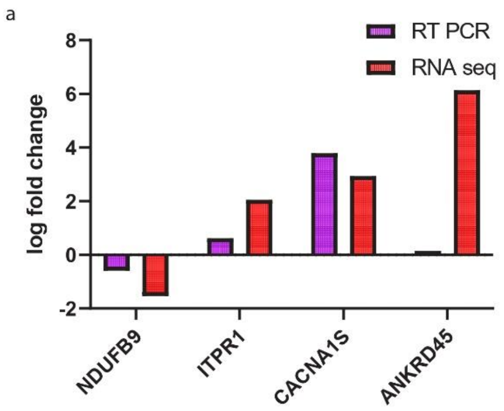

Supplementary figure 6

Validation of gene expression dysregulation in *PINK1* and *PRKN* mutant DA neurons compared to healthy controls using RT PCR. The graphs display the log fold change in 4 chosen genes (*NDUFB9* - mitochondrial gene, *ITPR1*- calcium signaling related gene, *CACNA1S* - calcium signaling related gene, and *ANKRD45* - a gene related to cell proliferation) as determined by RT-qPCR and RNA sequencing (RNA-seq). The purple bars represent RT-qPCR results, while the red bars denote RNA-seq results. The direction of change in expression levels was similar between RT-qPCR experiments and RNA-seq, although the fold change varied between the two methods.

Supplemmentary table 1 (a)					
**Pathway**	Total	Expected	Hits	P.Value	FDR
**Hypertrophic cardiomyopathy (HCM)**	85	2.89	11	0.00013	0.0414
**Focal adhesion**	199	6.76	17	0.000417	0.0438
**ECM-receptor interaction**	82	2.79	10	0.000429	0.0438
**AGE-RAGE signaling pathway in diabetic complications**	100	3.4	11	0.000551	0.0438
**Dilated cardiomyopathy**	91	3.09	10	0.000985	0.0627
**Leukocyte transendothelial migration**	112	3.81	11	0.00142	0.0755
**p53 signaling pathway**	72	2.45	8	0.00292	0.132
**Thyroid cancer**	37	1.26	5	0.00782	0.311
**Arrhythmogenic right ventricular cardiomyopathy (ARVC)**	72	2.45	7	0.0108	0.356
**Protein digestion and absorption**	90	3.06	8	0.0112	0.356
**Amoebiasis**	96	3.26	8	0.0161	0.434
**Hepatitis C**	155	5.27	11	0.0164	0.434
**TNF signaling pathway**	110	3.74	8	0.0333	0.765
**TGF-beta signaling pathway**	92	3.13	7	0.0366	0.765
**Platinum drug resistance**	73	2.48	6	0.0372	0.765
**Small cell lung cancer**	93	3.16	7	0.0385	0.765
**Epstein-Barr virus infection**	201	6.83	12	0.0411	0.768
**Cellular senescence**	160	5.44	10	0.0458	0.786
**Cardiac muscle contraction**	78	2.65	6	0.0489	0.786
**Bladder cancer**	41	1.39	4	0.0494	0.786

Supplementary Table 1. KEGG pathway enrichment analysis of differentially expressed genes (DEGs) in *PINK1* and *PRKN* mutant DA neurons versus control neurons. The table lists significantly upregulated pathways, the number of genes in each pathway, the expected number of DEGs in the pathway, the actual number of DEGs observed in the pathway, p-values from the performed enrichment test, and Benjamini-Hochberg adjusted FDR p-values (q-values). Pathways are ordered by statistical significance.

**Supplementary table 1(b)**					
**Pathway**	Total	Expected	Hits	P.Value	FDR
**ECM-receptor interaction**	82	2.47	10	0.000161	0.0374
**Focal adhesion**	199	5.99	16	0.000315	0.0374
**Epstein-Barr virus infection**	201	6.05	16	0.000353	0.0374
**Amoebiasis**	96	2.89	9	0.00232	0.184
**Fatty acid metabolism**	53	1.6	6	0.00492	0.215
**Retrograde endocannabinoid signaling**	148	4.46	11	0.00497	0.215
**Alzheimer's disease**	171	5.15	12	0.0054	0.215
**Dilated cardiomyopathy**	91	2.74	8	0.00594	0.215
**Leukocyte transendothelial migration**	112	3.37	9	0.0065	0.215
**Small cell lung cancer**	93	2.8	8	0.00676	0.215
**Tryptophan metabolism**	42	1.26	5	0.00814	0.216
**Biosynthesis of unsaturated fatty acids**	27	0.813	4	0.00816	0.216
**Aminoacyl-tRNA biosynthesis**	66	1.99	6	0.0141	0.345
**Protein digestion and absorption**	90	2.71	7	0.0185	0.42
**p53 signaling pathway**	72	2.17	6	0.0209	0.443
**Circadian entrainment**	97	2.92	7	0.0267	0.469
**Parkinson's disease**	142	4.28	9	0.0273	0.469
**Regulation of actin cytoskeleton**	214	6.44	12	0.028	0.469
**Endocrine resistance**	98	2.95	7	0.028	0.469
**AGE-RAGE signaling pathway in diabetic complications**	100	3.01	7	0.0308	0.49
**Type I diabetes mellitus**	43	1.29	4	0.0395	0.556
**Proteoglycans in cancer**	201	6.05	11	0.0402	0.556
**Basal cell carcinoma**	63	1.9	5	0.0406	0.556
**Hypertrophic cardiomyopathy (HCM)**	85	2.56	6	0.0426	0.556
**Vascular smooth muscle contraction**	132	3.98	8	0.0455	0.556
**Fatty acid elongation**	27	0.813	3	0.0463	0.556
**Oxidative phosphorylation**	133	4.01	8	0.0472	0.556
**Gap junction**	88	2.65	6	0.049	0.557

Supplementary Table 1(b). KEGG pathway enrichment analysis of differentially expressed genes (DEGs) in homozygous *PINK1* mutant DA neurons versus control neurons. The table lists significantly dysregulated pathways, the number of genes in each pathway, the expected number of DEGs in the pathway, the actual number of DEGs observed in the pathway, p-values from the performed enrichment test, and Benjamini-Hochberg adjusted FDR p-values (q-values). Pathways are ordered by statistical significance.

Supplementary table 1(c)					
**Pathway**	Total	Expected	Hits	P.Value	FDR
**Extracellular matrix structural constituent**	80	2.88	12	2.69E-05	0.0104
**Actin binding**	373	13.4	29	8.46E-05	0.0164
**Cytoskeletal protein binding**	738	26.6	44	0.000691	0.0893
**Heparin binding**	130	4.68	12	0.00254	0.246
**Glycosaminoglycan binding**	178	6.4	14	0.00507	0.393
**Structural constituent of muscle**	49	1.76	6	0.00793	0.513
**Structural molecule activity**	666	24	35	0.016	0.886
**Oxidoreductase activity, acting on NAD(P)H**	117	4.21	9	0.0253	1
**Single_stranded DNA binding**	65	2.34	6	0.0291	1
**ATP_dependent RNA helicase activity**	21	0.756	3	0.038	1
**Cation binding**	4160	150	168	0.0401	1
**Actin filament binding**	71	2.55	6	0.0422	1
**RNA_dependent ATPase activity**	22	0.792	3	0.0429	1
**GTPase binding**	150	5.4	10	0.0445	1
**Calmodulin binding**	171	6.15	11	0.045	1

Supplementary Table 1(c). Gene ontology (GO) molecular function enrichment analysis of DEGs with |log2 fold change| > 1.1 in *PINK1* and *PRKN* mutant DA neurons versus control neurons. The table displays significantly upregulated terms with stats and enrichment analysis results displayed as in Supplementary Table 1(c).

Supplementary figure 1(d)					
**Pathway**	Total	Expected	Hits	P.Value	FDR
**Extracellular matrix structural constituent**	80	1.71	9	5.14E-05	0.0199
**Heparin binding**	130	2.78	11	0.00011	0.0208
**Actin binding**	373	7.98	20	0.000161	0.0208
**Glycosaminoglycan binding**	178	3.81	12	0.000457	0.0443
**Structural molecule activity**	666	14.3	27	0.00112	0.0871
**Actin filament binding**	71	1.52	6	0.00409	0.264
**Chromatin binding**	338	7.24	15	0.00632	0.35
**Cytoskeletal protein binding**	738	15.8	26	0.0088	0.427
**Integrin binding**	86	1.84	6	0.0103	0.431
**Ubiquitin binding**	63	1.35	5	0.0111	0.431
**Collagen binding**	47	1.01	4	0.0178	0.581
**Growth factor binding**	125	2.68	7	0.018	0.581
**Coenzyme binding**	188	4.02	9	0.0201	0.598
**Small conjugating protein binding**	75	1.61	5	0.0222	0.616
**Transcription cofactor activity**	552	11.8	19	0.0287	0.743
**Calmodulin binding**	171	3.66	8	0.031	0.751
**Protein binding transcription factor activity**	600	12.8	20	0.0338	0.771
**Oxidoreductase activity, acting on NAD(P)H**	117	2.5	6	0.0399	0.859

Supplementary Table 1(d). GO molecular function enrichment analysis of DEGs with |log2 fold change| > 1.1 in homozygous *PINK1* mutant DA neurons versus control neurons. The table displays significantly upregulated terms with stats and enrichment analysis results displayed as in Supplementary Table 1(d).

**Supplementary table 1(e)**					
**Pathway**	Total	Expected	Hits	P.Value	FDR
**Endoplasmic reticulum lumen**	175	6.09	19	1.15E-05	0.00258
**Extracellular matrix part**	204	7.1	20	3.06E-05	0.00345
**Proteinaceous extracellular matrix**	398	13.8	30	6.37E-05	0.00477
**Basement membrane**	100	3.48	12	0.000181	0.0102
**Extracellular region part**	1320	45.9	68	0.000687	0.0309
**Extracellular matrix**	570	19.8	34	0.00163	0.053
**Secretory granule**	276	9.6	20	0.00165	0.053
**Tight junction**	105	3.65	10	0.00359	0.0946
**Actin cytoskeleton**	430	15	26	0.00462	0.0946
**Integrin complex**	32	1.11	5	0.00463	0.0946
**Cell_cell junction**	346	12	22	0.00496	0.0946
**Collagen**	93	3.24	9	0.00504	0.0946
**Apical junction complex**	123	4.28	10	0.0107	0.186
**Sarcomere**	163	5.67	12	0.0117	0.188
**Contractile fiber part**	187	6.51	13	0.0139	0.209
**Neuron projection**	685	23.8	35	0.0151	0.213
**Cortical cytoskeleton**	60	2.09	6	0.0177	0.22
**Contractile fiber**	214	7.45	14	0.0178	0.22
**Cell cortex**	195	6.78	13	0.0191	0.22
**Ruffle**	135	4.7	10	0.0196	0.22
**Myofibril**	197	6.85	13	0.0206	0.22
**Extracellular space**	901	31.3	42	0.0329	0.322
**Receptor complex**	189	6.58	12	0.0329	0.322
**Membrane_bounded vesicle**	1100	38.2	49	0.0439	0.385
**Apicolateral plasma membrane**	10	0.348	2	0.0452	0.385
**Cell junction**	847	29.5	39	0.0454	0.385
**Myosin complex**	75	2.61	6	0.0462	0.385
**U12_type spliceosomal complex**	24	0.835	3	0.0493	0.396

Supplementary Table 1(e). GO cellular components enrichment analysis of DEGs with |log2 fold change| > 1.1 in *PINK1* and *PRKN* mutant DA neurons versus control neurons. The table displays significantly upregulated terms with stats and enrichment analysis results displayed as in Supplementary Table 1(e).

**Supplememntary table 1(f)**					
**Pathway**	Total	Expected	Hits	P.Value	FDR
**Extracellular matrix part**	204	4.3	15	2.87E-05	0.00646
**Proteinaceous extracellular matrix**	398	8.38	21	0.000114	0.0128
**Basement membrane**	100	2.11	9	0.000259	0.0192
**Endoplasmic reticulum lumen**	175	3.68	12	0.000342	0.0192
**Extracellular matrix**	570	12	25	0.00046	0.0207
**Collagen**	93	1.96	8	0.000767	0.0288
**Extracellular region part**	1320	27.8	43	0.00279	0.0896
**Histone deacetylase complex**	53	1.12	5	0.00504	0.142
**Cortical cytoskeleton**	60	1.26	5	0.00852	0.213
**U12_type spliceosomal complex**	24	0.505	3	0.0135	0.293
**Secretory granule**	276	5.81	12	0.0143	0.293
**Mitochondrial membrane part**	217	4.57	10	0.0169	0.317
**Actin cytoskeleton**	430	9.05	16	0.0204	0.317
**Myosin complex**	75	1.58	5	0.0209	0.317
**Sarcomere**	163	3.43	8	0.0223	0.317
**Cell cortex**	195	4.11	9	0.0226	0.317
**Myofibril**	197	4.15	9	0.024	0.317
**Integrin complex**	32	0.674	3	0.0292	0.366
**Contractile fiber**	214	4.51	9	0.0378	0.447
**Cortical actin cytoskeleton**	37	0.779	3	0.0425	0.457
**Ribosomal subunit**	154	3.24	7	0.0446	0.457
**Contractile fiber part**	187	3.94	8	0.0447	0.457
**Receptor complex**	189	3.98	8	0.0471	0.46

Supplementary Table 1(f). GO cellular components enrichment analysis of DEGs with |log2 fold change| > 1.1 in homozygous *PINK1* mutant DA neurons versus control neurons. The table displays significantly upregulated terms with stats and enrichment analysis results displayed as in Supplementary Table 1(f).

Supplementary table 1(g)					
Pathway	Total	Expected	Hits	P.Value	FDR
Central nervous system development	784	12.6	34	1.21E-07	9.89E-05
Regulation of neurogenesis	444	7.15	22	2.98E-06	0.00105
Brain development	559	9	25	3.84E-06	0.00105
Gliogenesis	176	2.83	12	2.80E-05	0.00574
Cell fate commitment	254	4.09	14	6.45E-05	0.0106
Neurogenesis	1390	22.4	38	0.000812	0.11
Generation of neurons	1300	21	36	0.000938	0.11
Epidermal growth factor receptor signaling pathway	167	2.69	9	0.00154	0.158
Nervous system development	2190	35.3	52	0.00217	0.197
G2/M transition of mitotic cell cycle	150	2.41	8	0.00297	0.243
Neuron differentiation	1190	19.1	31	0.00493	0.366
Myoblast differentiation	44	0.708	4	0.00536	0.366
Cell migration	1050	17	28	0.00604	0.381
Regulation of axonogenesis	104	1.67	6	0.00668	0.391
Regulation of Rho protein signal transduction	177	2.85	8	0.00796	0.435
Microtubule_based movement	179	2.88	8	0.00849	0.435
Organ morphogenesis	966	15.6	25	0.0127	0.613
Enzyme linked receptor protein signaling pathway	1180	18.9	29	0.0138	0.627
Transmembrane receptor protein tyrosine kinase signaling pathway	782	12.6	21	0.015	0.646
Regulation of cell morphogenesis	325	5.23	11	0.0164	0.671
Regulation of anatomical structure morphogenesis	702	11.3	19	0.0188	0.695
Regulation of Rho GTPase activity	101	1.63	5	0.0236	0.695
Interphase of mitotic cell cycle	435	7	13	0.024	0.695
Regulation of Ras protein signal transduction	302	4.86	10	0.0246	0.695
Tube morphogenesis	347	5.59	11	0.0251	0.695
Gland development	303	4.88	10	0.0251	0.695
Sensory organ development	485	7.81	14	0.0255	0.695
Interphase	443	7.13	13	0.0274	0.695
Fatty acid metabolic process	397	6.39	12	0.0274	0.695
Negative regulation of cell differentiation	540	8.69	15	0.0285	0.695
Positive regulation of cell proliferation	786	12.7	20	0.0289	0.695
Cell development	1840	29.7	40	0.0294	0.695
Phosphatidylinositol_mediated signaling	148	2.38	6	0.0326	0.695
Organelle localization	189	3.04	7	0.0334	0.695
Regulation of cell migration	456	7.34	13	0.0334	0.695
Negative regulation of transcription from RNA polymerase II promoter	552	8.89	15	0.0337	0.695
Response to hormone stimulus	751	12.1	19	0.0344	0.695
Tube development	506	8.15	14	0.0347	0.695
Anatomical structure formation involved in morphogenesis	2090	33.6	44	0.0352	0.695
Fatty acid biosynthetic process	151	2.43	6	0.0354	0.695

**Adenylate cyclase_inhibiting G_protein coupled receptor signaling pathway**	45	0.724	3	0.0356	0.695
**Base_excision repair**	45	0.724	3	0.0356	0.695
**Skeletal muscle tissue development**	197	3.17	7	0.0404	0.723
**Tissue morphogenesis**	566	9.11	15	0.0405	0.723
**Regulation of cell differentiation**	1290	20.7	29	0.0408	0.723
**Regulation of neurotransmitter levels**	157	2.53	6	0.0416	0.723
**Peripheral nervous system development**	81	1.3	4	0.0416	0.723
**Positive regulation of cell differentiation**	571	9.19	15	0.0432	0.723
**Regulation of pH**	49	0.789	3	0.0441	0.723
**Positive regulation of epithelial cell proliferation**	120	1.93	5	0.0446	0.723
**Developmental growth**	290	4.67	9	0.0454	0.723
**Regulation of lipid metabolic process**	246	3.96	8	0.0459	0.723
**Cell_cell signaling**	1310	21	29	0.0474	0.734
**Spermatid differentiation**	86	1.38	4	0.05	0.758

Supplementary Table 1(g). GO biological process enrichment analysis of DEGs with |log2 fold change| > 1.1 in *PINK1* and *PRKN* mutant DA neurons versus control neurons. The table displays significantly upregulated terms with stats and enrichment analysis results displayed as in Supplementary Table 1(g).

**Supplementary table 1(h)**					
Pathway	Total	Expected	Hits	P.Value	FDR
**Central nervous system development**	784	8.29	21	7.72E-05	0.0356
**Brain development**	559	5.91	17	8.69E-05	0.0356
**G2/M transition of mitotic cell cycle**	150	1.59	7	0.00107	0.292
**Epidermal growth factor receptor signaling pathway**	167	1.76	7	0.00198	0.4
**Regulation of neurogenesis**	444	4.69	12	0.00266	0.4
**Microtubule_based movement**	179	1.89	7	0.00293	0.4
**Regulation of axonogenesis**	104	1.1	5	0.00491	0.575
**Regulation of neurotransmitter levels**	157	1.66	6	0.00645	0.661
**Regulation of Rho protein signal transduction**	177	1.87	6	0.0113	0.868
**Adenylate cyclase_inhibiting G_protein coupled receptor signaling pathway**	45	0.476	3	0.0119	0.868
**Developmental growth**	290	3.06	8	0.012	0.868
**Regulation of Ras protein signal transduction**	302	3.19	8	0.015	0.868
**Neuropeptide signaling pathway**	90	0.951	4	0.0152	0.868
**Interphase of mitotic cell cycle**	435	4.6	10	0.0172	0.868
**Anatomical structure formation involved in morphogenesis**	2090	22.1	32	0.018	0.868
**Adenylate cyclase_activating G_protein coupled receptor signaling pathway**	53	0.56	3	0.0184	0.868
**Transmembrane receptor protein tyrosine kinase signaling pathway**	782	8.26	15	0.0184	0.868
**Interphase**	443	4.68	10	0.0193	0.868
**Microtubule_based process**	516	5.45	11	0.0211	0.868
**Fatty acid biosynthetic process**	151	1.6	5	0.0221	0.868
**Regulation of Rho GTPase activity**	101	1.07	4	0.0222	0.868

**Neurotransmitter secretion**	108	1.14	4	0.0276	1
**Cell migration**	1050	11.1	18	0.0294	1
**Regionalization**	353	3.73	8	0.0339	1
**Heterophilic cell_cell adhesion**	29	0.306	2	0.0374	1
**Adenylate cyclase_modulating G_protein coupled receptor signaling pathway**	120	1.27	4	0.0384	1
**Regulation of Ras GTPase activity**	176	1.86	5	0.039	1
**Regulation of cell growth**	302	3.19	7	0.0414	1
**Establishment or maintenance of cell polarity**	123	1.3	4	0.0415	1
**Vasculature development**	652	6.89	12	0.0437	1
**Negative regulation of intracellular transport**	77	0.814	3	0.048	1
**Membrane lipid biosynthetic process**	130	1.37	4	0.0491	1
**Actin filament_based movement**	78	0.824	3	0.0496	1
**Chromosome condensation**	34	0.359	2	0.0499	1

Supplementary Table 1(h). GO biological process enrichment analysis of DEGs with |log2 fold change| > 1.1 in homozygous *PINK1* mutant DA neurons versus control neurons. The table displays significantly upregulated terms with stats and enrichment analysis results displayed as in Supplementary Table 1(h).

**Supplementary table 2 (a)**	
**Dysregulated genes with log2FC>1.1 and FDR<0.05 for homo. and hetero. PINK1 mutant DA neurons**	
**GeneId**	log2FC
**RP11-244F12.2**	4.651297881
**RP11-402D21.2**	-5.503060723
**IGFBP7-AS1**	7.547126514
**AKAP13**	-4.458268188
**LRBA**	-4.35555648
**PAPPA-AS1**	3.533969977
**PCSK7**	6.090319612
**OFD1**	-4.660539109
**KRT8**	4.831400318
**RP11-205M5.3**	3.060759465
**ACTA2-AS1**	4.629763426
**PRICKLE3**	4.132042973
**RP11-482D24.3**	-4.95625342
**RP11-6O2.4**	-2.522414018
**LOXL1-AS1**	4.859459185
**RP5-998C11.1**	-3.339605856
**CTD-2369P2.8**	5.791613506

**XXyac-YX65C7_A.2**	-2.551264314
**TPM1-AS**	2.621799174
**SMC5-DT**	6.390453585
**CCDC152**	2.530936066
**BRF2**	4.003757839
**TCP1**	-3.34661759
**NKX2-2-AS1**	-9.124538173
**CTD-2636A23.2**	-3.51666303
**CTC-327F10.4**	7.06161278
**TMEM120B**	2.484677449
**LCORL**	-2.953766005
**ADCY3**	-2.182311088
**RP11-307C12.12**	2.447887903
**SRFBP1**	3.727817718
**NR2F1-AS1**	-3.228269554
**SAP30L-AS1**	2.886466815
**HPS3**	5.847643699
**C8orf88**	3.523412109
**CTC-463N11.4**	-2.195253979
**FEM1B**	6.106772772
**INPP5A**	-5.891596683
**POLR2B**	5.937624883
**CLCC1**	-2.631241305
**RP11-234G16.4**	-3.844382189
**RP11-482D24.2**	-4.517316889
**SMPD3**	3.131057142
**MYL12-AS1**	2.222704794
**PRC1-AS1**	-2.541092427
**GRM3-AS1**	-3.018101625
**FSIP1**	4.080221337
**SOX21-AS1**	-4.920333546
**RP11-427I6.5**	-2.414391746
**ZEB2-AS1**	-4.41443269
**AP001469.5**	-2.808533458
**RP11-299G20.2**	3.230059379
**TGFB3-AS1**	4.383992652

**BCL6-AS1**	3.546483237
**ZDHHC8**	-4.686868471
**LINC00621**	-4.181228446
**RP11-272L14.3**	-1.60808196
**MCF2L2**	-2.022392628
**TMEM139-AS1**	6.052489617
**RP11-893F2.5**	4.041079975
**CTB-102L5.9**	2.647990924
**CAPN12**	2.153951097
**HLCS**	3.608407747
**RP11-307B6.3**	5.659541107
**RP11-435O5.5**	-4.135269746
**PLEKHG3**	3.055514292
**CALD1**	3.040464727
**MMP24OS**	2.571069066
**SEMA6A-AS1**	-2.237410323
**TPT1-AS1**	-2.693380841
**BLCAP**	6.671705249
**SPAG4**	3.400827644
**VIM-AS1**	-1.632958698
**BMP6**	1.778401567
**RP11-572N21.1**	-6.617959609
**RP11-211G3.3**	2.80476251
**RP11-284F21.7**	-5.682406862
**KRT7-AS**	7.698080258
**C12orf65**	-1.809120857
**RP11-233G1.8**	7.063520088
**FIP1L1**	3.196283073
**RGMB-AS1**	-3.322667495
**HADHA**	-3.258201297
**LINC01572**	-5.581974164
**COL1A1**	5.343164469
**RP11-92C4.3**	2.911700291
**ZFYVE16**	1.221545297
**FADS1**	-2.761686879
**RP11-510J16.5**	5.666101368

**NECAB1**	1.805064632
**RP11-124N14.3**	-1.557316697
**ITFG2**	-3.114762082
**THBS1-AS1**	3.591443753
**DENND1A**	-2.055299124
**RP5-955M13.4**	-3.060008165
**RBIS**	-1.674696437
**IDH3A**	-3.334365935
**RP11-394B2.1**	-1.254197074
**AFDN-DT**	-4.498321932
**NCMAP-DT**	1.553736409
**AC004538.3**	-3.216915993
**ZNF483**	3.161531118
**AATK**	-1.761298956
**ALG12**	-2.658225648
**GDF5-AS1**	4.612456116
**LUC7L3**	2.304069129
**CERT1**	-2.272834449
**GS1-72M22.1**	-2.399059501
**SEMA4D**	-2.094866516
**EPB41**	3.419026594
**RP11-814P5.1**	6.372386571
**RP11-629N8.5**	3.985299575
**MYLK-AS1**	2.969855041
**C16orf70**	2.62676335
**RP11-25K24.3**	6.902811527
**MARCKS**	-2.078979167
**GSTM3P1**	-5.331968168
**SLC25A26**	-1.831499337
**RP11-59C5.3**	-1.502646994
**MFF-DT**	2.487959107
**RNF141**	3.844302876
**SPTB**	3.15464005

**RP11-74H8.1**	-1.779716635
**TMEM186**	-1.867617524
**TMEM51-AS1**	-2.111581705
**TMEM97**	1.486311874
**CTD-2215L10.1**	-2.844192762
**CELSR2**	-3.785193709
**LINC00404**	-6.146163793
**MTHFD2**	2.78443966
**RP11-342D11.2**	-1.825991061
**RP11-422N16.3**	-3.081611709
**HHIP-AS1**	-6.618089015
**NOC2L**	2.701779251
**GS1-34D21.1**	-2.285520808
**TUBB1**	-2.019879815
**BRI3**	3.370173143
**FAM234A**	2.287757461
**RTRAF**	3.160430949
**RP11-96D1.8**	2.017618971
**GAS6-AS1**	2.204966347
**SCYL3**	-1.458904063
**HNRNPA2B1**	2.297566378
**CARM1**	1.279205339
**RP11-732A19.6**	-2.151168391
**NCAM1-AS1**	-1.40001536
**POLG**	-2.847395843
**RP11-331G2.8**	3.559736259
**MBNL1**	2.652858116
**RP11-242G5.1**	5.777891654
**SNHG8**	3.871369263
**PTRH1**	2.994135652
**LINC00928**	-3.229754317
**P4HA2-AS1**	2.847406576
**EPN2**	1.720812587
**CTD-2015H6.3**	1.370801491
**CTD-2175A23.1**	4.413547492

**SKP2**	-1.226981447
**RNF217-AS1**	-1.841288874
**IDI2-AS1**	-1.945436918
**CTD-2207P18.1**	5.943168644
**NDUFB9**	-1.536232923
**FAM172A**	-4.410777409
**CFAP44**	-2.549627603
**AC009133.15**	-1.602745181
**PLEKHG1**	3.653212118
**RP11-192H23.5**	-1.780188455
**JAKMIP2-AS1**	-1.372137695
**BBIP1**	1.161960276
**HELLPAR**	4.473832688
**CSP2**	2.597096732
**PDIA3**	2.189208272
**AC008746.3**	-2.978084128
**SLC7A6OS**	2.040791247
**SGPP2**	-1.825955452
**CLIC6**	4.948334333
**MTERF2**	2.854001119
**FLJ16779**	3.16522387
**RP11-710F7.2**	3.8774197
**FGFBP3**	-3.847212163
**CYP1B1-AS1**	2.631079873
**GCSAM**	3.611143966
**ANKRD45**	6.135237411
**PSMG4**	1.197842416
**MAPK7**	2.379171056
**RP11-344B2.2**	-1.714834469
**DCAF1**	-1.394777104
**MTERF4**	2.615009218
**TBCE**	1.309606902
**RP11-720L2.4**	4.946787363
**CTB-147C22.8**	5.294687786
**UBE2N**	3.54388619
**LRRC8C-DT**	-1.748301246

**BDNF-AS**	2.827124332
**IFT20**	-2.717208282
**APBB2**	2.498309303
**DCHS1-AS1**	-1.471624536
**PXN**	-1.89944989
**TRIM59**	-1.811312705
**RP5-872K7.8**	-5.206767838
**SHLD2**	-1.612806019
**RP11-326A19.5**	1.416048516
**RP4-555D20.1**	-2.457751418
**TBPL1**	3.773787544
**C17orf100**	2.326484105
**A1BG-AS1**	-1.439995795
**SPRYD4**	1.391416247
**ZNF710-AS1**	-1.364691393
**RP11-12J10.4**	-2.105544379
**ERAP1**	1.238933779
**RPL10**	1.490356941
**ANKS1B**	1.257246134
**AE000658.22**	-1.358493587
**KCNK12**	-1.823987397
**ACTC1**	4.654569254
**RP11-17A4.2**	4.062782672
**SLC18A2**	1.128970079
**AC004160.4**	-3.430407431
**RP11-325J6.2**	-2.652944005
**CTC-558O2.2**	2.106702736
**RP11-296L22.8**	-1.642164812
**ACTN4**	3.162636409
**MT-ND1**	2.25838626
**MARCHF7**	1.681470859
**GSE1**	-1.900557823
**STMP1**	5.395355564
**YIF1A**	-1.74438348
**OR7E12P**	2.515395303

**FN1**	-1.543344684
**CTD-2561J22.5**	3.109132294
**AC008067.2**	-1.988348649
**MT-TY**	1.709966396
**WDFY3-AS1**	-1.430863159
**RP1-138B7.5**	1.564301978
**RP11-547I7.1**	-5.32804817
**SPRN**	-1.186677058
**CARD14**	2.008357653
**WDR3**	2.502504277
**EXOC6**	-5.73355803
**TIGD7**	-1.358135058
**TPM1**	2.694505395
**RP13-238F13.3**	-4.225953038
**INF2**	2.499013044
**BAIAP2L2**	1.249398829
**ANGPTL6**	1.68092001
**LINC02458**	4.617234681
**CTD-3094K11.3**	-2.95917999
**BID**	1.291955304
**RP11-810O3.2**	4.525615264
**RP5-881L22.5**	2.49964706
**RP11-567G24.3**	-2.343486491
**MT-ND2**	2.085101286
**TEX26-AS1**	4.379046123
**CTB-176F20.3**	1.522661001
**PSMC1**	-1.295093919
**RUSC1-AS1**	-1.44836138
**FMNL1**	1.939661467
**RP11-154J22.1**	1.139378808
**LOXL1**	3.505330814
**IPPK**	-1.438297371
**CTD-3193O13.1**	4.452011033
**RP11-204E9.3**	-1.678528825
**TPM2**	4.472879691
**RP5-991G20.1**	-1.477817691
**TCFL5**	4.074194615
**HSD17B12**	2.247554189

**OIP5-AS1**	-2.264981091
**ARHGAP44-AS1**	4.852165292
**FLNB-AS1**	1.563746044
**SPON1-AS1**	-5.735314374
**AC004490.1**	-1.495399626
**TNFRSF25**	-3.954952811
**POLDIP2**	1.30631653
**ANKRD37**	-1.2225813
**MIR1282**	1.215072189
**AF127577.10**	1.42485938
**RP4-742C19.13**	1.364524527
**CLDN4**	3.974995355
**KIF7**	-4.254118663
**SPIDR**	2.897375771
**RP11-284H19.1**	-2.42150937
**RP11-542C16.1**	1.812533585
**MT-TC**	1.711811367
**NAV2**	2.025350274
**CTD-2510F5.4**	-3.796743683
**RP11-845C23.3**	-1.570558717
**SREBF2-AS1**	-1.294293144
**MGC32805**	-1.91967589
**NUDT3**	2.422525219
**SNCA**	4.769170042
**AKAP9**	-2.009549958
**CTSB**	-2.188533257
**CTB-13F3.1**	3.10275519
**RP11-787I22.3**	1.30619933
**NMT1**	2.876525056
**NTPCR**	1.249522396
**SPATA1**	2.036408291
**RP11-234G16.6**	-2.971113624
**MYL9**	4.968151326
**SLC13A5**	4.15771612
**RP13-129E14.1**	3.362289637
**STK25**	1.251575667
**KLRK1-AS1**	4.547588632
**LMX1A-AS2**	2.67069249
**PRUNE2**	2.658423042

**RP11-6G22.1**	1.750690602
**ACAD8**	2.215263362
**ADCY7**	-1.115288035
**ANKS1A**	3.486776185
**RP11-1055B8.8**	-2.195453112
**BASP1P1**	-5.389764999
**LDLRAD2**	3.67394179
**RP11-360L9.7**	-1.73988748
**RP11-152H18.3**	1.209668119
**RP11-361L15.5**	1.656640915
**SYT1**	2.649246477
**ZNF142**	1.384265054
**CYP2U1-AS1**	2.208885466
**RELL2**	1.591079758
**RP5-858B6.3**	1.702078403
**NKTR**	1.857457215
**RP1-18C9.3**	-1.231903181
**GATA3-AS1**	-4.259925736
**RP11-81K13.1**	1.387045132
**LIF-AS2**	3.640657428
**CTC-296K1.3**	3.830809818
**DLX1**	-5.397179119
**NR2F2**	-2.966096679
**RP11-342C2.2**	-2.935866803
**RP11-353N14.7**	-2.127216101
**AC009133.21**	-1.334203625
**SWT1**	2.577158878
**CDR1**	-1.735945068
**CLOCK**	1.895581315
**RP5-1112D6.8**	1.241328633
**HDDC2**	4.734973998
**RP1-18D14.7**	-5.284856966
**NR2F1**	-2.006759178
**RP4-541C22.5**	-2.296808887
**VPS35L**	-1.499077965
**RP11-347D21.5**	5.381982282

**USP22**	2.122515199
**FAM169A**	-1.18046208
**CTD-2240E14.4**	1.566779198
**RNF169**	1.118737089
**RP4-734G22.3**	-1.616976931
**YIF1B**	5.551261641
**RP11-226E21.4**	-1.15577993
**IFNWP19**	3.585537663
**RP4-613B23.8**	1.146041684
**PC**	-1.103208289
**MUC3A**	2.700309466
**LRRC41**	-2.089041436
**CENPN-AS1**	-2.097597544
**GFAP**	-1.489939505
**MYH9**	2.745661842
**WDFY3-AS2**	-1.768354762
**AC083884.8**	-1.157683109
**CASK-AS1**	-1.655476002
**RIOX2**	-1.176058741
**PLD4**	2.162088043
**TARID**	3.809008659
**RP11-463O12.3**	-1.218505403
**PHKG1**	1.134017056
**RP11-149P14.2**	-1.701417094
**RP11-284F21.10**	-4.120854843
**AC053503.6**	-3.229657207
**MAP1B**	-1.264951477
**HCFC1R1**	2.127659303
**RP11-113H14.3**	-2.051874464
**TXLNA**	1.596677638
**ENSG10010137683.1**	-5.061159999
**BBOX1**	3.662364868
**RHCG**	-4.372430373
**CTD-2325A15.5**	2.398628732

**PLAC9**	3.78228432
**RP11-6E9.4**	3.234266666
**SCGB3A2**	4.683069105
**ZNF461**	1.859441997
**STAT1**	1.723754931
**CTD-2555O16.4**	-1.305653484
**RP11-23P13.6**	1.677493295
**UBXN6**	-1.531502929
**PPIAP53**	1.731327568
**GPR132**	4.760499353
**AC008440.5**	1.145990502
**XXbac-B562F10.11**	-1.525603246
**HOTAIRM1**	-5.146298549
**SHROOM3-AS1**	1.619905501
**CTD-2265O21.3**	4.266088649
**ICMT**	1.793653685
**LINC00334**	1.180492492
**RP3-326L13.2**	-4.959755324
**MIR34AHG**	1.699886342
**COL4A2-AS1**	2.546743499
**EYA4**	4.214103174
**RP11-261N11.8**	-1.910298409
**RP11-460I13.2**	4.62046261
**RP5-850O15.3**	4.548536442
**CDKN2B-AS1**	3.284403053
**CLDN22**	1.785680435
**RP11-544A12.4**	-1.466442173
**RP1-37C10.7**	2.5082957
**NUP153-AS1**	-1.456866449
**RP5-1024G6.5**	-1.170846026
**AC005625.1**	2.631950198
**RP11-25K19.1**	-2.467493184
**RP11-945C19.4**	-1.500623417
**UQCC2**	2.294120706
**HS1BP3**	3.170850394
**RP11-103J8.1**	-1.586354702
**FABP7**	-3.504006187

**CTC-250P22.2**	-1.709400265
**RP11-4N23.1**	-2.173367523
**RP11-667F14.1**	2.430177236
**AC003099.2**	4.927350308
**ZNF276**	-1.204492213
**SPARC**	2.416817799
**RP11-666A8.9**	3.932759469
**CARHSP1**	1.444819497
**KLC1**	-1.314289261
**RP11-275G7.2**	-2.294569296
**RP11-95F22.1**	-1.724122082
**RP11-981G7.6**	2.884754814
**CTC-518P12.6**	1.108437058
**RP11-300E4.2**	-1.448702299
**CCDC13-AS1**	1.507719249
**CBX5**	3.844913709
**PBXIP1**	2.2540838
**INTS8**	-2.380700746
**RP11-830F9.5**	-1.708503919
**ZMYM4-AS1**	-1.610552107
**DINOL**	2.515826596
**CCNC**	1.46122178
**KRR1**	2.039677507
**VAX1**	4.880430082
**LIPA**	2.866111918
**CCN2**	3.241405879
**ST7L**	3.064135791
**GCNT1**	1.56999192
**SPATA46**	-2.437624609
**IGFL2**	4.961775385
**GULP1**	3.026230561
**RP11-34F13.2**	-3.51877649
**CCDC183-AS1**	1.387212253
**SMAP1**	-2.155489624
**AC007405.8**	-2.191296333
**CLMAT3**	2.407169348

**RP11-379K22.3**	-1.215100401
**RP11-794P6.6**	3.785142233
**ARSG**	1.308999059
**ELF3-AS1**	1.119367736
**STRADA**	-1.38765132
**LHX5**	-3.401396151
**RP11-265D17.2**	2.998775125
**RPL32P32**	4.782883109
**GRM5-AS1**	-3.024646028
**RP11-375N15.2**	-1.22024056
**NUP62**	-2.643714644
**SMARCC2**	1.617620565
**RP3-330O12.5**	2.921676178
**RP11-381K20.2**	4.750869872
**FAS**	2.013678745
**RP11-435D7.3**	-2.924043112
**ENG**	2.237735843
**DLD**	2.679933695
**MAP3K14**	4.362572084
**XACT**	3.856443549
**RP11-144F15.1**	-1.803133904
**RP3-413H6.3**	4.038934826
**NDUFA4L2**	-2.387813593
**ZEB2**	-2.82026811
**HDAC9**	3.217265877
**FBXO25**	1.505834212
**YPEL4**	-1.762930345
**NFATC3**	3.953345582
**BAZ2A**	1.953260958
**XX-15A10.1**	1.630872609
**KNOP1P5**	4.319902887
**KAAG1**	-2.229391962
**NPB**	-1.698536491
**RP11-626E13.1**	-1.695921208
**TRPA1**	4.06613257
**KATNA1**	2.059718026
**DELEC1**	-3.024116513
**RP11-815J21.2**	-1.32662472

**RP11-156L14.1**	2.725437727
**COL5A1**	3.001222875
**HOOK2**	1.762073823
**OPN1SW**	1.583449321
**ERCC2**	3.465708205
**ZBED5**	1.543206833
**MARS1**	-2.134845221
**MEIS1-AS2**	-3.389715857
**RP4-614O4.13**	1.880463304
**NSUN7**	1.49805064
**SCG2**	2.413898135
**RP11-5O23.2**	-1.31034761
**NEAT1**	3.99158167
**PCAT1**	2.532501356
**ERBIN**	-3.652247382
**KRT18**	3.675133385
**ASB16-AS1**	4.035193852
**RP11-1151B14.4**	1.767093414
**ENSG10010137930.1**	-4.707305644
**UROD**	-2.028988431
**KLRF2**	3.439089709
**CCND1**	1.118505353
**RP11-530C5.1**	1.399570913
**AF196970.3**	-1.761628676
**DNAJC11**	-1.340736503
**GNB4**	1.357538164
**RP11-864I4.1**	1.74149195
**KRT8P26**	3.095323631
**NES**	-2.519464857
**RGS11**	1.354590486
**PLEKHJ1**	-1.440885633
**JMJD4**	1.338363058
**METAP1D**	-4.49414034
**HEPN1**	-2.465217641
**LINC02029**	1.971472844
**RP11-860B13.3**	-1.128286426
**AC022007.5**	-1.202242898

**KIAA1614**	-<br>1.246812674
**AC005943.6**	-<br>1.411512744
**RP11-12M5.3**	3.969876411
**AC009480.3**	-<br>1.876067341
**C9orf16**	-<br>1.258518097
**LINC00602**	1.641820493
**PTK6**	1.309751309
**LTO1**	1.719287596
**RP1-259A10.2**	2.83431569
**MMP24**	1.209127664
**FZD4**	1.703356675
**LINC02139**	3.945537165
**KAZN**	1.954563076
**TMEM245**	3.281769599
**RP1-261G23.7**	-1.13162442
**D2HGDH**	3.428387073
**NUDCD2**	1.121071512
**TSSK2**	-<br>1.498008865
**AC012363.4**	-<br>1.240254879
**RP11-85O21.5**	-<br>3.072425553
**RP11-301N24.6**	2.385975216
**AC024592.9**	3.271132074
**PDXP-DT**	2.236597938
**HDLBP**	1.321027625
**SHPRH**	1.6223293
**PTN**	-3.14011867
**PSMF1**	1.394617315
**CPNE1**	2.868272484
**TLCD3A**	-1.37396539
**RP11-196H14.2**	-<br>2.135850072
**IFNLR1**	3.352251365
**SGO1-AS1**	-<br>3.278470618
**RP4-694A7.2**	-<br>3.463458649
**CCDC103**	-<br>3.357756376
**MLXP1**	2.671856819
**TKFC**	1.254973673
**ERMP1**	1.745418052
**RP1-125I3.2**	1.140075496
**FSTL1**	2.447177251

**THUMPD1**	3.60268798
**LIMS2**	-3.554554964
**RP11-33O4.3**	-1.546817991
**VIM2P**	-1.920871291
**RP11-90C4.1**	4.071429385
**PMM1**	1.902817769
**CDCA7L**	2.498877782
**IGFL2-AS1**	4.069281576
**RP1-69D17.4**	-1.954945886
**RP11-70C1.3**	3.102931492
**CTD-3193O13.12**	2.425195395
**SNRPD2**	2.367537596
**AFG3L2**	1.161994504
**RP11-265N6.2**	-1.127857589
**RP4-671O14.5**	-2.092372541
**MPEG1**	-2.675345142
**MT-RNR2**	1.562118475
**TRAPPC2**	-1.484998502
**RP3-412A9.16**	1.796681192
**URM1**	-3.833608335
**FKBP14-AS1**	1.433268434
**ZNF789**	1.478099195
**SLC32A1**	-4.089633998
**RP11-1277A3.1**	1.181086105
**KATNAL2**	-1.668089925
**RP13-143G15.4**	2.533520604
**FANCD2OS**	-2.106988877
**NEFH**	2.067351107
**TMPO-AS1**	-1.555568199
**PRR27**	4.495677104
**RP11-481J2.3**	-2.395347834
**NT5DC4**	-3.579804739
**FADS2**	-1.802028471
**TTLL10-AS1**	3.710672653
**RP11-437J2.4**	3.402407583

**RBMS1**	4.017754907
**TACC3**	-1.6688274
**AC005481.5**	-3.463002365
**RP11-213H15.1**	3.493474171
**RP11-780K2.1**	-1.600036512
**GNG12-AS1**	1.303164629
**EXOSC9**	-1.556978108
**AP001189.4**	4.129577412
**RP3-514A23.4**	-4.334821249
**POU3F3**	-3.262896361
**RP11-394J1.2**	2.101824017
**ITGB8-AS1**	-2.960288691
**RP11-152P23.2**	1.485942476
**TUBAP12**	-4.055888662
**MTND6P4**	4.525812354
**PHLPP2**	2.301115426
**NCEH1**	4.244149157
**CTD-2619J13.17**	-1.626398691
**RP11-834C11.11**	4.023247423
**SMIM4**	-1.412910704
**RP11-573D15.9**	1.368434667
**CHRM5**	-2.763399869
**NADK2-AS1**	-1.69527959
**RP11-536C5.7**	-1.363608737
**RPL22**	3.428993175
**RP11-416N2.4**	3.224505699
**RP11-69I8.3**	2.625607189
**AC104532.4**	1.80767135
**PDAP1**	1.411455217
**VIM**	-1.210533919
**PAQR4**	-2.241667052
**TP73-AS1**	3.280037806
**ZNHIT2**	-1.914655703
**RP11-84A19.3**	3.475270698
**CASC19**	3.418654636
**RRBP1**	1.194427595

**CTD-2062F14.3**	1.379698566
**RP1-197B17.3**	3.459481727
**ZSWIM1**	1.346215437
**RP11-49K24.3**	-3.683455136
**DIABLO**	2.01667292
**RP11-446H18.5**	2.003627642
**KMO**	1.264410046
**MTRNR2L8**	3.178899295
**COL4A1**	2.751982811
**RP11-4N23.4**	-1.418192546
**ACTN1**	2.907823374
**MYO5B**	1.659867972
**RP11-615I2.2**	2.753886202
**MDK**	2.449504083
**CNTFR-AS1**	-1.309682503
**CTB-79E8.2**	1.773089567
**LCOR**	-3.091395654
**RP11-102N12.3**	1.665336046
**SPTLC2**	3.911517877
**CPM**	1.314183906
**MAST3-AS1**	1.133535114
**POMGNT1**	1.399222273
**RP11-573D15.8**	-1.529409778
**ZMAT5**	2.415659105
**LINC00867**	-1.320183119
**OPN3**	4.081924375
**MYCL-AS1**	-1.742328541
**RP11-425A6.6**	1.782051757
**PNMA2**	1.406727069
**TRIM37**	-1.308282245
**RP11-10A14.3**	1.7673421
**SIDT2**	2.059232231
**MYO1E**	-2.700250688
**CBR4**	1.368761182
**RP4-784A16.2**	-1.738061625
**FABP6-AS1**	4.165023158
**EDNRB**	-3.973335531
**LINC02487**	-3.205821613

**RP11-517I3.1**	1.214448748
**CYP11A1**	4.145457332
**RP11-245K15.2**	4.153867196
**RP11-1072A3.3**	1.126064889
**RP11-755H23.1**	-1.880678962
**RP11-154I21.1**	-3.614527751
**PIEZO1**	-1.429892104
**CTD-2544N14.3**	3.107125516
**RP11-893F2.15**	3.864144492
**CTD-2015C24.1**	-3.899688316
**MAL2-AS1**	1.630892354
**RP11-42O4.2**	3.364728566
**BET1**	3.594996565
**MTCL1**	3.616140162
**RP11-152K4.2**	1.646495898
**GLB1L2**	-1.30881951
**DAAM1**	1.577958662
**RP11-254F19.5**	3.566871134
**SPC24**	-2.920083861
**IGFBP3**	3.840307103
**AC092597.3**	1.505479991
**TAX1BP1**	1.193756611
**AJM1**	-1.971330088
**ACAP1**	1.1845161
**OPN5**	2.422408775
**MN1**	-2.248210913
**MACROH2A1**	1.570049349
**OGFRL1**	2.238589805
**WDR24**	-1.292226972
**RNF2P1**	-1.986922291
**EAF1**	1.433221992
**ZNF862**	-1.24756872
**RP5-896L10.1**	-2.21739316
**RP11-85O21.2**	-4.210831613
**ZNF718**	3.499581689
**CTB-47B11.3**	1.484758193
**TMEM213**	-1.195460954
**HAS2-AS1**	-2.205028486

**GFM1**	3.164644366
**P2RX6**	2.068666609
**RP11-16C18.3**	-1.191944754
**ATXN1L**	-1.273598126
**C1RL-AS1**	1.281475751
**CXCL14**	3.868245091
**DDX20**	-1.39871083
**CTA-992D9.8**	3.250114123
**PIK3IP1**	1.536090889
**PAH**	-1.988973338
**LINC01917**	-1.376848893
**LPIN3**	2.062551787
**COL4A2**	2.216472407
**C1orf159**	2.31801575
**ALKBH4**	1.261699424
**LYPD1**	-1.677880026
**RP5-994D16.9**	-1.280592023
**IGSF8**	1.543439681
**ACADVL**	-1.336730213
**CTD-2555A7.2**	-4.0653698
**HCCAT5**	-3.035648777
**MARK2P16**	-2.076887121
**TINAG**	2.61652464
**IRF1-AS1**	1.905488452
**RP11-989F5.1**	-1.87363307
**NPR3**	3.756238845
**TBX10**	3.004996836
**NHLH2**	-2.345383402
**TRIP11**	1.505414673
**LRRC7-AS1**	-1.886866064
**MORN4**	3.734755117
**ADCYAP1**	1.798839026
**MNS1**	1.156749574
**RP11-146F11.1**	-1.717507232
**ARHGAP5**	1.560717436
**PTP4A1**	3.330044338
**SIAH3**	3.724458533
**CSN3**	4.038153855

**SHH**	-2.912041329
**LINC01361**	-4.010799143
**UCHL5**	-1.528537805
**RP11-118B23.6**	2.192454408
**SNRPGP10**	-2.266678678
**RP11-435O5.4**	-2.191019789
**RP11-73E17.2**	-2.266542479
**KCNA7**	2.875411484
**PARPBP**	2.966093863
**RP11-379F12.4**	-2.436629195
**ATP8B4**	2.392176336
**RP11-1000B6.9**	-3.736003295
**COA1**	1.873096466
**HRNR**	2.91664407
**RP11-95O2.5**	-1.184456797
**RP1-12G14.7**	1.715844101
**TMED1**	-1.105870756
**RP11-632F7.4**	1.495668751
**CTD-2353F22.1**	-2.593187559
**IL21R**	1.316100364
**RP11-1069G10.1**	1.12800672
**AC022154.7**	-1.285541729
**MYO18B**	3.693362847
**RP11-380I10.4**	-3.973558565
**RP11-369E15.3**	3.934634761
**NAP1L1**	1.223150868
**ERRFI1**	-2.500284544
**GPR17**	3.890421827
**ZNF273**	2.513570696
**LINC02334**	2.41921
**AC002057.2**	1.629118146
**FIBIN**	2.290036283
**RP11-50D9.3**	1.101274826
**DNHD1**	-1.302969192
**MDM2**	3.633808131

**EHBP1**	-2.731462084
**NUDT8**	3.133537961
**CTA-109P11.4**	2.770947253
**SIGLEC10**	-2.991418707
**MKRN3**	-1.979961354
**LA16c-314G4.4**	2.822425502
**RP11-30L15.4**	-1.925226336
**ITGA5**	2.780327535
**FXYD1**	2.635351091
**RP11-108L7.4**	1.223290762
**EFCC1**	-3.395160759
**PPP5C**	1.691948567
**GADD45B**	3.317311326
**ITPR1**	2.056913444
**RP11-95J9.3**	-1.228371929
**RP11-67L14.2**	3.886059869
**ABHD15**	2.670799409
**PPFIBP2**	3.575522926
**RP11-135D11.2**	3.608956914
**ZNF56**	1.893559294
**GAS2**	-1.127477031
**GCC2**	1.58066529
**TEX9**	1.355973578
**CTB-60B18.18**	3.781244598
**RP11-80B9.1**	-1.300289879
**FAM13A**	1.485376593
**ZNF732**	1.729257864
**CMIP**	2.035401861
**EFCAB2**	1.965873492
**AC037445.1**	-1.815294179
**AC079135.1**	-3.680160511
**RP11-1140I5.2**	-1.876495094
**RP11-739B23.1**	-1.29164091
**COLEC12**	3.78557935
**HIVEP3**	3.586618355
**AC068057.1**	-3.157239049
**RP11-627G18.1**	3.10302626
**RP5-966M1.7**	-1.43112056

**C11orf42**	-1.936472705
**TMEM161B-AS1**	-2.50266361
**CTD-2017F17.2**	1.984208678
**RP11-268F1.3**	3.787900287
**EMILIN3**	2.057381895
**CTD-2619J13.9**	-1.465617349
**RAET1G**	-3.189138788
**SKIDA1**	-1.421722414
**PCA3**	-1.450825162
**AF131216.7**	-1.269797789
**SPDYE6**	-2.476656676
**ZP3**	3.551901134
**PPIB**	1.376378711
**RP11-183J19.1**	3.518320994
**BAHCC1**	-2.628024325
**RP11-589G9.1**	3.51455756
**AMTN**	3.834001606
**GPR107**	1.28589686
**RP11-96D1.10**	3.781972975
**ASB8**	-1.189399922
**PKM**	-1.13880643
**CENPS**	2.628234712
**MAML3**	2.522495699
**PLK3**	3.778202979
**RP11-110I1.6**	-1.109677966
**BEAN1**	1.812560221
**DIS3**	-1.178085926
**CTC-563A5.5**	-1.152175239
**LIN7A**	1.479478548
**RNASEH1P1**	1.989110974
**USP45**	1.642482261
**GIGYF2**	1.862049147
**BRAT1**	-1.277441278
**RP11-552F3.9**	-1.818956003
**ANO8**	2.1334199
**GFER**	1.505837547

**RP11-428F8.2**	3.698566978
**DAAM2-AS1**	-1.639566545
**LINC00200**	-1.630664892
**RIMKLBP2**	1.35940387
**MORN1**	3.042766131
**DENND1C**	2.149437683
**MICAL1**	1.721108594
**RP11-35G9.5**	2.846884724
**WIPI1**	1.491111828
**RPSAP44**	1.412648704
**PNISR**	1.500280716
**SLC16A8**	1.254174721
**GAD2**	-3.524905599
**AC000095.9**	-1.164944825
**BMF-AS1**	1.520225461
**INHBE**	-2.692237963
**PITX2**	3.509059628
**RP11-395N17.3**	-2.15111963
**LINC00963**	3.452740268
**UBR5**	1.462069565
**RP11-554D14.4**	3.562813927
**WDR54**	1.599565178
**RP11-588H23.3**	2.417966616
**LINC01798**	-3.106200782
**PBRM1**	1.424732922
**COA3**	1.953678699
**RP11-225H22.7**	3.23020426
**NOXO1**	-1.402605131
**CTD-2282P23.2**	-3.533374117
**EVA1B**	1.331238325
**C10orf95**	1.142133033
**QKI**	-2.029118073
**SPATA7**	-1.233256496
**RP4-686C3.7**	3.068564238
**RP11-319G6.1**	1.519299606
**ZFPM1**	-1.893256989
**PSMB7**	-1.517703797

**JAKMIP2**	-2.864606113
**SPON2**	1.738024571
**TECR**	-2.086771625
**RP11-641A6.5**	3.305256906
**CALB1**	1.751149906
**SYN2**	-2.968806132
**RNASEH2B-AS1**	2.145390795
**RP11-483P21.2**	-1.170138445
**MTFR1**	1.223471426
**ACMSD**	-1.468069395
**POU3F4**	-3.724009066
**RP11-177H13.2**	2.695096142
**RP11-305E17.8**	2.1532452
**RP11-546B8.6**	-3.820481263
**RP1-80N2.4**	2.259560965
**PTPN14**	3.150463881
**MAVS**	1.369304219
**RASSF10**	-2.632046382
**MYT1L-AS1**	-2.207593065
**PEF1**	1.4788993
**RP11-108P20.3**	1.445978751
**HNRNPH3**	1.397316891
**RAB13**	1.702108183
**PAX9**	-2.133354792
**NDP-AS1**	-2.373631536
**FOXD1-AS1**	-2.754029427
**RP11-57H14.2**	-1.607382557
**FOSL2**	3.2281934
**POLR1B**	1.462403038
**DNAJC17**	1.281185216
**LINC02669**	2.800076483
**RP11-269F19.10**	-1.658241571
**ERI2**	2.54618702
**BNIP3P17**	-1.866595248
**RP11-229P13.23**	-1.531626788

**ZNF790**	1.731599692
**TFAP2A-AS2**	-3.681856349
**RP11-766F14.1**	-3.649486825
**LINC00237**	-3.645701006
**RP11-430H10.3**	3.417463004
**TTC23**	-1.947748366
**ZFP14**	2.212705897
**RP5-1166H10.4**	2.199303671
**CTD-2647L4.1**	-1.348251286
**PINK1-AS**	1.867684568
**TMEM80**	1.623454592
**AC007563.5**	-2.428501272
**AC114271.2**	1.26477813
**TAF1C**	1.875593334
**PTBP1**	-1.38015385
**TSPEAR-AS2**	2.538878205
**RNA5SP216**	-1.145117515
**TCEAL2**	2.06561555
**DHRS7B**	-1.631681279
**CAVIN1**	2.76739825
**C2CD6**	1.797778782
**LINC00944**	-3.698491914
**LINC02157**	-2.409450393
**KLF9**	2.201450593
**LPIN2**	2.231513101
**HOATZ**	3.372312158
**TSTD2**	-3.045958285
**NTRK2**	-2.986710505
**DDX54**	1.814355481
**LBX1**	-3.616338568
**RP3-514A23.2**	-3.616344893
**RDM1P5**	1.972134465
**RP11-144I2.1**	3.122994643
**P4HA3**	1.175566184
**SRSF11**	1.206104127
**CCDC107**	-1.835817316

**TAGLN**	1.392829043
**ANKRD28**	1.167136128
**MYRFL**	3.52808243
**RP11-315D16.4**	-1.406358044
**FBXL2**	1.269743018
**RP11-135A24.4**	-1.64884676
**CTB-60B18.12**	3.504459603
**SPATA32**	3.509867384
**SEC62**	1.581259578
**SLIT3-AS2**	1.380148706
**AC005540.3**	1.271738896
**PLXDC2**	2.882469966
**SLC9A7**	-1.333034466
**ZNF436**	1.472493062
**MALAT1**	1.566571192
**MAFK**	1.894837453
**RP11-223P11.3**	-1.103545579
**RP11-981G7.3**	-1.82349215
**CTD-2162K18.3**	1.627420607
**PDK3**	-2.454490205
**APOE**	3.508551227
**SOX2**	-2.413719105
**AC007326.9**	-1.878197728
**CCP110**	-2.016286578
**AC002398.12**	1.252768085
**RP11-33N14.3**	-1.142060935
**PTPRN**	-1.675837908
**RP11-397O8.7**	-2.009169644
**PRKG1**	2.488080468
**RP11-219B4.3**	-2.164305635
**AMT**	2.076412269
**RP3-388E23.2**	1.35424658
**ADGRL1**	-1.128601489
**NDUFV1**	2.129292186
**SIX5**	1.346028635
**MT-CYB**	1.190966057
**RP11-535A19.1**	1.623654157
**RP11-241K3.5**	3.300315986

**KDM8**	-1.825142166
**RGP1**	1.946941861
**UPF2**	2.36772299
**CDH3**	3.26912982
**RP4-789D17.5**	1.465281246
**KLHL34**	-1.806888158
**ISLR**	2.689514938
**VAC14-AS1**	-1.194057959
**RP5-1142A6.9**	1.642598748
**RP5-1055C14.9**	-2.473130895
**RP11-54O7.18**	2.068642461
**CSPG4P12**	2.625258218
**XPO4**	2.441066651
**G3BP1**	1.416190866
**SOX11**	-1.898834487
**ALG1L13P**	1.301797532
**MT-TP**	3.025448044
**RP11-549L6.3**	-1.82305563
**B4GALT1-AS1**	3.500078195
**NTN1**	-1.736717102
**SEPTIN7P14**	1.243126421
**TRPM3**	2.748364174
**ANKDD1A**	1.201969896
**RFPL1S**	2.223455532
**CTD-2008L17.2**	1.331772591
**PPP1R12B**	-1.466350314
**CNN2P1**	2.801356732
**RINT1**	1.452314726
**CYP8B1**	2.801378032
**RP11-370B11.4**	3.452464486
**LINCR-0001**	2.641841407
**LACTB**	1.977479048
**ENAH**	1.148023416
**ID3**	3.006947127
**MEG9**	1.428876499
**LUARIS**	-1.616832618
**TOX**	-2.470280092
**BBS10**	3.405490738
**CHRNB1**	2.331360255
**CYBA**	2.807734094

**AC093390.1**	-2.347523499
**SLC8B1**	2.966664747
**NOP9**	1.730075351
**TBL1X**	1.458393078
**RP11-662I13.3**	-2.513964411
**NOTCH1**	-2.73794731
**KLF2P3**	2.234811186
**GRPEL1**	-1.309639528
**RP11-465B22.3**	1.949454102
**MID1IP1-AS1**	-1.555006496
**RP11-446J8.1**	-1.649394583
**RP11-89N17.4**	2.756187575
**RP11-513M16.8**	-1.15033421
**ACOXL**	1.174282325
**ZBTB20-AS1**	-1.932299441
**ADAM1B**	-1.431626752
**COL3A1**	1.923127663
**ITGAE**	-2.817190249
**RBBP4**	1.975487482
**LINC01132**	3.070302493
**LZTS2**	2.303825831
**MYCNOS**	-1.336627572
**TSHZ2**	2.845395245
**AC000035.3**	3.165095342
**PLSCR5-AS1**	-3.097707828
**RP11-203H19.2**	1.466346216
**ZC3H10**	1.971561038
**ST7**	2.302070787
**RP5-1148A21.3**	3.359009382
**TPR**	2.165370657
**SOX8**	-3.214446871
**LINC00632**	-3.395568622
**PRORP**	-1.69775987
**RP11-221G19.1**	3.188433072
**MAGI2**	1.579168074
**PRPH**	3.385264895
**HSPB7**	3.355232737
**CERNA1**	1.432198838

**RP11-346M10.3**	-2.795995922
**RP11-379K22.2**	-1.94020322
**CDKN2AIP**	1.494202659
**CTA-941F9.10**	2.610249328
**IRF1**	1.332492292
**BLNK**	2.350090749
**LRRC57**	1.172316554
**KRT7**	3.327258871
**OR52W1**	-3.374768377
**HSPG2**	2.482253352
**ATXN3**	1.438440135
**YJEFN3**	1.515019675
**LAMP5-AS1**	-3.370451314
**PPFIA2-AS1**	-1.276500403
**AC104809.2**	3.167297086
**SMTN**	1.950366912
**SLC6A17-AS1**	1.148785065
**MROCKI**	-2.15633742
**SRRM1**	1.636177387
**LDLRAD4**	1.936406661
**PAPLN**	1.267550117
**SLC7A11-AS1**	-1.548389769
**CTD-2555O16.2**	-1.452226081
**RP11-424N24.2**	-1.871862126
**DCDC2**	-2.513364193
**RP11-573M3.3**	1.10599131
**RMC1**	-1.117639484
**SBK1**	-1.557799504
**COL9A2**	2.18148443
**ANO7**	2.711493955
**SOX1**	-3.17493389
**TTR**	2.925508018
**GS1-124K5.4**	3.119871774
**GRM7-AS3**	-3.318777802
**RP11-16E23.5**	2.24021681
**LINC02028**	3.303150407
**UNC5C**	-2.073461056
**MT-TL1**	1.892373695

**BCAS1**	1.94073685
**PRRC2C**	1.198980226
**RP11-53B2.3**	1.785966417
**PTENP1**	-1.82668347
**CDKN1A**	2.570228277
**RECQL4**	-1.609337157
**RP11-257O5.2**	1.282953195
**PRKG1-AS1**	1.508682905
**RP5-881P19.8**	1.641952404
**NDUFC1**	-1.15970244
**AC096574.5**	1.662940645
**C9orf147**	-1.807064831
**NKAIN4**	3.089090867
**SRPK1**	2.159093526
**PPIC-AS1**	2.116698895
**RP3-406P24.5**	1.409166525
**MIR3665**	-2.786844663
**USP32**	3.139666835
**EEF1DP2**	2.682735048
**RP11-344E13.4**	2.206833656
**RP11-605F22.1**	1.276091416
**RP11-286N22.14**	1.144556629
**MGST1**	3.095670318
**CASC11**	1.221930973
**MPHOSPH6P1**	2.726227327
**ZNF575**	1.707177215
**SLC19A1**	1.906037595
**PARVA**	3.078813693
**RP11-400K9.3**	-2.790898206
**LINC00240**	1.414787762
**MST1**	-1.514276315
**TLN2**	2.895828067
**SLC9C1**	3.269604679
**RP3-512E2.2**	-1.305863748
**MAST2**	-1.874431595
**RP11-408H20.3**	3.279319897
**ARRDC3-AS1**	2.408491395
**FRMD3**	2.107197924
**RP11-436D10.3**	-1.103071984
**IFIT3**	1.245435546

**LA16c-395F10.1**	-1.217983601
**PLG**	3.096202745
**EDN1**	3.293179058
**TUBB2B**	-1.183235174
**LINC00960**	1.123836164
**CEP57**	-1.254772701
**RAB6C**	2.313953137
**RDH14**	2.627260858
**SMAD1**	-1.593833164
**RP11-582J16.3**	1.36768088
**LINC01618**	3.299472909
**FAM86B1**	-1.546339343
**CTC-563A5.2**	-1.288177819
**BACH1**	-1.833010777
**STRA6**	3.308388641
**PRPF40A**	-1.283965129
**CTA-38K21.6**	-1.65812049
**C2orf15**	2.635841331
**PCNX2**	1.153986261
**RP11-325L7.1**	-1.179268581
**STK16**	2.597875994
**CTA-363E6.1**	3.22655873
**CDCA4P1**	1.429227493
**ODAM**	3.285492161
**RP11-45M22.3**	-1.258064272
**CCND2-AS1**	1.497407729
**STEAP2**	1.956160913
**GPR148**	2.663477673
**CHMP2B**	2.66274187
**IL1B**	2.671780467
**NRXN2-AS1**	-1.988809638
**CTC-251D13.1**	3.096117075
**RP1-267L14.6**	-1.147317523
**BTBD6**	-2.552072659
**RDH10-AS1**	1.851377694
**RP11-7F17.10**	3.218680947
**AP006621.9**	1.800990944

**RIMS4**	2.374501713
**CD4**	1.45898063
**CEBPD**	1.783942709
**LUCAT1**	-1.101238736
**PCP4L1**	1.897077441
**DNAJB2**	2.004141202
**RPS9P1**	1.944185773
**LRP11**	-2.597145157
**MED14OS**	-1.536323322
**AC012513.6**	1.589866487
**RP11-499P20.2**	-1.378041132
**GZMA**	3.003218295
**CREM**	2.506773753
**AGFG2**	2.457262583
**KLHL14**	2.96745823
**RMST**	3.176096451
**FBXO2**	2.34385161
**SLC25A3**	1.385996912
**KCNMA1**	1.786008886
**SGCA**	2.637364818
**FEN1**	-1.227843715
**CTC-308K20.2**	1.718854355
**LAYN**	3.160452243
**TRIM7-AS1**	2.99848609
**NCOR2**	-1.189885364
**RP11-625L16.3**	-3.187965664
**CHGB**	1.374502539
**ARHGEF16**	2.242996733
**A4GNT**	1.992585702
**SNAI3**	-1.499885193
**FOXP1**	2.090119862
**SHOC1**	2.290960828
**STAC3**	-1.74052391
**UPF3B**	2.345145044
**DUX4L50**	-1.55717758
**RP11-98D18.1**	1.243308019
**RP11-108P20.2**	2.676879543
**RP11-273B20.1**	1.143645815
**RP11-420L9.5**	-1.112737046

**RP11-507J18.2**	-1.782697194
**NAA25**	-2.862985538
**KCTD21**	1.833943207
**TSPEAR-AS1**	2.452812947
**SATB2-AS1**	-2.497018587
**CTB-85C5.2**	2.410559543
**HOXA-AS3**	-3.322889309
**AC063976.7**	1.384554281
**LINC00710**	3.018308853
**PAQR5**	1.108847977
**SLC52A1**	2.959499626
**DCHS2**	-1.840422552
**LIPH**	1.827819285
**UGGT1**	1.577345003
**RP11-635O16.2**	1.268706566
**RP11-1110F20.1**	2.400889991
**ONECUT1**	-3.154994754
**BMP2K**	-1.328274057
**MIR762**	-2.243885969
**PCED1B-AS1**	2.822277938
**SMCR8**	-1.424688612
**RUSF1**	1.520488846
**KLB**	-1.811791586
**ZNF285B**	-1.366714281
**AC010641.1**	2.219763243
**PPIC**	2.285821281
**ARSJ**	2.537989666
**LMX1A**	2.782827559
**PCOLCE-AS1**	1.302764235
**BNIP3P9**	2.661028946
**AF064858.6**	1.897701465
**AL163953.3**	2.916484825
**GATB**	2.914805954
**R3HDM2**	-1.840286705
**CD44-AS1**	-1.913569041
**CABYR**	1.311412561

**SEMA6A**	-1.189194915
**PCDHA5**	1.809240564
**RP11-51B23.3**	2.549766303
**MSX2**	3.125730705
**TENM4**	2.507023821
**PTPRZ1**	-2.94613481
**RP11-469A15.2**	3.11175044
**IST1**	1.792549023
**SSPN**	-1.286284328
**ELF3**	3.109041552
**ACKR4**	1.187603534
**MYC**	2.939027275
**RP1-104O17.3**	-3.125531289
**LINC00973**	2.908785107
**KLF2P2**	2.071950344
**SIX3**	2.920113111
**ADNP**	1.744102738
**RP11-445N20.2**	2.188539893
**SNRPD3**	1.180830453
**CCSAP**	1.464097561
**MYO7B**	2.945061464
**CTD-2020K17.3**	1.635870421
**LRRC46**	1.802836123
**RPS20P4**	-2.226881954
**RP11-128A17.1**	-1.910268251
**UFL1-AS1**	-2.749884712
**NMNAT2**	1.84940416
**LINC01498**	3.114804491
**RP11-619A14.2**	2.566428753
**SUMO1**	2.671629793
**RP11-234O6.2**	-1.744609083
**ELK4**	1.581229842
**RP11-697K23.3**	3.029071899
**PAPPA**	1.61769471
**RP11-177B4.1**	1.376218143
**RP5-1103B4.3**	-1.112566977
**DAZAP1**	1.271842385
**IFITM1**	1.199979241
**GALR1**	-2.718980866

**RP11-431N8.1**	-1.256442393
**CTD-3193K9.4**	2.036038139
**AC068499.10**	1.384424931
**EML2**	2.4524766
**MIR4500HG**	-1.359963458
**LINC01036**	3.015289663
**TMC3-AS1**	2.748130889
**LINC01621**	2.394541191
**EPHA5**	1.130671228
**GLRX5P2**	2.876934466
**OR2AT4**	2.893123682
**IRS1**	-1.323570701
**TNNT1**	2.995911681
**MIR9-3HG**	-2.229121925
**TMEM92-AS1**	3.050233799
**RP11-521O16.2**	2.440674258
**ITGA4**	1.149721914
**SCNM1**	2.075984357
**RP11-394B2.5**	-1.174432214
**RNF19A**	1.633753695
**HEBP2**	1.260603717
**WDR97**	3.075195215
**CTD-3128G10.6**	1.39492729
**DHX8**	1.833742481
**PRSS51**	-1.332789575
**RP1-223E5.4**	1.227754427
**AC004381.9**	3.037782614
**DHRS12**	2.276660342
**BCAR1**	2.353596153
**RP11-317B17.4**	1.342301194
**PRRT3**	-1.347282774
**NKX2-2**	-3.041617821
**TJAP1**	1.722173127
**KIAA1755**	2.543468856
**ZSWIM7**	-1.461865637
**AC000032.2**	-2.014260605
**F5**	1.685366255
**HAS1**	1.850011017
**RP11-344E13.1**	2.292883007

**RP11-567E21.3**	3.102191133
**RCAN3**	2.494984337
**ANAPC11**	-1.758302343
**RGS17**	1.234479706
**LEF1-AS1**	-1.866955807
**RP11-596C23.6**	2.604809545
**HMMR-AS1**	-2.283187681
**CTD-2002J20.1**	-1.706367801
**PRICKLE2-DT**	2.370340947
**RP11-446E24.3**	2.2838889
**HEPHL1**	1.803433769
**ZNF114**	2.980698505
**ANXA2P1**	-2.380009977
**DHDDS**	-1.278149317
**RP11-83B20.1**	1.625548098
**AC005281.2**	2.615996239
**GTPBP2**	1.149413697
**RP11-172H24.4**	2.339026471
**PODXL**	1.702730872
**RP3-395C13.2**	-1.618723783
**USP17L2**	-2.880446981
**FRG1HP**	2.911100037
**RP11-118M9.3**	-2.204136807
**RP11-392A14.9**	1.60529016
**RP5-1029K10.2**	-1.817658416
**ZNF19**	1.625220172
**SLC5A10**	1.192048575
**SOX1-OT**	-3.027101849
**HHLA3-AS1**	2.098150876
**ITM2B**	1.804581359
**ZNF12**	1.226350997
**GAS6**	2.182231444
**CCDC134**	-1.28977044
**RNF139-AS1**	-1.531043861
**ACTR3C**	-2.633428156
**RP11-308D13.5**	2.461341697
**STYXL2**	2.634230788

**AC093690.1**	1.255928225
**UBAP1**	-1.50162601
**CTB-50E14.4**	-2.085638399
**KCNJ10**	1.988716078
**HMGB3P24**	-1.671880474
**IL23A**	1.927128977
**LINC02052**	2.794656281
**RP11-21C4.1**	-3.036450027
**DYNLT4**	1.186186494
**GSDME**	1.569312127
**RP1-69D17.3**	-1.429544271
**LMO7**	2.905993226
**HKDC1**	2.145080216
**RP11-1085N6.5**	2.431311417
**RP3-528L19.1**	2.950137592
**CTB-187M2.2**	-1.111906274
**AGGF1**	-1.952341907
**YBX1P2**	2.77644176
**SBNO1-AS1**	-2.028416912
**PLP2**	1.909590583
**RP11-388M20.2**	2.102136501
**CTB-25B13.9**	2.914149121
**RP11-234G16.5**	-2.628161524
**CAMK4**	-1.394737083
**RP11-993B23.3**	2.586841049
**CYC1**	-1.169446313
**UBN1**	1.648871132
**AL132709.1**	2.437451183
**SLIT3-AS1**	2.923283578
**USP1**	-1.807140447
**SLIT1**	-2.216779725
**ZNF540**	1.55213348
**SH3GL1P2**	2.882294501
**OR5BK1P**	1.765305199
**RP11-183G22.1**	2.892036442
**FER**	1.627085444
**WDR74**	1.355618405
**PDLIM3**	1.388014628

**HSPH1**	-1.310282093
**NF1**	1.390178851
**DCST1**	2.163360598
**ARL8B**	-2.59046196
**CTD-3051D23.1**	2.905138131
**ENSG10010135817.1**	2.543877913
**FLJ12825**	1.791981906
**SPZ1**	2.768453163
**ITGA9-AS1**	-1.599929876
**ACAD9**	-1.201226593
**RBP5**	1.197138115
**RP11-161H23.9**	1.87416506
**RP11-314E23.2**	2.875906529
**VWA2**	1.781780994
**CERS5**	1.587748451
**AF127577.13**	2.151670884
**RP11-51B6.1**	-2.905642698
**ROR1-AS1**	2.877094055
**KIRREL3**	-2.092721997
**RP11-513G11.5**	2.725791553
**ID1**	2.870245518
**NRL**	-1.864274492
**SRPX2**	-1.713571996
**LIF**	2.868699299
**CH17-195P21.2**	2.223168211
**GPR75**	2.15083818
**WNK3**	1.981012433
**AC007750.5**	2.569220965
**RASSF9**	2.868298546
**RP11-227H15.5**	2.256528499
**MEG3**	2.489769645
**SLCO1A2**	2.051475562
**SKI**	1.751440282
**SOX9**	-2.198387527
**MSC**	2.725086925
**AP001066.12**	2.86304649
**RP11-318M2.5**	1.269618708
**CACNA1S**	2.937883262
**RP11-362A9.3**	1.73813971
**FBN1**	2.530790049
**HMGN5**	2.062567135

**SCD**	-2.024748719
**TXNL1**	-1.467153519
**LINC02867**	1.738351137
**WARS1**	-1.985744806
**RP11-248E9.7**	2.875462622
**PARTICL**	2.631321345
**ASPDH**	1.725567424
**RP11-1084A12.2**	-1.105054777
**RN7SKP275**	-2.339347634
**RP11-434D9.1**	2.748489756
**PRMT6**	-1.178257191
**RBBP6**	1.460421938
**CACNA2D3-AS1**	-1.726072177
**LTB4R2**	2.697305779
**COL11A1**	1.784011412
**HOXB3**	-2.671644715
**DSG2**	2.805198273
**PREX1**	-2.540105008
**RP11-114J13.1**	2.879338093
**LINC02225**	2.807718876
**ERFL**	2.680096357
**ZBTB8A**	1.591350194
**AMD1P4**	2.486881791
**GRIK4**	-2.856642311
**NOS3**	1.699993308
**GLB1L3**	1.259974246
**RIN3**	2.257609251
**ARCN1**	2.14942692
**SLC22A17**	1.883736025
**CTC-338M12.5**	2.807547429
**OPRD1**	2.011684897
**CYP2E1**	2.103286227
**U3**	1.137660555
**LRP4-AS1**	-1.260894998
**TFAP2E-AS1**	1.946641663
**RP11-299H22.6**	2.797118507
**RP11-40A13.1**	1.66902881
**TNS1**	2.501753568

**WI2-85898F10.2**	-2.360434445
**PERM1**	1.977104389
**DLX6**	-2.866489168
**TPH1**	2.860590966
**SCRG1**	-1.775317428
**CLDN18**	1.248086945
**CTD-2349P21.11**	-1.691606322
**CTB-33G10.6**	1.404266605
**CYB5R2**	1.464637205
**GPR68**	1.791123464
**RP11-435O5.6**	-2.838406827
**ATXN1**	2.039151465
**RASA4CP**	1.720086841
**GPC6**	1.33442267
**RSRP1**	1.295656484
**TSPAN11**	-1.24239516
**RP11-87H9.4**	2.120764913
**ZNF324**	1.376152497
**ALKBH3-AS1**	-1.422329447
**RP11-672L10.3**	2.531921503
**VAT1L**	1.573646527
**HSPD1**	1.183695845
**IGBP1P1**	-2.376469438
**CAB39**	-1.399256824
**CTC-523E23.5**	1.871293083
**CYP24A1**	2.791997977
**LINC01159**	-2.147336744
**RP4-671O14.7**	1.553044079
**PXYLP1**	2.136978232
**TUT1**	1.234329147
**NPAS3**	-2.24225906
**CTD-2331H12.7**	2.440253261
**ZNF597**	-1.197277155
**KCNT1**	1.353292954
**RP11-347D21.1**	2.797278025
**EVC**	-1.486842455
**TMEM107**	2.63795605
**RP11-502N13.2**	2.17837363

**RP11-235E17.6**	-1.649446809
**RP11-304L19.4**	-1.529033002
**CCDC188**	1.769428101
**RAPGEF3**	1.117241524
**RP11-122G18.11**	2.265575678
**ARL3**	1.957650629
**EDC3**	-1.313418964
**UICLM**	2.680549537
**MAF**	2.110575241
**RNF157**	1.362040414
**INTU**	-2.465763926
**TPM4**	1.309279996
**CHD5**	1.970230537
**IFT22**	1.551443388
**LYST**	2.66239747
**RP11-359M6.1**	2.775028196
**DIP2C-AS1**	1.243091589
**RP11-303E16.10**	-1.292818542
**RP11-64I5.2**	2.640694012
**CCDC121**	1.624106853
**ITGA3**	2.4449669
**PRIMPOL**	-1.556723703
**ZBTB16**	2.363560858
**ARMC8**	1.873571794
**AC090616.2**	-1.203050122
**CHRNA6**	2.740732117
**SCUBE2**	2.613309103
**RP11-15A1.3**	1.848009958
**RP11-71H17.1**	-2.775783304
**ENSG10010136622.1**	-1.344963063
**PNPLA3**	-2.680382252
**MFSD14B**	1.868247145
**LMX1A-AS1**	2.302174345
**LDLR**	-1.485902011
**DHRS3**	2.731969789
**SKP1**	1.441647622
**PDCL3P5**	2.6148881
**YIPF3**	-1.624301464

**C12orf45**	-1.448612501
**ZNF391**	1.331822596
**ATP9B**	2.683450567
**RP11-644F5.11**	-1.609948702
**CTD-2126E3.5**	2.417566576
**ZNF385C**	1.687945698
**FBXO24**	1.69533208
**LINC01668**	-2.34117529
**TTC39A**	1.280001182
**CTD-2639E6.11**	1.115836208
**IQCN**	2.41651691
**RP11-72O9.5**	2.737046343
**PXDN**	1.909320497
**ST8SIA6-AS1**	2.773235725
**EPS8L2**	2.044034925
**RP11-482M8.1**	-2.323581227
**ACBD6**	-2.58306301
**CTD-2516F10.2**	1.470010902
**RP11-98D3.2**	2.597397814
**RP11-389G6.4**	-1.514335964
**RP4-534N18.2**	2.234882148
**AUTS2**	-1.175458952
**MKI67**	-2.364478914
**SHC3**	-1.942674283
**FUBP1**	2.404698009
**TMEM86A**	2.729709099
**LINC02026**	2.698968134
**LSM4**	1.503855301
**NEIL2**	1.609873135
**C6orf223**	1.86056878
**GRK2**	1.37541334
**FLRT2**	1.721868896
**AC097374.2**	1.924687104
**CFLAR**	1.994029441
**PLB1**	2.702744785
**SSTR1**	2.607425241
**RP13-895J2.3**	-1.868248959
**LINC02559**	2.631569355
**PGM2L1**	2.380109201
**PAX8-AS1**	-2.124634059

**GLS**	1.162514931
**AC068196.1**	-1.318037937
**RP11-475G3.1**	1.254148149
**CICP16**	-1.309073614
**SPDYE1**	-1.721264323
**COL1A2**	1.841500981
**RP11-54O15.3**	1.856393337
**RFX7**	2.562996168
**RP5-872K7.7**	-2.419309112
**SGCZ**	-2.21153075
**RAD21**	1.13908889
**TKT**	1.763533038
**TNC**	-2.057433302
**RSRC1**	-1.621467101
**U91328.21**	2.683747043
**RP1-80N2.2**	2.565269123
**RP11-411B10.3**	-1.246108364
**POU3F2**	-1.754763414
**RP5-921G16.1**	-2.645788464
**RP11-154D6.1**	1.102855636
**RP11-798K3.2**	2.711149564
**FRG1CP**	2.242345133
**RP11-973N13.4**	1.11750352
**AC012078.2**	2.671209246
**CEPT1**	2.045341883
**ST3GAL5**	1.538664377
**PDCD6IP**	1.889461107
**GRPEL2-AS1**	-1.199265367
**RP11-318N11.1**	-1.934353692
**SERPINE1**	2.675619235
**CROCC**	1.884703264
**GRHL2**	2.233541006
**SQLE**	-2.161463236
**REST**	1.20501567
**DRG1**	-1.769377541
**KAT8**	1.876013695
**RELT**	2.657529633

**ZMIZ1-AS1**	-1.745086962
**CMTM2**	2.367336457
**RNPEP**	2.555818968
**RPL26P3**	2.682612171
**RP11-713M15.2**	2.71343179
**SYCE2**	-1.193359873
**ITSN1**	-1.122752834
**RP11-195F19.30**	1.942477735
**UBA1**	-2.057949065
**RP4-782D21.1**	2.644952826
**LINC02289**	2.561159041
**FAM43A**	2.120828947
**ZFP69**	1.194868912
**TTC14**	2.018718173
**SLC35G6**	-1.674412776
**MPRIPP1**	-1.159329808
**ATG9B**	2.115170418
**STXBP3**	-2.591509129
**PCOLCE**	2.059379378
**AKR1C1**	-2.125703014
**ITGB7**	2.421632831
**RP11-754H12.1**	-1.771000382
**AC055764.1**	-1.174908161
**PAPSS2**	1.11376578
**FGF13-AS1**	-1.597226605
**EHD3**	1.548929015
**RAI14**	2.337593048
**RP11-564P9.1**	2.626150374
**MT-RNR1**	1.333893897
**AFDN**	-1.227792871
**MMP15**	-2.290241283
**RPL22P24**	-1.130790544
**CTD-2521M24.11**	2.643423125
**RP11-440I14.4**	2.515184121
**HDGFL3**	1.29543536
**PTCH2**	2.016308507

**RIPOR3**	2.131180365
**LRP2**	-2.652155607
**RTEL1-TNFRSF6B**	2.086740818
**OARD1**	-1.128717339
**RP11-802D6.1**	-1.575827344
**LINC02006**	-2.328518459
**RP11-420K8.1**	-1.209841121
**C12orf43**	-1.783001317
**KCNH1**	1.390466523
**MBNL2**	2.569772793
**RP11-313D6.3**	1.426374143
**LINC00565**	2.615155018
**RP11-71L14.3**	2.615155018
**RP11-50D16.4**	-2.164709345
**SNHG16**	2.20762227
**RP11-19B4.2**	-1.846313412
**AC097468.4**	2.609482468
**FZD6**	1.708543438
**RP11-932B15.2**	1.306313864
**RP11-290L1.2**	1.624099611
**NELFCD**	1.605455924
**ABCA3**	-2.119525172
**ZNF747**	1.125612185
**HIP1R**	-1.221585162
**GATA3**	-2.634399988
**RP11-123M6.2**	2.666608505
**STARD5**	1.333387493
**RP11-17J14.2**	1.336779871
**GPR182**	-1.790677
**LINC02020**	2.643640386
**RP11-3L10.2**	2.423443909
**TMEM108**	1.505619217
**ACTA2**	1.569734196
**LINC00683**	2.089326663
**ZNF516-AS1**	-1.439073909
**RPL9P2**	1.667958432
**RP11-107G24.4**	1.386076143
**CCDC80**	1.384810062

**KREMEN2**	-1.168614998
**ATP2C2-AS1**	-1.749323967
**RIF1**	1.796359274
**ZNF510**	2.472733438
**SPATA42**	-2.665003896
**CSNK1G3**	-1.502602776
**PDHA1**	-1.279295735
**SLC16A6P1**	2.475734883
**RPL29**	1.698641087
**SLC30A4**	2.629300285
**TMC2**	1.226099763
**TCAF2**	2.51486773
**PCSK1**	2.194473554
**SSBP4**	1.210313909
**UTP25**	1.973062144
**ITCH**	1.622988148
**SNTG1**	-2.011133421
**IER5L**	2.052201559
**MYO10**	1.443523687
**CTD-2517M22.17**	-1.504590298
**RP11-677M14.7**	-1.875974309
**INPP5D**	2.569567732
**MTND6P22**	2.486039384
**C10orf55**	1.283470183
**CGB7**	2.300675147
**NATD1**	2.002571624
**PEX5L**	1.281273356
**RP13-487P22.1**	1.335041521
**MAP10**	1.854371585
**ADAMTS12**	1.994478918
**XKR6**	1.822900944
**ETS2**	2.604455611
**FOXD3-AS1**	-2.608226859
**WDR87**	1.412835079
**ANO4**	2.563863322
**ASIC3**	-1.318179471
**NFIX**	-2.603194297
**GNAS-AS1**	-1.762233869

**PIGZ**	-1.5837289
**DPP7**	-1.124048606
**LANCL3**	2.017124144
**RTL5**	-1.351241685
**CTD-2335A18.2**	-2.617056303
**STEAP3**	-1.260951859
**RP11-168F9.2**	2.24952201
**GS1-199J3.3**	2.495221469
**PRDX3P4**	1.702747351
**CTD-2081C10.8**	1.965705908
**CAHM**	-2.474162492
**INSYN1**	-2.473061699
**LRRTM2**	1.851640468
**DMAC2**	1.950475232
**RP11-310N16.1**	2.574776862
**PCDHB15**	-2.469823882
**HSD17B6**	-1.231410056
**RP11-756J15.2**	2.560112702
**ZNF781**	2.460123128
**FOXJ3**	1.796846295
**NTF4**	2.248112978
**RP11-128P10.1**	-1.298916029
**RP11-1330D14.1**	-2.122686387
**SPATS2L**	1.179405221
**PGBD4P3**	-1.652314855
**IFT81**	2.100687852
**OPA3**	1.651600518
**BAALC**	1.727867708
**TAF3**	1.666673143
**RP11-66B24.4**	1.488372387
**GOLGA4**	1.29790262
**LRPPRC**	1.979559314
**ZSWIM5**	-2.062497786
**DOK7**	-1.510150704
**LRRC9**	2.322796058
**CTB-109A12.1**	2.25321982
**BIRC5**	2.528786109

**MIR122HG**	2.069225731
**TSEN54**	1.865057685
**RP4-760C5.3**	-2.321671647
**RUNDC3A**	1.471443678
**SNHG22**	2.260694979
**RP11-401P9.6**	-1.707130455
**RP11-478H11.3-001**	-1.921790826
**CDC23**	-2.299360411
**CTD-2515H24.4**	1.562378764
**TIFAB**	2.519380258
**ARSDP1**	2.509421788
**EVA1A-AS**	2.517959832
**FKBP14**	1.142605479
**RP11-582J16.7**	2.517959832
**TMEM199**	2.255413244
**ECHDC2**	1.47824672
**RP4-671G15.2**	2.197833301
**RPS21P4**	1.91376044
**HELZ-AS1**	2.437589473
**RP11-646I19.1**	2.568569578
**ADA**	1.325311105
**RP11-284F21.11**	1.42564194
**RP11-45A17.2**	-1.43017403
**AC104131.1**	-2.604551193
**RPL27A**	1.183314532
**AC067945.4**	1.406828516
**PLA2G4C-AS1**	1.224272343
**RP11-792A8.4**	1.395325912
**EEA1**	2.42810185
**Z82214.2**	-1.411970261

Supplementary Table 2(a): Dysregulated Genes in *PINK1* and *PRKN* mutant DA neurons. This table lists genes that exhibit significant dysregulation (log2 Fold Change (FC) > 1.1 and False Discovery Rate (FDR) < 0.05) in *PINK1* and *PRKN* mutant DA neurons, compared to control neurons. Each gene is identified by its gene symbol, along with the log2FC value.

**Suppelementary figure 2 (b)**	
**Upregulated genes with log2FC>1.1 and FDR<0.05 for homo. and hetero. PINK1 mutant DA neurons**	
**GeneId**	log2FC
**RP11-244F12.2**	4.651297881

**IGFBP7-AS1**	7.547126514
**PAPPA-AS1**	3.533969977
**PCSK7**	6.090319612
**KRT8**	4.831400318
**RP11-205M5.3**	3.060759465
**ACTA2-AS1**	4.629763426
**PRICKLE3**	4.132042973
**LOXL1-AS1**	4.859459185
**CTD-2369P2.8**	5.791613506
**TPM1-AS**	2.621799174
**SMC5-DT**	6.390453585
**CCDC152**	2.530936066
**BRF2**	4.003757839
**CTC-327F10.4**	7.06161278
**TMEM120B**	2.484677449
**RP11-307C12.12**	2.447887903
**SRFBP1**	3.727817718
**SAP30L-AS1**	2.886466815
**HPS3**	5.847643699
**C8orf88**	3.523412109
**FEM1B**	6.106772772
**POLR2B**	5.937624883
**SMPD3**	3.131057142
**MYL12-AS1**	2.222704794
**FSIP1**	4.080221337
**RP11-299G20.2**	3.230059379
**TGFB3-AS1**	4.383992652
**BCL6-AS1**	3.546483237
**TMEM139-AS1**	6.052489617
**RP11-893F2.5**	4.041079975
**CTB-102L5.9**	2.647990924
**CAPN12**	2.153951097
**HLCS**	3.608407747
**RP11-307B6.3**	5.659541107
**PLEKHG3**	3.055514292
**CALD1**	3.040464727
**MMP24OS**	2.571069066
**BLCAP**	6.671705249
**SPAG4**	3.400827644
**BMP6**	1.778401567
**RP11-211G3.3**	2.80476251
**KRT7-AS**	7.698080258
**RP11-233G1.8**	7.063520088
**FIP1L1**	3.196283073
**COL1A1**	5.343164469
**RP11-92C4.3**	2.911700291
**ZFYVE16**	1.221545297

**RP11-510J16.5**	5.666101368
**NECAB1**	1.805064632
**THBS1-AS1**	3.591443753
**NCMAP-DT**	1.553736409
**ZNF483**	3.161531118
**GDF5-AS1**	4.612456116
**LUC7L3**	2.304069129
**EPB41**	3.419026594
**RP11-814P5.1**	6.372386571
**RP11-629N8.5**	3.985299575
**MYLK-AS1**	2.969855041
**C16orf70**	2.62676335
**RP11-25K24.3**	6.902811527
**MFF-DT**	2.487959107
**RNF141**	3.844302876
**SPTB**	3.15464005
**TMEM97**	1.486311874
**MTHFD2**	2.78443966
**NOC2L**	2.701779251
**BRI3**	3.370173143
**FAM234A**	2.287757461
**RTRAF**	3.160430949
**RP11-96D1.8**	2.017618971
**GAS6-AS1**	2.204966347
**HNRNPA2B1**	2.297566378
**CARM1**	1.279205339
**RP11-331G2.8**	3.559736259
**MBNL1**	2.652858116
**RP11-242G5.1**	5.777891654
**SNHG8**	3.871369263
**PTRH1**	2.994135652
**P4HA2-AS1**	2.847406576
**EPN2**	1.720812587
**CTD-2015H6.3**	1.370801491
**CTD-2175A23.1**	4.413547492
**CTD-2207P18.1**	5.943168644
**PLEKHG1**	3.653212118
**BBIP1**	1.161960276
**HELLPAR**	4.473832688
**CSP2**	2.597096732
**PDIA3**	2.189208272
**SLC7A6OS**	2.040791247
**CLIC6**	4.948334333
**MTERF2**	2.854001119
**FLJ16779**	3.16522387
**RP11-710F7.2**	3.8774197
**CYP1B1-AS1**	2.631079873

**GCSAM**	3.611143966
**ANKRD45**	6.135237411
**PSMG4**	1.197842416
**MAPK7**	2.379171056
**MTERF4**	2.615009218
**TBCE**	1.309606902
**RP11-720L2.4**	4.946787363
**CTB-147C22.8**	5.294687786
**UBE2N**	3.54388619
**BDNF-AS**	2.827124332
**APBB2**	2.498309303
**RP11-326A19.5**	1.416048516
**TBPL1**	3.773787544
**C17orf100**	2.326484105
**SPRYD4**	1.391416247
**ERAP1**	1.238933779
**RPL10**	1.490356941
**ANKS1B**	1.257246134
**ACTC1**	4.654569254
**RP11-17A4.2**	4.062782672
**SLC18A2**	1.128970079
**CTC-558O2.2**	2.106702736
**ACTN4**	3.162636409
**MT-ND1**	2.25838626
**MARCHF7**	1.681470859
**STMP1**	5.395355564
**OR7E12P**	2.515395303
**CTD-2561J22.5**	3.109132294
**MT-TY**	1.709966396
**RP1-138B7.5**	1.564301978
**CARD14**	2.008357653
**WDR3**	2.502504277
**TPM1**	2.694505395
**INF2**	2.499013044
**BAIAP2L2**	1.249398829
**ANGPTL6**	1.68092001
**LINC02458**	4.617234681
**BID**	1.291955304
**RP11-810O3.2**	4.525615264
**RP5-881L22.5**	2.49964706
**MT-ND2**	2.085101286
**TEX26-AS1**	4.379046123
**CTB-176F20.3**	1.522661001
**FMNL1**	1.939661467
**RP11-154J22.1**	1.139378808
**LOXL1**	3.505330814
**CTD-3193O13.1**	4.452011033

**TPM2**	4.472879691
**TCFL5**	4.074194615
**HSD17B12**	2.247554189
**ARHGAP44-AS1**	4.852165292
**FLNB-AS1**	1.563746044
**POLDIP2**	1.30631653
**MIR1282**	1.215072189
**AF127577.10**	1.42485938
**RP4-742C19.13**	1.364524527
**CLDN4**	3.974995355
**SPIDR**	2.897375771
**RP11-542C16.1**	1.812533585
**MT-TC**	1.711811367
**NAV2**	2.025350274
**NUDT3**	2.422525219
**SNCA**	4.769170042
**CTB-13F3.1**	3.10275519
**RP11-787I22.3**	1.30619933
**NMT1**	2.876525056
**NTPCR**	1.249522396
**SPATA1**	2.036408291
**MYL9**	4.968151326
**SLC13A5**	4.15771612
**RP13-129E14.1**	3.362289637
**STK25**	1.251575667
**KLRK1-AS1**	4.547588632
**LMX1A-AS2**	2.67069249
**PRUNE2**	2.658423042
**RP11-6G22.1**	1.750690602
**ACAD8**	2.215263362
**ANKS1A**	3.486776185
**LDLRAD2**	3.67394179
**RP11-152H18.3**	1.209668119
**RP11-361L15.5**	1.656640915
**SYT1**	2.649246477
**ZNF142**	1.384265054
**CYP2U1-AS1**	2.208885466
**RELL2**	1.591079758
**RP5-858B6.3**	1.702078403
**NKTR**	1.857457215
**RP11-81K13.1**	1.387045132
**LIF-AS2**	3.640657428
**CTC-296K1.3**	3.830809818
**SWT1**	2.577158878
**CLOCK**	1.895581315
**RP5-1112D6.8**	1.241328633
**HDDC2**	4.734973998

**RP11-347D21.5**	5.381982282
**USP22**	2.122515199
**CTD-2240E14.4**	1.566779198
**RNF169**	1.118737089
**YIF1B**	5.551261641
**IFNWP19**	3.585537663
**RP4-613B23.8**	1.146041684
**MUC3A**	2.700309466
**MYH9**	2.745661842
**PLD4**	2.162088043
**TARID**	3.809008659
**PHKG1**	1.134017056
**HCFC1R1**	2.127659303
**TXLNA**	1.596677638
**BBOX1**	3.662364868
**CTD-2325A15.5**	2.398628732
**PLAC9**	3.78228432
**RP11-6E9.4**	3.234266666
**SCGB3A2**	4.683069105
**ZNF461**	1.859441997
**STAT1**	1.723754931
**RP11-23P13.6**	1.677493295
**PPIAP53**	1.731327568
**GPR132**	4.760499353
**AC008440.5**	1.145990502
**SHROOM3-AS1**	1.619905501
**CTD-2265O21.3**	4.266088649
**ICMT**	1.793653685
**LINC00334**	1.180492492
**MIR34AHG**	1.699886342
**COL4A2-AS1**	2.546743499
**EYA4**	4.214103174
**RP11-460I13.2**	4.62046261
**RP5-850O15.3**	4.548536442
**CDKN2B-AS1**	3.284403053
**CLDN22**	1.785680435
**RP1-37C10.7**	2.5082957
**AC005625.1**	2.631950198
**UQCC2**	2.294120706
**HS1BP3**	3.170850394
**RP11-667F14.1**	2.430177236
**AC003099.2**	4.927350308
**SPARC**	2.416817799
**RP11-666A8.9**	3.932759469
**CARHSP1**	1.444819497
**RP11-981G7.6**	2.884754814
**CTC-518P12.6**	1.108437058

**CCDC13-AS1**	1.507719249
**CBX5**	3.844913709
**PBXIP1**	2.2540838
**DINOL**	2.515826596
**CCNC**	1.46122178
**KRR1**	2.039677507
**VAX1**	4.880430082
**LIPA**	2.866111918
**CCN2**	3.241405879
**ST7L**	3.064135791
**GCNT1**	1.56999192
**IGFL2**	4.961775385
**GULP1**	3.026230561
**CCDC183-AS1**	1.387212253
**CLMAT3**	2.407169348
**RP11-794P6.6**	3.785142233
**ARSG**	1.308999059
**ELF3-AS1**	1.119367736
**RP11-265D17.2**	2.998775125
**RPL32P32**	4.782883109
**SMARCC2**	1.617620565
**RP3-330O12.5**	2.921676178
**RP11-381K20.2**	4.750869872
**FAS**	2.013678745
**ENG**	2.237735843
**DLD**	2.679933695
**MAP3K14**	4.362572084
**XACT**	3.856443549
**RP3-413H6.3**	4.038934826
**HDAC9**	3.217265877
**FBXO25**	1.505834212
**NFATC3**	3.953345582
**BAZ2A**	1.953260958
**XX-15A10.1**	1.630872609
**KNOP1P5**	4.319902887
**TRPA1**	4.06613257
**KATNA1**	2.059718026
**RP11-156L14.1**	2.725437727
**COL5A1**	3.001222875
**HOOK2**	1.762073823
**OPN1SW**	1.583449321
**ERCC2**	3.465708205
**ZBED5**	1.543206833
**RP4-614O4.13**	1.880463304
**NSUN7**	1.49805064
**SCG2**	2.413898135
**NEAT1**	3.99158167

**PCAT1**	2.532501356
**KRT18**	3.675133385
**ASB16-AS1**	4.035193852
**RP11-1151B14.4**	1.767093414
**KLRF2**	3.439089709
**CCND1**	1.118505353
**RP11-530C5.1**	1.399570913
**GNB4**	1.357538164
**RP11-864I4.1**	1.74149195
**KRT8P26**	3.095323631
**RGS11**	1.354590486
**JMJD4**	1.338363058
**LINC02029**	1.971472844
**RP11-12M5.3**	3.969876411
**LINC00602**	1.641820493
**PTK6**	1.309751309
**LTO1**	1.719287596
**RP1-259A10.2**	2.83431569
**MMP24**	1.209127664
**FZD4**	1.703356675
**LINC02139**	3.945537165
**KAZN**	1.954563076
**TMEM245**	3.281769599
**D2HGDH**	3.428387073
**NUDCD2**	1.121071512
**RP11-301N24.6**	2.385975216
**AC024592.9**	3.271132074
**PDXP-DT**	2.236597938
**HDLBP**	1.321027625
**SHPRH**	1.6223293
**PSMF1**	1.394617315
**CPNE1**	2.868272484
**IFNLR1**	3.352251365
**MLXP1**	2.671856819
**TKFC**	1.254973673
**ERMP1**	1.745418052
**RP1-125I3.2**	1.140075496
**FSTL1**	2.447177251
**THUMPD1**	3.60268798
**RP11-90C4.1**	4.071429385
**PMM1**	1.902817769
**CDCA7L**	2.498877782
**IGFL2-AS1**	4.069281576
**RP11-70C1.3**	3.102931492
**CTD-3193O13.12**	2.425195395
**SNRPD2**	2.367537596
**AFG3L2**	1.161994504

**MT-RNR2**	1.562118475
**RP3-412A9.16**	1.796681192
**FKBP14-AS1**	1.433268434
**ZNF789**	1.478099195
**RP11-1277A3.1**	1.181086105
**RP13-143G15.4**	2.533520604
**NEFH**	2.067351107
**PRR27**	4.495677104
**TTLL10-AS1**	3.710672653
**RP11-437J2.4**	3.402407583
**RBMS1**	4.017754907
**RP11-213H15.1**	3.493474171
**GNG12-AS1**	1.303164629
**AP001189.4**	4.129577412
**RP11-394J1.2**	2.101824017
**RP11-152P23.2**	1.485942476
**MTND6P4**	4.525812354
**PHLPP2**	2.301115426
**NCEH1**	4.244149157
**RP11-834C11.11**	4.023247423
**RP11-573D15.9**	1.368434667
**RPL22**	3.428993175
**RP11-416N2.4**	3.224505699
**RP11-69I8.3**	2.625607189
**AC104532.4**	1.80767135
**PDAP1**	1.411455217
**TP73-AS1**	3.280037806
**RP11-84A19.3**	3.475270698
**CASC19**	3.418654636
**RRBP1**	1.194427595
**CTD-2062F14.3**	1.379698566
**RP1-197B17.3**	3.459481727
**ZSWIM1**	1.346215437
**DIABLO**	2.01667292
**RP11-446H18.5**	2.003627642
**KMO**	1.264410046
**MTRNR2L8**	3.178899295
**COL4A1**	2.751982811
**ACTN1**	2.907823374
**MYO5B**	1.659867972
**RP11-615I2.2**	2.753886202
**MDK**	2.449504083
**CTB-79E8.2**	1.773089567
**RP11-102N12.3**	1.665336046
**SPTLC2**	3.911517877
**CPM**	1.314183906
**MAST3-AS1**	1.133535114

**POMGNT1**	1.399222273
**ZMAT5**	2.415659105
**OPN3**	4.081924375
**RP11-425A6.6**	1.782051757
**PNMA2**	1.406727069
**RP11-10A14.3**	1.7673421
**SIDT2**	2.059232231
**CBR4**	1.368761182
**FABP6-AS1**	4.165023158
**RP11-517I3.1**	1.214448748
**CYP11A1**	4.145457332
**RP11-245K15.2**	4.153867196
**RP11-1072A3.3**	1.126064889
**CTD-2544N14.3**	3.107125516
**RP11-893F2.15**	3.864144492
**MAL2-AS1**	1.630892354
**RP11-42O4.2**	3.364728566
**BET1**	3.594996565
**MTCL1**	3.616140162
**RP11-152K4.2**	1.646495898
**DAAM1**	1.577958662
**RP11-254F19.5**	3.566871134
**IGFBP3**	3.840307103
**AC092597.3**	1.505479991
**TAX1BP1**	1.193756611
**ACAP1**	1.1845161
**OPN5**	2.422408775
**MACROH2A1**	1.570049349
**OGFRL1**	2.238589805
**EAF1**	1.433221992
**ZNF718**	3.499581689
**CTB-47B11.3**	1.484758193
**GFM1**	3.164644366
**P2RX6**	2.068666609
**C1RL-AS1**	1.281475751
**CXCL14**	3.868245091
**CTA-992D9.8**	3.250114123
**PIK3IP1**	1.536090889
**LPIN3**	2.062551787
**COL4A2**	2.216472407
**C1orf159**	2.31801575
**ALKBH4**	1.261699424
**IGSF8**	1.543439681
**TINAG**	2.61652464
**IRF1-AS1**	1.905488452
**NPR3**	3.756238845
**TBX10**	3.004996836

**TRIP11**	1.505414673
**MORN4**	3.734755117
**ADCYAP1**	1.798839026
**MNS1**	1.156749574
**ARHGAP5**	1.560717436
**PTP4A1**	3.330044338
**SIAH3**	3.724458533
**CSN3**	4.038153855
**RP11-118B23.6**	2.192454408
**KCNA7**	2.875411484
**PARPBP**	2.966093863
**ATP8B4**	2.392176336
**COA1**	1.873096466
**HRNR**	2.91664407
**RP1-12G14.7**	1.715844101
**RP11-632F7.4**	1.495668751
**IL21R**	1.316100364
**RP11-1069G10.1**	1.12800672
**MYO18B**	3.693362847
**RP11-369E15.3**	3.934634761
**NAP1L1**	1.223150868
**GPR17**	3.890421827
**ZNF273**	2.513570696
**LINC02334**	2.41921
**AC002057.2**	1.629118146
**FIBIN**	2.290036283
**RP11-50D9.3**	1.101274826
**MDM2**	3.633808131
**NUDT8**	3.133537961
**CTA-109P11.4**	2.770947253
**LA16c-314G4.4**	2.822425502
**ITGA5**	2.780327535
**FXYD1**	2.635351091
**RP11-108L7.4**	1.223290762
**PPP5C**	1.691948567
**GADD45B**	3.317311326
**ITPR1**	2.056913444
**RP11-67L14.2**	3.886059869
**ABHD15**	2.670799409
**PPFIBP2**	3.575522926
**RP11-135D11.2**	3.608956914
**ZNF56**	1.893559294
**GCC2**	1.58066529
**TEX9**	1.355973578
**CTB-60B18.18**	3.781244598
**FAM13A**	1.485376593
**ZNF732**	1.729257864

**CMIP**	2.035401861
**EFCAB2**	1.965873492
**COLEC12**	3.78557935
**HIVEP3**	3.586618355
**RP11-627G18.1**	3.10302626
**CTD-2017F17.2**	1.984208678
**RP11-268F1.3**	3.787900287
**EMILIN3**	2.057381895
**ZP3**	3.551901134
**PPIB**	1.376378711
**RP11-183J19.1**	3.518320994
**RP11-589G9.1**	3.51455756
**AMTN**	3.834001606
**GPR107**	1.28589686
**RP11-96D1.10**	3.781972975
**CENPS**	2.628234712
**MAML3**	2.522495699
**PLK3**	3.778202979
**BEAN1**	1.812560221
**LIN7A**	1.479478548
**RNASEH1P1**	1.989110974
**USP45**	1.642482261
**GIGYF2**	1.862049147
**ANO8**	2.1334199
**GFER**	1.505837547
**RP11-428F8.2**	3.698566978
**RIMKLBP2**	1.35940387
**MORN1**	3.042766131
**DENND1C**	2.149437683
**MICAL1**	1.721108594
**RP11-35G9.5**	2.846884724
**WIPI1**	1.491111828
**RPSAP44**	1.412648704
**PNISR**	1.500280716
**SLC16A8**	1.254174721
**BMF-AS1**	1.520225461
**PITX2**	3.509059628
**LINC00963**	3.452740268
**UBR5**	1.462069565
**RP11-554D14.4**	3.562813927
**WDR54**	1.599565178
**RP11-588H23.3**	2.417966616
**PBRM1**	1.424732922
**COA3**	1.953678699
**RP11-225H22.7**	3.23020426
**EVA1B**	1.331238325
**C10orf95**	1.142133033

**RP4-686C3.7**	3.068564238
**RP11-319G6.1**	1.519299606
**SPON2**	1.738024571
**RP11-641A6.5**	3.305256906
**CALB1**	1.751149906
**RNASEH2B-AS1**	2.145390795
**MTFR1**	1.223471426
**RP11-177H13.2**	2.695096142
**RP11-305E17.8**	2.1532452
**RP1-80N2.4**	2.259560965
**PTPN14**	3.150463881
**MAVS**	1.369304219
**PEF1**	1.4788993
**RP11-108P20.3**	1.445978751
**HNRNPH3**	1.397316891
**RAB13**	1.702108183
**FOSL2**	3.2281934
**POLR1B**	1.462403038
**DNAJC17**	1.281185216
**LINC02669**	2.800076483
**ERI2**	2.54618702
**ZNF790**	1.731599692
**RP11-430H10.3**	3.417463004
**ZFP14**	2.212705897
**RP5-1166H10.4**	2.199303671
**PINK1-AS**	1.867684568
**TMEM80**	1.623454592
**AC114271.2**	1.26477813
**TAF1C**	1.875593334
**TSPEAR-AS2**	2.538878205
**TCEAL2**	2.06561555
**CAVIN1**	2.76739825
**C2CD6**	1.797778782
**KLF9**	2.201450593
**LPIN2**	2.231513101
**HOATZ**	3.372312158
**DDX54**	1.814355481
**RDM1P5**	1.972134465
**RP11-144I2.1**	3.122994643
**P4HA3**	1.175566184
**SRSF11**	1.206104127
**TAGLN**	1.392829043
**ANKRD28**	1.167136128
**MYRFL**	3.52808243
**FBXL2**	1.269743018
**CTB-60B18.12**	3.504459603
**SPATA32**	3.509867384

**SEC62**	1.581259578
**SLIT3-AS2**	1.380148706
**AC005540.3**	1.271738896
**PLXDC2**	2.882469966
**ZNF436**	1.472493062
**MALAT1**	1.566571192
**MAFK**	1.894837453
**CTD-2162K18.3**	1.627420607
**APOE**	3.508551227
**AC002398.12**	1.252768085
**PRKG1**	2.488080468
**AMT**	2.076412269
**RP3-388E23.2**	1.35424658
**NDUFV1**	2.129292186
**SIX5**	1.346028635
**MT-CYB**	1.190966057
**RP11-535A19.1**	1.623654157
**RP11-241K3.5**	3.300315986
**RGP1**	1.946941861
**UPF2**	2.36772299
**CDH3**	3.26912982
**RP4-789D17.5**	1.465281246
**ISLR**	2.689514938
**RP5-1142A6.9**	1.642598748
**RP11-54O7.18**	2.068642461
**CSPG4P12**	2.625258218
**XPO4**	2.441066651
**G3BP1**	1.416190866
**ALG1L13P**	1.301797532
**MT-TP**	3.025448044
**B4GALT1-AS1**	3.500078195
**SEPTIN7P14**	1.243126421
**TRPM3**	2.748364174
**ANKDD1A**	1.201969896
**RFPL1S**	2.223455532
**CTD-2008L17.2**	1.331772591
**CNN2P1**	2.801356732
**RINT1**	1.452314726
**CYP8B1**	2.801378032
**RP11-370B11.4**	3.452464486
**LINCR-0001**	2.641841407
**LACTB**	1.977479048
**ENAH**	1.148023416
**ID3**	3.006947127
**MEG9**	1.428876499
**BBS10**	3.405490738
**CHRNB1**	2.331360255

**CYBA**	2.807734094
**SLC8B1**	2.966664747
**NOP9**	1.730075351
**TBL1X**	1.458393078
**KLF2P3**	2.234811186
**RP11-465B22.3**	1.949454102
**RP11-89N17.4**	2.756187575
**ACOXL**	1.174282325
**COL3A1**	1.923127663
**RBBP4**	1.975487482
**LINC01132**	3.070302493
**LZTS2**	2.303825831
**TSHZ2**	2.845395245
**AC000035.3**	3.165095342
**RP11-203H19.2**	1.466346216
**ZC3H10**	1.971561038
**ST7**	2.302070787
**RP5-1148A21.3**	3.359009382
**TPR**	2.165370657
**RP11-221G19.1**	3.188433072
**MAGI2**	1.579168074
**PRPH**	3.385264895
**HSPB7**	3.355232737
**CERNA1**	1.432198838
**CDKN2AIP**	1.494202659
**CTA-941F9.10**	2.610249328
**IRF1**	1.332492292
**BLNK**	2.350090749
**LRRC57**	1.172316554
**KRT7**	3.327258871
**HSPG2**	2.482253352
**ATXN3**	1.438440135
**YJEFN3**	1.515019675
**AC104809.2**	3.167297086
**SMTN**	1.950366912
**SLC6A17-AS1**	1.148785065
**SRRM1**	1.636177387
**LDLRAD4**	1.936406661
**PAPLN**	1.267550117
**RP11-573M3.3**	1.10599131
**COL9A2**	2.18148443
**ANO7**	2.711493955
**TTR**	2.925508018
**GS1-124K5.4**	3.119871774
**RP11-16E23.5**	2.24021681
**LINC02028**	3.303150407
**MT-TL1**	1.892373695

**BCAS1**	1.94073685
**PRRC2C**	1.198980226
**RP11-53B2.3**	1.785966417
**CDKN1A**	2.570228277
**RP11-257O5.2**	1.282953195
**PRKG1-AS1**	1.508682905
**RP5-881P19.8**	1.641952404
**AC096574.5**	1.662940645
**NKAIN4**	3.089090867
**SRPK1**	2.159093526
**PPIC-AS1**	2.116698895
**RP3-406P24.5**	1.409166525
**USP32**	3.139666835
**EEF1DP2**	2.682735048
**RP11-344E13.4**	2.206833656
**RP11-605F22.1**	1.276091416
**RP11-286N22.14**	1.144556629
**MGST1**	3.095670318
**CASC11**	1.221930973
**MPHOSPH6P1**	2.726227327
**ZNF575**	1.707177215
**SLC19A1**	1.906037595
**PARVA**	3.078813693
**LINC00240**	1.414787762
**TLN2**	2.895828067
**SLC9C1**	3.269604679
**RP11-408H20.3**	3.279319897
**ARRDC3-AS1**	2.408491395
**FRMD3**	2.107197924
**IFIT3**	1.245435546
**PLG**	3.096202745
**EDN1**	3.293179058
**LINC00960**	1.123836164
**RAB6C**	2.313953137
**RDH14**	2.627260858
**RP11-582J16.3**	1.36768088
**LINC01618**	3.299472909
**STRA6**	3.308388641
**C2orf15**	2.635841331
**PCNX2**	1.153986261
**STK16**	2.597875994
**CTA-363E6.1**	3.22655873
**CDCA4P1**	1.429227493
**ODAM**	3.285492161
**CCND2-AS1**	1.497407729
**STEAP2**	1.956160913
**GPR148**	2.663477673

**CHMP2B**	2.66274187
**IL1B**	2.671780467
**CTC-251D13.1**	3.096117075
**RDH10-AS1**	1.851377694
**RP11-7F17.10**	3.218680947
**AP006621.9**	1.800990944
**RIMS4**	2.374501713
**CD4**	1.45898063
**CEBPD**	1.783942709
**PCP4L1**	1.897077441
**DNAJB2**	2.004141202
**RPS9P1**	1.944185773
**AC012513.6**	1.589866487
**GZMA**	3.003218295
**CREM**	2.506773753
**AGFG2**	2.457262583
**KLHL14**	2.96745823
**RMST**	3.176096451
**FBXO2**	2.34385161
**SLC25A3**	1.385996912
**KCNMA1**	1.786008886
**SGCA**	2.637364818
**CTC-308K20.2**	1.718854355
**LAYN**	3.160452243
**TRIM7-AS1**	2.99848609
**CHGB**	1.374502539
**ARHGEF16**	2.242996733
**A4GNT**	1.992585702
**FOXP1**	2.090119862
**SHOC1**	2.290960828
**UPF3B**	2.345145044
**RP11-98D18.1**	1.243308019
**RP11-108P20.2**	2.676879543
**RP11-273B20.1**	1.143645815
**KCTD21**	1.833943207
**TSPEAR-AS1**	2.452812947
**CTB-85C5.2**	2.410559543
**AC063976.7**	1.384554281
**LINC00710**	3.018308853
**PAQR5**	1.108847977
**SLC52A1**	2.959499626
**LIPH**	1.827819285
**UGGT1**	1.577345003
**RP11-635O16.2**	1.268706566
**RP11-1110F20.1**	2.400889991
**PCED1B-AS1**	2.822277938
**RUSF1**	1.520488846

**AC010641.1**	2.219763243
**PPIC**	2.285821281
**ARSJ**	2.537989666
**LMX1A**	2.782827559
**PCOLCE-AS1**	1.302764235
**BNIP3P9**	2.661028946
**AF064858.6**	1.897701465
**AL163953.3**	2.916484825
**GATB**	2.914805954
**CABYR**	1.311412561
**PCDHA5**	1.809240564
**RP11-51B23.3**	2.549766303
**MSX2**	3.125730705
**TENM4**	2.507023821
**RP11-469A15.2**	3.11175044
**IST1**	1.792549023
**ELF3**	3.109041552
**ACKR4**	1.187603534
**MYC**	2.939027275
**LINC00973**	2.908785107
**KLF2P2**	2.071950344
**SIX3**	2.920113111
**ADNP**	1.744102738
**RP11-445N20.2**	2.188539893
**SNRPD3**	1.180830453
**CCSAP**	1.464097561
**MYO7B**	2.945061464
**CTD-2020K17.3**	1.635870421
**LRRC46**	1.802836123
**NMNAT2**	1.84940416
**LINC01498**	3.114804491
**RP11-619A14.2**	2.566428753
**SUMO1**	2.671629793
**ELK4**	1.581229842
**RP11-697K23.3**	3.029071899
**PAPPA**	1.61769471
**RP11-177B4.1**	1.376218143
**DAZAP1**	1.271842385
**IFITM1**	1.199979241
**CTD-3193K9.4**	2.036038139
**AC068499.10**	1.384424931
**EML2**	2.4524766
**LINC01036**	3.015289663
**TMC3-AS1**	2.748130889
**LINC01621**	2.394541191
**EPHA5**	1.130671228
**GLRX5P2**	2.876934466

**OR2AT4**	2.893123682
**TNNT1**	2.995911681
**TMEM92-AS1**	3.050233799
**RP11-521O16.2**	2.440674258
**ITGA4**	1.149721914
**SCNM1**	2.075984357
**RNF19A**	1.633753695
**HEBP2**	1.260603717
**WDR97**	3.075195215
**CTD-3128G10.6**	1.39492729
**DHX8**	1.833742481
**RP1-223E5.4**	1.227754427
**AC004381.9**	3.037782614
**DHRS12**	2.276660342
**BCAR1**	2.353596153
**RP11-317B17.4**	1.342301194
**TJAP1**	1.722173127
**KIAA1755**	2.543468856
**F5**	1.685366255
**HAS1**	1.850011017
**RP11-344E13.1**	2.292883007
**RP11-567E21.3**	3.102191133
**RCAN3**	2.494984337
**RGS17**	1.234479706
**RP11-596C23.6**	2.604809545
**PRICKLE2-DT**	2.370340947
**RP11-446E24.3**	2.2838889
**HEPHL1**	1.803433769
**ZNF114**	2.980698505
**RP11-83B20.1**	1.625548098
**AC005281.2**	2.615996239
**GTPBP2**	1.149413697
**RP11-172H24.4**	2.339026471
**PODXL**	1.702730872
**FRG1HP**	2.911100037
**RP11-392A14.9**	1.60529016
**ZNF19**	1.625220172
**SLC5A10**	1.192048575
**HHLA3-AS1**	2.098150876
**ITM2B**	1.804581359
**ZNF12**	1.226350997
**GAS6**	2.182231444
**RP11-308D13.5**	2.461341697
**STYXL2**	2.634230788
**AC093690.1**	1.255928225
**KCNJ10**	1.988716078
**IL23A**	1.927128977

**LINC02052**	2.794656281
**DYNLT4**	1.186186494
**GSDME**	1.569312127
**LMO7**	2.905993226
**HKDC1**	2.145080216
**RP11-1085N6.5**	2.431311417
**RP3-528L19.1**	2.950137592
**YBX1P2**	2.77644176
**PLP2**	1.909590583
**RP11-388M20.2**	2.102136501
**CTB-25B13.9**	2.914149121
**RP11-993B23.3**	2.586841049
**UBN1**	1.648871132
**AL132709.1**	2.437451183
**SLIT3-AS1**	2.923283578
**ZNF540**	1.55213348
**SH3GL1P2**	2.882294501
**OR5BK1P**	1.765305199
**RP11-183G22.1**	2.892036442
**FER**	1.627085444
**WDR74**	1.355618405
**PDLIM3**	1.388014628
**NF1**	1.390178851
**DCST1**	2.163360598
**CTD-3051D23.1**	2.905138131
**ENSG10010135817.1**	2.543877913
**FLJ12825**	1.791981906
**SPZ1**	2.768453163
**RBP5**	1.197138115
**RP11-161H23.9**	1.87416506
**RP11-314E23.2**	2.875906529
**VWA2**	1.781780994
**CERS5**	1.587748451
**AF127577.13**	2.151670884
**ROR1-AS1**	2.877094055
**RP11-513G11.5**	2.725791553
**ID1**	2.870245518
**LIF**	2.868699299
**CH17-195P21.2**	2.223168211
**GPR75**	2.15083818
**WNK3**	1.981012433
**AC007750.5**	2.569220965
**RASSF9**	2.868298546
**RP11-227H15.5**	2.256528499
**MEG3**	2.489769645
**SLCO1A2**	2.051475562
**SKI**	1.751440282

**MSC**	2.725086925
**AP001066.12**	2.86304649
**RP11-318M2.5**	1.269618708
**CACNA1S**	2.937883262
**RP11-362A9.3**	1.73813971
**FBN1**	2.530790049
**HMGN5**	2.062567135
**LINC02867**	1.738351137
**RP11-248E9.7**	2.875462622
**PARTICL**	2.631321345
**ASPDH**	1.725567424
**RP11-434D9.1**	2.748489756
**RBBP6**	1.460421938
**LTB4R2**	2.697305779
**COL11A1**	1.784011412
**DSG2**	2.805198273
**RP11-114J13.1**	2.879338093
**LINC02225**	2.807718876
**ERFL**	2.680096357
**ZBTB8A**	1.591350194
**AMD1P4**	2.486881791
**NOS3**	1.699993308
**GLB1L3**	1.259974246
**RIN3**	2.257609251
**ARCN1**	2.14942692
**SLC22A17**	1.883736025
**CTC-338M12.5**	2.807547429
**OPRD1**	2.011684897
**CYP2E1**	2.103286227
**U3**	1.137660555
**TFAP2E-AS1**	1.946641663
**RP11-299H22.6**	2.797118507
**RP11-40A13.1**	1.66902881
**TNS1**	2.501753568
**PERM1**	1.977104389
**TPH1**	2.860590966
**CLDN18**	1.248086945
**CTB-33G10.6**	1.404266605
**CYB5R2**	1.464637205
**GPR68**	1.791123464
**ATXN1**	2.039151465
**RASA4CP**	1.720086841
**GPC6**	1.33442267
**RSRP1**	1.295656484
**RP11-87H9.4**	2.120764913
**ZNF324**	1.376152497
**RP11-672L10.3**	2.531921503

**VAT1L**	1.573646527
**HSPD1**	1.183695845
**CTC-523E23.5**	1.871293083
**CYP24A1**	2.791997977
**RP4-671O14.7**	1.553044079
**PXYLP1**	2.136978232
**TUT1**	1.234329147
**CTD-2331H12.7**	2.440253261
**KCNT1**	1.353292954
**RP11-347D21.1**	2.797278025
**TMEM107**	2.63795605
**RP11-502N13.2**	2.17837363
**CCDC188**	1.769428101
**RAPGEF3**	1.117241524
**RP11-122G18.11**	2.265575678
**ARL3**	1.957650629
**UICLM**	2.680549537
**MAF**	2.110575241
**RNF157**	1.362040414
**TPM4**	1.309279996
**CHD5**	1.970230537
**IFT22**	1.551443388
**LYST**	2.66239747
**RP11-359M6.1**	2.775028196
**DIP2C-AS1**	1.243091589
**RP11-64I5.2**	2.640694012
**CCDC121**	1.624106853
**ITGA3**	2.4449669
**ZBTB16**	2.363560858
**ARMC8**	1.873571794
**CHRNA6**	2.740732117
**SCUBE2**	2.613309103
**RP11-15A1.3**	1.848009958
**MFSD14B**	1.868247145
**LMX1A-AS1**	2.302174345
**DHRS3**	2.731969789
**SKP1**	1.441647622
**PDCL3P5**	2.6148881
**ZNF391**	1.331822596
**ATP9B**	2.683450567
**CTD-2126E3.5**	2.417566576
**ZNF385C**	1.687945698
**FBXO24**	1.69533208
**TTC39A**	1.280001182
**CTD-2639E6.11**	1.115836208
**IQCN**	2.41651691
**RP11-72O9.5**	2.737046343

**PXDN**	1.909320497
**ST8SIA6-AS1**	2.773235725
**EPS8L2**	2.044034925
**CTD-2516F10.2**	1.470010902
**RP11-98D3.2**	2.597397814
**RP4-534N18.2**	2.234882148
**FUBP1**	2.404698009
**TMEM86A**	2.729709099
**LINC02026**	2.698968134
**LSM4**	1.503855301
**NEIL2**	1.609873135
**C6orf223**	1.86056878
**GRK2**	1.37541334
**FLRT2**	1.721868896
**AC097374.2**	1.924687104
**CFLAR**	1.994029441
**PLB1**	2.702744785
**SSTR1**	2.607425241
**LINC02559**	2.631569355
**PGM2L1**	2.380109201
**GLS**	1.162514931
**RP11-475G3.1**	1.254148149
**COL1A2**	1.841500981
**RP11-54O15.3**	1.856393337
**RFX7**	2.562996168
**RAD21**	1.13908889
**TKT**	1.763533038
**U91328.21**	2.683747043
**RP1-80N2.2**	2.565269123
**RP11-154D6.1**	1.102855636
**RP11-798K3.2**	2.711149564
**FRG1CP**	2.242345133
**RP11-973N13.4**	1.11750352
**AC012078.2**	2.671209246
**CEPT1**	2.045341883
**ST3GAL5**	1.538664377
**PDCD6IP**	1.889461107
**SERPINE1**	2.675619235
**CROCC**	1.884703264
**GRHL2**	2.233541006
**REST**	1.20501567
**KAT8**	1.876013695
**RELT**	2.657529633
**CMTM2**	2.367336457
**RNPEP**	2.555818968
**RPL26P3**	2.682612171
**RP11-713M15.2**	2.71343179

**RP11-195F19.30**	1.942477735
**RP4-782D21.1**	2.644952826
**LINC02289**	2.561159041
**FAM43A**	2.120828947
**ZFP69**	1.194868912
**TTC14**	2.018718173
**ATG9B**	2.115170418
**PCOLCE**	2.059379378
**ITGB7**	2.421632831
**PAPSS2**	1.11376578
**EHD3**	1.548929015
**RAI14**	2.337593048
**RP11-564P9.1**	2.626150374
**MT-RNR1**	1.333893897
**CTD-2521M24.11**	2.643423125
**RP11-440I14.4**	2.515184121
**HDGFL3**	1.29543536
**PTCH2**	2.016308507
**RIPOR3**	2.131180365
**RTEL1-TNFRSF6B**	2.086740818
**KCNH1**	1.390466523
**MBNL2**	2.569772793
**RP11-313D6.3**	1.426374143
**LINC00565**	2.615155018
**RP11-71L14.3**	2.615155018
**SNHG16**	2.20762227
**AC097468.4**	2.609482468
**FZD6**	1.708543438
**RP11-932B15.2**	1.306313864
**RP11-290L1.2**	1.624099611
**NELFCD**	1.605455924
**ZNF747**	1.125612185
**RP11-123M6.2**	2.666608505
**STARD5**	1.333387493
**RP11-17J14.2**	1.336779871
**LINC02020**	2.643640386
**RP11-3L10.2**	2.423443909
**TMEM108**	1.505619217
**ACTA2**	1.569734196
**LINC00683**	2.089326663
**RPL9P2**	1.667958432
**RP11-107G24.4**	1.386076143
**CCDC80**	1.384810062
**RIF1**	1.796359274
**ZNF510**	2.472733438
**SLC16A6P1**	2.475734883
**RPL29**	1.698641087

**SLC30A4**	2.629300285
**TMC2**	1.226099763
**TCAF2**	2.51486773
**PCSK1**	2.194473554
**SSBP4**	1.210313909
**UTP25**	1.973062144
**ITCH**	1.622988148
**IER5L**	2.052201559
**MYO10**	1.443523687
**INPP5D**	2.569567732
**MTND6P22**	2.486039384
**C10orf55**	1.283470183
**CGB7**	2.300675147
**NATD1**	2.002571624
**PEX5L**	1.281273356
**RP13-487P22.1**	1.335041521
**MAP10**	1.854371585
**ADAMTS12**	1.994478918
**XKR6**	1.822900944
**ETS2**	2.604455611
**WDR87**	1.412835079
**ANO4**	2.563863322
**LANCL3**	2.017124144
**RP11-168F9.2**	2.24952201
**GS1-199J3.3**	2.495221469
**PRDX3P4**	1.702747351
**CTD-2081C10.8**	1.965705908
**LRRTM2**	1.851640468
**DMAC2**	1.950475232
**RP11-310N16.1**	2.574776862
**RP11-756J15.2**	2.560112702
**ZNF781**	2.460123128
**FOXJ3**	1.796846295
**NTF4**	2.248112978
**SPATS2L**	1.179405221
**IFT81**	2.100687852
**OPA3**	1.651600518
**BAALC**	1.727867708
**TAF3**	1.666673143
**RP11-66B24.4**	1.488372387
**GOLGA4**	1.29790262
**LRPPRC**	1.979559314
**LRRC9**	2.322796058
**CTB-109A12.1**	2.25321982
**BIRC5**	2.528786109
**MIR122HG**	2.069225731
**TSEN54**	1.865057685

**RUNDC3A**	1.471443678
**SNHG22**	2.260694979
**CTD-2515H24.4**	1.562378764
**TIFAB**	2.519380258
**ARSDP1**	2.509421788
**EVA1A-AS**	2.517959832
**FKBP14**	1.142605479
**RP11-582J16.7**	2.517959832
**TMEM199**	2.255413244
**ECHDC2**	1.47824672
**RP4-671G15.2**	2.197833301
**RPS21P4**	1.91376044
**HELZ-AS1**	2.437589473
**RP11-646I19.1**	2.568569578
**ADA**	1.325311105
**RP11-284F21.11**	1.42564194
**RPL27A**	1.183314532
**AC067945.4**	1.406828516
**PLA2G4C-AS1**	1.224272343
**RP11-792A8.4**	1.395325912
**EEA1**	2.42810185

Supplementary Table 2(b): Upregulated Genes in *PINK1* and *PRKN* mutant DA neurons. This table lists genes that exhibit significant upregulation (log2 Fold Change (FC) > 1.1 and False Discovery Rate (FDR) < 0.05) in *PINK1* and *PRKN* mutant DA neurons, compared to control neurons. Each gene is identified by its gene symbol, along with the log2FC value.

**Supplementary figure 2(c)**	
**Downregulated genes with log2FC<-1.1 and FDR<0.05 for homo. and hetero. PINK1 mutant DA neurons**	
**GeneId**	log2FC
**RP11-402D21.2**	-5.503060723
**AKAP13**	-4.458268188
**LRBA**	-4.35555648
**OFD1**	-4.660539109
**RP11-482D24.3**	-4.95625342
**RP11-6O2.4**	-2.522414018
**RP5-998C11.1**	-3.339605856
**XXyac-YX65C7_A.2**	-2.551264314
**TCP1**	-3.34661759
**NKX2-2-AS1**	-9.124538173
**CTD-2636A23.2**	-3.51666303
**LCORL**	-2.953766005
**ADCY3**	-2.182311088
**NR2F1-AS1**	-3.228269554
**CTC-463N11.4**	-2.195253979
**INPP5A**	-5.891596683
**CLCC1**	-2.631241305
**RP11-234G16.4**	-3.844382189

**RP11-482D24.2**	-4.517316889
**PRC1-AS1**	-2.541092427
**GRM3-AS1**	-3.018101625
**SOX21-AS1**	-4.920333546
**RP11-427I6.5**	-2.414391746
**ZEB2-AS1**	-4.41443269
**AP001469.5**	-2.808533458
**ZDHHC8**	-4.686868471
**LINC00621**	-4.181228446
**RP11-272L14.3**	-1.60808196
**MCF2L2**	-2.022392628
**RP11-435O5.5**	-4.135269746
**SEMA6A-AS1**	-2.237410323
**TPT1-AS1**	-2.693380841
**VIM-AS1**	-1.632958698
**RP11-572N21.1**	-6.617959609
**RP11-284F21.7**	-5.682406862
**C12orf65**	-1.809120857
**RGMB-AS1**	-3.322667495
**HADHA**	-3.258201297
**LINC01572**	-5.581974164
**FADS1**	-2.761686879
**RP11-124N14.3**	-1.557316697
**ITFG2**	-3.114762082
**DENND1A**	-2.055299124
**RP5-955M13.4**	-3.060008165
**RBIS**	-1.674696437
**IDH3A**	-3.334365935
**RP11-394B2.1**	-1.254197074
**AFDN-DT**	-4.498321932
**AC004538.3**	-3.216915993
**AATK**	-1.761298956
**ALG12**	-2.658225648
**CERT1**	-2.272834449
**GS1-72M22.1**	-2.399059501
**SEMA4D**	-2.094866516
**MARCKS**	-2.078979167
**GSTM3P1**	-5.331968168
**SLC25A26**	-1.831499337
**RP11-59C5.3**	-1.502646994
**RP11-74H8.1**	-1.779716635
**TMEM186**	-1.867617524
**TMEM51-AS1**	-2.111581705
**CTD-2215L10.1**	-2.844192762
**CELSR2**	-3.785193709
**LINC00404**	-6.146163793
**RP11-342D11.2**	-1.825991061

**RP11-422N16.3**	-3.081611709
**HHIP-AS1**	-6.618089015
**GS1-34D21.1**	-2.285520808
**TUBB1**	-2.019879815
**SCYL3**	-1.458904063
**RP11-732A19.6**	-2.151168391
**NCAM1-AS1**	-1.40001536
**POLG**	-2.847395843
**LINC00928**	-3.229754317
**SKP2**	-1.226981447
**RNF217-AS1**	-1.841288874
**IDI2-AS1**	-1.945436918
**NDUFB9**	-1.536232923
**FAM172A**	-4.410777409
**CFAP44**	-2.549627603
**AC009133.15**	-1.602745181
**RP11-192H23.5**	-1.780188455
**JAKMIP2-AS1**	-1.372137695
**AC008746.3**	-2.978084128
**SGPP2**	-1.825955452
**FGFBP3**	-3.847212163
**RP11-344B2.2**	-1.714834469
**DCAF1**	-1.394777104
**LRRC8C-DT**	-1.748301246
**IFT20**	-2.717208282
**DCHS1-AS1**	-1.471624536
**PXN**	-1.89944989
**TRIM59**	-1.811312705
**RP5-872K7.8**	-5.206767838
**SHLD2**	-1.612806019
**RP4-555D20.1**	-2.457751418
**A1BG-AS1**	-1.439995795
**ZNF710-AS1**	-1.364691393
**RP11-12J10.4**	-2.105544379
**AE000658.22**	-1.358493587
**KCNK12**	-1.823987397
**AC004160.4**	-3.430407431
**RP11-325J6.2**	-2.652944005
**RP11-296L22.8**	-1.642164812
**GSE1**	-1.900557823
**YIF1A**	-1.74438348
**FN1**	-1.543344684
**AC008067.2**	-1.988348649
**WDFY3-AS1**	-1.430863159
**RP11-547I7.1**	-5.32804817
**SPRN**	-1.186677058
**EXOC6**	-5.73355803

**TIGD7**	-1.358135058
**RP13-238F13.3**	-4.225953038
**CTD-3094K11.3**	-2.95917999
**RP11-567G24.3**	-2.343486491
**PSMC1**	-1.295093919
**RUSC1-AS1**	-1.44836138
**IPPK**	-1.438297371
**RP11-204E9.3**	-1.678528825
**RP5-991G20.1**	-1.477817691
**OIP5-AS1**	-2.264981091
**SPON1-AS1**	-5.735314374
**AC004490.1**	-1.495399626
**TNFRSF25**	-3.954952811
**ANKRD37**	-1.2225813
**KIF7**	-4.254118663
**RP11-284H19.1**	-2.42150937
**CTD-2510F5.4**	-3.796743683
**RP11-845C23.3**	-1.570558717
**SREBF2-AS1**	-1.294293144
**MGC32805**	-1.91967589
**AKAP9**	-2.009549958
**CTSB**	-2.188533257
**RP11-234G16.6**	-2.971113624
**ADCY7**	-1.115288035
**RP11-1055B8.8**	-2.195453112
**BASP1P1**	-5.389764999
**RP11-360L9.7**	-1.73988748
**RP1-18C9.3**	-1.231903181
**GATA3-AS1**	-4.259925736
**DLX1**	-5.397179119
**NR2F2**	-2.966096679
**RP11-342C2.2**	-2.935866803
**RP11-353N14.7**	-2.127216101
**AC009133.21**	-1.334203625
**CDR1**	-1.735945068
**RP1-18D14.7**	-5.284856966
**NR2F1**	-2.006759178
**RP4-541C22.5**	-2.296808887
**VPS35L**	-1.499077965
**FAM169A**	-1.18046208
**RP4-734G22.3**	-1.616976931
**RP11-226E21.4**	-1.15577993
**PC**	-1.103208289
**LRRC41**	-2.089041436
**CENPN-AS1**	-2.097597544
**GFAP**	-1.489939505
**WDFY3-AS2**	-1.768354762

**AC083884.8**	-1.157683109
**CASK-AS1**	-1.655476002
**RIOX2**	-1.176058741
**RP11-463O12.3**	-1.218505403
**RP11-149P14.2**	-1.701417094
**RP11-284F21.10**	-4.120854843
**AC053503.6**	-3.229657207
**MAP1B**	-1.264951477
**RP11-113H14.3**	-2.051874464
**ENSG10010137683.1**	-5.061159999
**RHCG**	-4.372430373
**CTD-2555O16.4**	-1.305653484
**UBXN6**	-1.531502929
**XXbac-B562F10.11**	-1.525603246
**HOTAIRM1**	-5.146298549
**RP3-326L13.2**	-4.959755324
**RP11-261N11.8**	-1.910298409
**RP11-544A12.4**	-1.466442173
**NUP153-AS1**	-1.456866449
**RP5-1024G6.5**	-1.170846026
**RP11-25K19.1**	-2.467493184
**RP11-945C19.4**	-1.500623417
**RP11-103J8.1**	-1.586354702
**FABP7**	-3.504006187
**CTC-250P22.2**	-1.709400265
**RP11-4N23.1**	-2.173367523
**ZNF276**	-1.204492213
**KLC1**	-1.314289261
**RP11-275G7.2**	-2.294569296
**RP11-95F22.1**	-1.724122082
**RP11-300E4.2**	-1.448702299
**INTS8**	-2.380700746
**RP11-830F9.5**	-1.708503919
**ZMYM4-AS1**	-1.610552107
**SPATA46**	-2.437624609
**RP11-34F13.2**	-3.51877649
**SMAP1**	-2.155489624
**AC007405.8**	-2.191296333
**RP11-379K22.3**	-1.215100401
**STRADA**	-1.38765132
**LHX5**	-3.401396151
**GRM5-AS1**	-3.024646028
**RP11-375N15.2**	-1.22024056
**NUP62**	-2.643714644
**RP11-435D7.3**	-2.924043112
**RP11-144F15.1**	-1.803133904
**NDUFA4L2**	-2.387813593

**ZEB2**	-2.82026811
**YPEL4**	-1.762930345
**KAAG1**	-2.229391962
**NPB**	-1.698536491
**RP11-626E13.1**	-1.695921208
**DELEC1**	-3.024116513
**RP11-815J21.2**	-1.32662472
**MARS1**	-2.134845221
**MEIS1-AS2**	-3.389715857
**RP11-5O23.2**	-1.31034761
**ERBIN**	-3.652247382
**ENSG10010137930.1**	-4.707305644
**UROD**	-2.028988431
**AF196970.3**	-1.761628676
**DNAJC11**	-1.340736503
**NES**	-2.519464857
**PLEKHJ1**	-1.440885633
**METAP1D**	-4.49414034
**HEPN1**	-2.465217641
**RP11-860B13.3**	-1.128286426
**AC022007.5**	-1.202242898
**KIAA1614**	-1.246812674
**AC005943.6**	-1.411512744
**AC009480.3**	-1.876067341
**C9orf16**	-1.258518097
**RP1-261G23.7**	-1.13162442
**TSSK2**	-1.498008865
**AC012363.4**	-1.240254879
**RP11-85O21.5**	-3.072425553
**PTN**	-3.14011867
**TLCD3A**	-1.37396539
**RP11-196H14.2**	-2.135850072
**SGO1-AS1**	-3.278470618
**RP4-694A7.2**	-3.463458649
**CCDC103**	-3.357756376
**LIMS2**	-3.554554964
**RP11-33O4.3**	-1.546817991
**VIM2P**	-1.920871291
**RP1-69D17.4**	-1.954945886
**RP11-265N6.2**	-1.127857589
**RP4-671O14.5**	-2.092372541
**MPEG1**	-2.675345142
**TRAPPC2**	-1.484998502
**URM1**	-3.833608335
**SLC32A1**	-4.089633998
**KATNAL2**	-1.668089925
**FANCD2OS**	-2.106988877

**TMPO-AS1**	-1.555568199
**RP11-481J2.3**	-2.395347834
**NT5DC4**	-3.579804739
**FADS2**	-1.802028471
**TACC3**	-1.6688274
**AC005481.5**	-3.463002365
**RP11-780K2.1**	-1.600036512
**EXOSC9**	-1.556978108
**RP3-514A23.4**	-4.334821249
**POU3F3**	-3.262896361
**ITGB8-AS1**	-2.960288691
**TUBAP12**	-4.055888662
**CTD-2619J13.17**	-1.626398691
**SMIM4**	-1.412910704
**CHRM5**	-2.763399869
**NADK2-AS1**	-1.69527959
**RP11-536C5.7**	-1.363608737
**VIM**	-1.210533919
**PAQR4**	-2.241667052
**ZNHIT2**	-1.914655703
**RP11-49K24.3**	-3.683455136
**RP11-4N23.4**	-1.418192546
**CNTFR-AS1**	-1.309682503
**LCOR**	-3.091395654
**RP11-573D15.8**	-1.529409778
**LINC00867**	-1.320183119
**MYCL-AS1**	-1.742328541
**TRIM37**	-1.308282245
**MYO1E**	-2.700250688
**RP4-784A16.2**	-1.738061625
**EDNRB**	-3.973335531
**LINC02487**	-3.205821613
**RP11-755H23.1**	-1.880678962
**RP11-154I21.1**	-3.614527751
**PIEZO1**	-1.429892104
**CTD-2015C24.1**	-3.899688316
**GLB1L2**	-1.30881951
**SPC24**	-2.920083861
**AJM1**	-1.971330088
**MN1**	-2.248210913
**WDR24**	-1.292226972
**RNF2P1**	-1.986922291
**ZNF862**	-1.24756872
**RP5-896L10.1**	-2.21739316
**RP11-85O21.2**	-4.210831613
**TMEM213**	-1.195460954
**HAS2-AS1**	-2.205028486

**RP11-16C18.3**	-1.191944754
**ATXN1L**	-1.273598126
**DDX20**	-1.39871083
**PAH**	-1.988973338
**LINC01917**	-1.376848893
**LYPD1**	-1.677880026
**RP5-994D16.9**	-1.280592023
**ACADVL**	-1.336730213
**CTD-2555A7.2**	-4.0653698
**HCCAT5**	-3.035648777
**MARK2P16**	-2.076887121
**RP11-989F5.1**	-1.87363307
**NHLH2**	-2.345383402
**LRRC7-AS1**	-1.886866064
**RP11-146F11.1**	-1.717507232
**SHH**	-2.912041329
**LINC01361**	-4.010799143
**UCHL5**	-1.528537805
**SNRPGP10**	-2.266678678
**RP11-435O5.4**	-2.191019789
**RP11-73E17.2**	-2.266542479
**RP11-379F12.4**	-2.436629195
**RP11-1000B6.9**	-3.736003295
**RP11-95O2.5**	-1.184456797
**TMED1**	-1.105870756
**CTD-2353F22.1**	-2.593187559
**AC022154.7**	-1.285541729
**RP11-380I10.4**	-3.973558565
**ERRFI1**	-2.500284544
**DNHD1**	-1.302969192
**EHBP1**	-2.731462084
**SIGLEC10**	-2.991418707
**MKRN3**	-1.979961354
**RP11-30L15.4**	-1.925226336
**EFCC1**	-3.395160759
**RP11-95J9.3**	-1.228371929
**GAS2**	-1.127477031
**RP11-80B9.1**	-1.300289879
**AC037445.1**	-1.815294179
**AC079135.1**	-3.680160511
**RP11-1140I5.2**	-1.876495094
**RP11-739B23.1**	-1.29164091
**AC068057.1**	-3.157239049
**RP5-966M1.7**	-1.43112056
**C11orf42**	-1.936472705
**TMEM161B-AS1**	-2.50266361
**CTD-2619J13.9**	-1.465617349

**RAET1G**	-3.189138788
**SKIDA1**	-1.421722414
**PCA3**	-1.450825162
**AF131216.7**	-1.269797789
**SPDYE6**	-2.476656676
**BAHCC1**	-2.628024325
**ASB8**	-1.189399922
**PKM**	-1.13880643
**RP11-110I1.6**	-1.109677966
**DIS3**	-1.178085926
**CTC-563A5.5**	-1.152175239
**BRAT1**	-1.277441278
**RP11-552F3.9**	-1.818956003
**DAAM2-AS1**	-1.639566545
**LINC00200**	-1.630664892
**GAD2**	-3.524905599
**AC000095.9**	-1.164944825
**INHBE**	-2.692237963
**RP11-395N17.3**	-2.15111963
**LINC01798**	-3.106200782
**NOXO1**	-1.402605131
**CTD-2282P23.2**	-3.533374117
**QKI**	-2.029118073
**SPATA7**	-1.233256496
**ZFPM1**	-1.893256989
**PSMB7**	-1.517703797
**JAKMIP2**	-2.864606113
**TECR**	-2.086771625
**SYN2**	-2.968806132
**RP11-483P21.2**	-1.170138445
**ACMSD**	-1.468069395
**POU3F4**	-3.724009066
**RP11-546B8.6**	-3.820481263
**RASSF10**	-2.632046382
**MYT1L-AS1**	-2.207593065
**PAX9**	-2.133354792
**NDP-AS1**	-2.373631536
**FOXD1-AS1**	-2.754029427
**RP11-57H14.2**	-1.607382557
**RP11-269F19.10**	-1.658241571
**BNIP3P17**	-1.866595248
**RP11-229P13.23**	-1.531626788
**TFAP2A-AS2**	-3.681856349
**RP11-766F14.1**	-3.649486825
**LINC00237**	-3.645701006
**TTC23**	-1.947748366
**CTD-2647L4.1**	-1.348251286

**AC007563.5**	-2.428501272
**PTBP1**	-1.38015385
**RNA5SP216**	-1.145117515
**DHRS7B**	-1.631681279
**LINC00944**	-3.698491914
**LINC02157**	-2.409450393
**TSTD2**	-3.045958285
**NTRK2**	-2.986710505
**LBX1**	-3.616338568
**RP3-514A23.2**	-3.616344893
**CCDC107**	-1.835817316
**RP11-315D16.4**	-1.406358044
**RP11-135A24.4**	-1.64884676
**SLC9A7**	-1.333034466
**RP11-223P11.3**	-1.103545579
**RP11-981G7.3**	-1.82349215
**PDK3**	-2.454490205
**SOX2**	-2.413719105
**AC007326.9**	-1.878197728
**CCP110**	-2.016286578
**RP11-33N14.3**	-1.142060935
**PTPRN**	-1.675837908
**RP11-397O8.7**	-2.009169644
**RP11-219B4.3**	-2.164305635
**ADGRL1**	-1.128601489
**KDM8**	-1.825142166
**KLHL34**	-1.806888158
**VAC14-AS1**	-1.194057959
**RP5-1055C14.9**	-2.473130895
**SOX11**	-1.898834487
**RP11-549L6.3**	-1.82305563
**NTN1**	-1.736717102
**PPP1R12B**	-1.466350314
**LUARIS**	-1.616832618
**TOX**	-2.470280092
**AC093390.1**	-2.347523499
**RP11-662I13.3**	-2.513964411
**NOTCH1**	-2.73794731
**GRPEL1**	-1.309639528
**MID1IP1-AS1**	-1.555006496
**RP11-446J8.1**	-1.649394583
**RP11-513M16.8**	-1.15033421
**ZBTB20-AS1**	-1.932299441
**ADAM1B**	-1.431626752
**ITGAE**	-2.817190249
**MYCNOS**	-1.336627572
**PLSCR5-AS1**	-3.097707828

**SOX8**	-3.214446871
**LINC00632**	-3.395568622
**PRORP**	-1.69775987
**RP11-346M10.3**	-2.795995922
**RP11-379K22.2**	-1.94020322
**OR52W1**	-3.374768377
**LAMP5-AS1**	-3.370451314
**PPFIA2-AS1**	-1.276500403
**MROCKI**	-2.15633742
**SLC7A11-AS1**	-1.548389769
**CTD-2555O16.2**	-1.452226081
**RP11-424N24.2**	-1.871862126
**DCDC2**	-2.513364193
**RMC1**	-1.117639484
**SBK1**	-1.557799504
**SOX1**	-3.17493389
**GRM7-AS3**	-3.318777802
**UNC5C**	-2.073461056
**PTENP1**	-1.82668347
**RECQL4**	-1.609337157
**NDUFC1**	-1.15970244
**C9orf147**	-1.807064831
**MIR3665**	-2.786844663
**RP11-400K9.3**	-2.790898206
**MST1**	-1.514276315
**RP3-512E2.2**	-1.305863748
**MAST2**	-1.874431595
**RP11-436D10.3**	-1.103071984
**LA16c-395F10.1**	-1.217983601
**TUBB2B**	-1.183235174
**CEP57**	-1.254772701
**SMAD1**	-1.593833164
**FAM86B1**	-1.546339343
**CTC-563A5.2**	-1.288177819
**BACH1**	-1.833010777
**PRPF40A**	-1.283965129
**CTA-38K21.6**	-1.65812049
**RP11-325L7.1**	-1.179268581
**RP11-45M22.3**	-1.258064272
**NRXN2-AS1**	-1.988809638
**RP1-267L14.6**	-1.147317523
**BTBD6**	-2.552072659
**LUCAT1**	-1.101238736
**LRP11**	-2.597145157
**MED14OS**	-1.536323322
**RP11-499P20.2**	-1.378041132
**FEN1**	-1.227843715

**NCOR2**	-1.189885364
**RP11-625L16.3**	-3.187965664
**SNAI3**	-1.499885193
**STAC3**	-1.74052391
**DUX4L50**	-1.55717758
**RP11-420L9.5**	-1.112737046
**RP11-507J18.2**	-1.782697194
**NAA25**	-2.862985538
**SATB2-AS1**	-2.497018587
**HOXA-AS3**	-3.322889309
**DCHS2**	-1.840422552
**ONECUT1**	-3.154994754
**BMP2K**	-1.328274057
**MIR762**	-2.243885969
**SMCR8**	-1.424688612
**KLB**	-1.811791586
**ZNF285B**	-1.366714281
**R3HDM2**	-1.840286705
**CD44-AS1**	-1.913569041
**SEMA6A**	-1.189194915
**PTPRZ1**	-2.94613481
**SSPN**	-1.286284328
**RP1-104O17.3**	-3.125531289
**RPS20P4**	-2.226881954
**RP11-128A17.1**	-1.910268251
**UFL1-AS1**	-2.749884712
**RP11-234O6.2**	-1.744609083
**RP5-1103B4.3**	-1.112566977
**GALR1**	-2.718980866
**RP11-431N8.1**	-1.256442393
**MIR4500HG**	-1.359963458
**IRS1**	-1.323570701
**MIR9-3HG**	-2.229121925
**RP11-394B2.5**	-1.174432214
**PRSS51**	-1.332789575
**PRRT3**	-1.347282774
**NKX2-2**	-3.041617821
**ZSWIM7**	-1.461865637
**AC000032.2**	-2.014260605
**ANAPC11**	-1.758302343
**LEF1-AS1**	-1.866955807
**HMMR-AS1**	-2.283187681
**CTD-2002J20.1**	-1.706367801
**ANXA2P1**	-2.380009977
**DHDDS**	-1.278149317
**RP3-395C13.2**	-1.618723783
**USP17L2**	-2.880446981

**RP11-118M9.3**	-2.204136807
**RP5-1029K10.2**	-1.817658416
**SOX1-OT**	-3.027101849
**CCDC134**	-1.28977044
**RNF139-AS1**	-1.531043861
**ACTR3C**	-2.633428156
**UBAP1**	-1.50162601
**CTB-50E14.4**	-2.085638399
**HMGB3P24**	-1.671880474
**RP11-21C4.1**	-3.036450027
**RP1-69D17.3**	-1.429544271
**CTB-187M2.2**	-1.111906274
**AGGF1**	-1.952341907
**SBNO1-AS1**	-2.028416912
**RP11-234G16.5**	-2.628161524
**CAMK4**	-1.394737083
**CYC1**	-1.169446313
**USP1**	-1.807140447
**SLIT1**	-2.216779725
**HSPH1**	-1.310282093
**ARL8B**	-2.59046196
**ITGA9-AS1**	-1.599929876
**ACAD9**	-1.201226593
**RP11-51B6.1**	-2.905642698
**KIRREL3**	-2.092721997
**NRL**	-1.864274492
**SRPX2**	-1.713571996
**SOX9**	-2.198387527
**SCD**	-2.024748719
**TXNL1**	-1.467153519
**WARS1**	-1.985744806
**RP11-1084A12.2**	-1.105054777
**RN7SKP275**	-2.339347634
**PRMT6**	-1.178257191
**CACNA2D3-AS1**	-1.726072177
**HOXB3**	-2.671644715
**PREX1**	-2.540105008
**GRIK4**	-2.856642311
**LRP4-AS1**	-1.260894998
**WI2-85898F10.2**	-2.360434445
**DLX6**	-2.866489168
**SCRG1**	-1.775317428
**CTD-2349P21.11**	-1.691606322
**RP11-435O5.6**	-2.838406827
**TSPAN11**	-1.24239516
**ALKBH3-AS1**	-1.422329447
**IGBP1P1**	-2.376469438

**CAB39**	-1.399256824
**LINC01159**	-2.147336744
**NPAS3**	-2.24225906
**ZNF597**	-1.197277155
**EVC**	-1.486842455
**RP11-235E17.6**	-1.649446809
**RP11-304L19.4**	-1.529033002
**EDC3**	-1.313418964
**INTU**	-2.465763926
**RP11-303E16.10**	-1.292818542
**PRIMPOL**	-1.556723703
**AC090616.2**	-1.203050122
**RP11-71H17.1**	-2.775783304
**ENSG10010136622.1**	-1.344963063
**PNPLA3**	-2.680382252
**LDLR**	-1.485902011
**YIPF3**	-1.624301464
**C12orf45**	-1.448612501
**RP11-644F5.11**	-1.609948702
**LINC01668**	-2.34117529
**RP11-482M8.1**	-2.323581227
**ACBD6**	-2.58306301
**RP11-389G6.4**	-1.514335964
**AUTS2**	-1.175458952
**MKI67**	-2.364478914
**SHC3**	-1.942674283
**RP13-895J2.3**	-1.868248959
**PAX8-AS1**	-2.124634059
**AC068196.1**	-1.318037937
**CICP16**	-1.309073614
**SPDYE1**	-1.721264323
**RP5-872K7.7**	-2.419309112
**SGCZ**	-2.21153075
**TNC**	-2.057433302
**RSRC1**	-1.621467101
**RP11-411B10.3**	-1.246108364
**POU3F2**	-1.754763414
**RP5-921G16.1**	-2.645788464
**GRPEL2-AS1**	-1.199265367
**RP11-318N11.1**	-1.934353692
**SQLE**	-2.161463236
**DRG1**	-1.769377541
**ZMIZ1-AS1**	-1.745086962
**SYCE2**	-1.193359873
**ITSN1**	-1.122752834
**UBA1**	-2.057949065
**SLC35G6**	-1.674412776

**MPRIPP1**	-1.159329808
**STXBP3**	-2.591509129
**AKR1C1**	-2.125703014
**RP11-754H12.1**	-1.771000382
**AC055764.1**	-1.174908161
**FGF13-AS1**	-1.597226605
**AFDN**	-1.227792871
**MMP15**	-2.290241283
**RPL22P24**	-1.130790544
**LRP2**	-2.652155607
**OARD1**	-1.128717339
**RP11-802D6.1**	-1.575827344
**LINC02006**	-2.328518459
**RP11-420K8.1**	-1.209841121
**C12orf43**	-1.783001317
**RP11-50D16.4**	-2.164709345
**RP11-19B4.2**	-1.846313412
**ABCA3**	-2.119525172
**HIP1R**	-1.221585162
**GATA3**	-2.634399988
**GPR182**	-1.790677
**ZNF516-AS1**	-1.439073909
**KREMEN2**	-1.168614998
**ATP2C2-AS1**	-1.749323967
**SPATA42**	-2.665003896
**CSNK1G3**	-1.502602776
**PDHA1**	-1.279295735
**SNTG1**	-2.011133421
**CTD-2517M22.17**	-1.504590298
**RP11-677M14.7**	-1.875974309
**FOXD3-AS1**	-2.608226859
**ASIC3**	-1.318179471
**NFIX**	-2.603194297
**GNAS-AS1**	-1.762233869
**PIGZ**	-1.5837289
**DPP7**	-1.124048606
**RTL5**	-1.351241685
**CTD-2335A18.2**	-2.617056303
**STEAP3**	-1.260951859
**CAHM**	-2.474162492
**INSYN1**	-2.473061699
**PCDHB15**	-2.469823882
**HSD17B6**	-1.231410056
**RP11-128P10.1**	-1.298916029
**RP11-1330D14.1**	-2.122686387
**PGBD4P3**	-1.652314855
**ZSWIM5**	-2.062497786

**DOK7**	-1.510150704
**RP4-760C5.3**	-2.321671647
**RP11-401P9.6**	-1.707130455
**RP11-478H11.3-001**	-1.921790826
**CDC23**	-2.299360411
**RP11-45A17.2**	-1.43017403
**AC104131.1**	-2.604551193
**Z82214.2**	-1.411970261

Supplementary Table 2(c): Downregulated Genes in *PINK1* and *PRKN* mutant DA neurons. This table lists genes that exhibit significant downregulation (log2 Fold Change (FC) > 1.1 and False Discovery Rate (FDR) < 0.05) in *PINK1* and *PRKN* mutant DA neurons, compared to control neurons. Each gene is identified by its gene symbol, along with the log2FC value.

**Supplementary table 3(a)**	
**Gene Id**	log2FC
**EXOC6**	-5.405677811
**RP11-482D24.3**	-5.367352939
**ENSG10010137930.1**	-5.248384302
**RP11-547I7.1**	-5.166396542
**ENSG10010137683.1**	-4.967944006
**OFD1**	-4.932170129
**ZDHHC8**	-4.81656645
**RP11-482D24.2**	-4.788751405
**HOXA10-AS**	-4.737610219
**FAM172A**	-4.667594178
**RP11-284F21.10**	-4.661955343
**SOX21-AS1**	-4.53269563
**RP5-872K7.8**	-4.47535043
**LINC00621**	-4.470249015
**HOXB3**	-4.456810712
**LRBA**	-4.448099466
**AC005481.5**	-4.444310576
**AKAP13**	-4.384201174
**ACBD6**	-4.383208303
**LINC00944**	-4.377312506

**MEIS1-AS2**	-4.36367608
**RP1-18D14.7**	-4.307743908
**LIMS2**	-4.276989966
**ZEB2-AS1**	-4.206327087
**RP11-435O5.5**	-4.149354867
**GATA3-AS1**	-4.113744612
**RP3-514A23.4**	-4.104586363
**RHCG**	-4.092463152
**KIF7**	-4.079503323
**RP11-380I10.4**	-4.069238513
**SLC32A1**	-3.973862774
**HOXA2**	-3.936908545
**DELEC1**	-3.910349581
**POU3F4**	-3.884091678
**URM1**	-3.865603573
**LINC01361**	-3.865501862
**TSTD2**	-3.859747855
**AFDN-DT**	-3.844297846
**HOXA10**	-3.784836866
**HADHA**	-3.737598232
**LINC01798**	-3.715473375
**RP11-1000B6.9**	-3.631283444
**CELSR2**	-3.60533905
**LCOR**	-3.58793148
**CTD-2510F5.4**	-3.587037687
**GALR1**	-3.582803918
**CTD-2015C24.1**	-3.519658418
**RP5-1055C14.9**	-3.492764621

**NT5DC4**	-3.492600461
**FGFBP3**	-3.492158813
**SYN2**	-3.47484052
**RP13-238F13.3**	-3.46448606
**RP4-694A7.2**	-3.451959167
**LINC00928**	-3.438777125
**AC007563.5**	-3.437027425
**RP11-234G16.4**	-3.424187602
**ERBIN**	-3.361193628
**LHX5**	-3.356075821
**USP17L2**	-3.336023484
**RP11-422N16.3**	-3.302803594
**RP5-998C11.1**	-3.249298511
**IDH3A**	-3.243871753
**HAGLR**	-3.235353262
**AC004160.4**	-3.16883494
**FOXD1-AS1**	-3.163236244
**SGO1-AS1**	-3.153066636
**TNFRSF25**	-3.138905842
**CCDC103**	-3.117872692
**CTD-3094K11.3**	-3.102041908
**RP11-49K24.3**	-3.045958054
**GRM5-AS1**	-3.040868375
**CTD-2636A23.2**	-3.008085421
**ITFG2**	-3.00008304
**RP11-154I21.1**	-2.982308184
**AC053503.6**	-2.964477215
**LCORL**	-2.957792165

**TPT1-AS1**	-2.891596238
**ZEB2**	-2.880904446
**EFCC1**	-2.868937274
**RP11-435D7.3**	-2.865945028
**HEPN1**	-2.861179174
**AC004538.3**	-2.844629779
**TCP1**	-2.843503936
**GS1-72M22.1**	-2.831304593
**GRM3-AS1**	-2.818479734
**PAX8-AS1**	-2.814002022
**HOXB-AS2**	-2.811603443
**HOXA-AS2**	-2.811335911
**SHH**	-2.808489808
**FABP7**	-2.802614056
**DCDC2**	-2.799484904
**POLG**	-2.78756239
**RGMB-AS1**	-2.787089215
**NTRK2**	-2.775797652
**ITGB8-AS1**	-2.766493329
**NDP-AS1**	-2.765199889
**NR2F1-AS1**	-2.763152457
**TMEM161B-AS1**	-2.748374894
**RP11-128A17.1**	-2.744780231
**ALDH3A2**	-2.730970418
**AC008746.3**	-2.701855604
**MYO1E**	-2.695526131
**ALG12**	-2.69499968

**RP11-318N11.1**	-2.690228776
**ITGAL**	-2.687425281
**XXyac-YX65C7_A.2**	-2.685200994
**RP11-6O2.4**	-2.681412094
**MEIS1**	-2.680962078
**RP11-397O8.7**	-2.676086712
**HOXB-AS1**	-2.634142482
**SRPX2**	-2.633934072
**RP5-955M13.4**	-2.63093198
**KAAG1**	-2.626448693
**GNAS-AS1**	-2.61715628
**AC068057.1**	-2.614221252
**BAHCC1**	-2.590416791
**CLCC1**	-2.58891711
**INHBE**	-2.571038095
**PRC1-AS1**	-2.555298914
**CTD-2215L10.1**	-2.553744081
**CHRM5**	-2.553374003
**AP001469.5**	-2.546906994
**ATP2C2-AS1**	-2.546278414
**ERRFI1**	-2.515778119
**KLB**	-2.509337439
**RP11-234G16.6**	-2.49254543
**TNC**	-2.481627596
**RP11-342C2.2**	-2.481241795
**INTS8**	-2.477342187
**SNRPGP10**	-2.473217088
**RP4-555D20.1**	-2.465348952

**RP11-379F12.4**	-2.454432233
**RP4-671O14.5**	-2.448293563
**RP11-34F13.2**	-2.444457479
**NR2F2**	-2.412018329
**SMAP1**	-2.401022957
**DCHS2**	-2.38783771
**POU3F3**	-2.379342656
**RP11-25K19.1**	-2.348399576
**CD44-AS1**	-2.328554833
**HAS2-AS1**	-2.321843434
**MPEG1**	-2.318784777
**SHC3**	-2.295326501
**AC093390.1**	-2.285503444
**RP11-481J2.3**	-2.276651568
**FGF13-AS1**	-2.268449924
**RP11-73E17.2**	-2.25845021
**AGGF1**	-2.247423565
**LINC00511**	-2.24419447
**FADS1**	-2.243273682
**RP11-275G7.2**	-2.240574476
**RP11-567G24.3**	-2.230205256
**RP11-427I6.5**	-2.226937484
**TUBB1**	-2.217759433
**CDR1**	-2.207470185
**DENND1A**	-2.207321747
**DAAM2-AS1**	-2.203858398
**NDUFA4L2**	-2.196034735
**SEMA4D**	-2.192404654

**CCP110**	-2.190661968
**RP11-552F3.9**	-2.182009615
**TTC23**	-2.170338299
**FGF13**	-2.166618132
**CACNA2D3-AS1**	-2.159830106
**RP11-290O12.2**	-2.159710777
**PER3**	-2.15208
**TRIM59**	-2.149556662
**OIP5-AS1**	-2.146956083
**ADCY3**	-2.142214922
**MCF2L2**	-2.140972603
**RP11-353N14.7**	-2.125339567
**TECR**	-2.121119584
**NES**	-2.11547064
**ZBTB20-AS1**	-2.109206998
**RP11-1055B8.8**	-2.105608641
**RP11-342D11.2**	-2.097240455
**ZFPM1**	-2.097237153
**TMEM186**	-2.091871119
**RP11-12J10.4**	-2.08921809
**GS1-34D21.1**	-2.087204473
**NRXN2-AS1**	-2.086311431
**KLHL34**	-2.084804521
**NCOA3**	-2.074279078
**DUX4L50**	-2.069508311
**YIF1A**	-2.063822847
**RP11-732A19.6**	-2.063333641
**MGC32805**	-2.060389639

**RP11-284H19.1**	-2.056241377
**CTD-2353F22.1**	-2.05610727
**SEMA6A-AS1**	-2.029407728
**C12orf65**	-2.027357786
**CASK-AS1**	-2.013473357
**RP11-4N23.1**	-2.012276066
**RSRC1**	-2.010808952
**RP11-325J6.2**	-2.00895762
**LINC00200**	-2.004225397
**PPP1R12B**	-1.994164249
**RP11-74H8.1**	-1.983357744
**RP11-989F5.1**	-1.980946108
**NTN1**	-1.980533609
**RECQL4**	-1.979913214
**CENPN-AS1**	-1.977706346
**MID1IP1-AS1**	-1.967032922
**PAH**	-1.957575006
**UROD**	-1.944929105
**CTC-463N11.4**	-1.944188952
**RP5-896L10.1**	-1.939755313
**PIGZ**	-1.932125548
**IFT20**	-1.930138735
**MARCKS**	-1.927761164
**CERT1**	-1.923998984
**AC008067.2**	-1.917443162
**SYTL4**	-1.916865439
**SLC25A26**	-1.915555777

**DDX20**	-1.914403415
**AC007405.8**	-1.913536738
**FANCD2OS**	-1.91236296
**RP11-435O5.4**	-1.911262701
**GDE1**	-1.898249648
**TMEM51-AS1**	-1.887727563
**SPATA46**	-1.884228722
**RP11-344B2.2**	-1.883716523
**CEP57**	-1.88251036
**QKI**	-1.873659524
**AC007326.9**	-1.872458471
**TACC3**	-1.869742425
**NADK2-AS1**	-1.863998027
**RP11-360L9.7**	-1.86177615
**PAQR4**	-1.856890005
**LRRC41**	-1.856288747
**AKAP9**	-1.850491502
**CFAP44**	-1.848558667
**LRRC7-AS1**	-1.847206664
**WDFY3-AS2**	-1.834772042
**C11orf42**	-1.825892433
**RP11-261N11.8**	-1.823527648
**RP11-196H14.2**	-1.821015205
**NUP62**	-1.810415462
**RP11-1140I5.2**	-1.799626862
**KCNK12**	-1.796774402
**RP11-144F15.1**	-1.793759229
**AC037445.1**	-1.78668077

**EXOSC9**	-1.785808311
**Z82214.2**	-1.784634992
**PRORP**	-1.781794512
**RP11-146F11.1**	-1.776484324
**RNF2P1**	-1.773281463
**TSIX**	-1.772727856
**MARS1**	-1.76503967
**RP11-113H14.3**	-1.761487488
**RP11-626E13.1**	-1.75857086
**RP11-9N12.2**	-1.757342337
**PXN**	-1.751678669
**VIM-AS1**	-1.743751247
**PSMB7**	-1.741414653
**RP11-862L9.3**	-1.735828003
**TRAPPC2**	-1.733061626
**NPAS2-AS1**	-1.731763503
**AATK**	-1.725398037
**AC009480.3**	-1.721713207
**RP11-272L14.3**	-1.720218372
**SGPP2**	-1.716989441
**RP11-124N14.3**	-1.716560911
**AP1S2**	-1.703997389
**RP4-541C22.5**	-1.701593302
**CAB39**	-1.69444934
**RP11-5O23.2**	-1.678151434
**CTC-250P22.2**	-1.665079862
**RP11-315D16.4**	-1.653531577
**RP11-95F22.1**	-1.64507492

**FAM86B1**	-1.643548397
**CTD-2653D5.1**	-1.642446441
**PTPRN**	-1.633004345
**RP11-192H23.5**	-1.631211507
**MST1**	-1.630447071
**IGFBP5**	-1.620998284
**SKIDA1**	-1.619459095
**ASPM**	-1.618738099
**NR2F1**	-1.608404406
**CTA-363E6.6**	-1.603788501
**CTD-2555O16.2**	-1.600662634
**RP11-296L22.8**	-1.58741101
**ITGBL1**	-1.58321056
**ACMSD**	-1.576895224
**CTSB**	-1.57385692
**LYPD1**	-1.569996638
**GSE1**	-1.568252021
**UCHL5**	-1.559158778
**RP4-784A16.2**	-1.554964156
**RNF217-AS1**	-1.553566257
**KATNAL2**	-1.55120219
**AC009133.15**	-1.550733296
**RP11-573D15.8**	-1.55024837
**IDI2-AS1**	-1.545901822
**YPEL4**	-1.543473289
**DHX38**	-1.542257871
**IPPK**	-1.531977424
**SCYL3**	-1.523823614
**PCA3**	-1.520343006

**A1BG-AS1**	-1.51989502
**VPS35L**	-1.516810967
**RP11-755H23.1**	-1.514184137
**RP11-945C19.4**	-1.511123906
**RP11-103J8.1**	-1.510963479
**FN1**	-1.505857367
**RP11-780K2.1**	-1.50316886
**TRIM37**	-1.499339593
**RP11-149P14.2**	-1.499148109
**ZMYM4-AS1**	-1.498777665
**SHLD2**	-1.498166137
**RBIS**	-1.494806969
**NDUFB9**	-1.494171269
**CTD-2619J13.9**	-1.493687218
**WDFY3-AS1**	-1.490616093
**RP11-536C5.7**	-1.486316703
**RP11-33O4.3**	-1.481858329
**AC016738.4**	-1.477671711
**CNTFR-AS1**	-1.466194436
**SCN1A-AS1**	-1.4594642
**RP5-966M1.7**	-1.456741341
**FAM198B-AS1**	-1.4566521
**AF196970.3**	-1.454603555
**WWTR1-AS1**	-1.449905388
**MYCL-AS1**	-1.447398922
**SLC9A7**	-1.446262262
**UBXN6**	-1.445558016
**FADS2**	-1.441221591

**RP3-512E2.2**	-1.441064537
**LRRC8C-DT**	-1.43612079
**AF131216.7**	-1.430898934
**XXbac-B562F10.11**	-1.429010678
**RP11-229P13.23**	-1.415318256
**NCAM1-AS1**	-1.414294024
**RP11-59C5.3**	-1.407149532
**ATXN1L**	-1.405164118
**AC022154.7**	-1.398143853
**LUCAT1**	-1.393360068
**RP11-204E9.3**	-1.391901128
**RP11-815J21.2**	-1.390725067
**GAD1**	-1.390658084
**LA16c-395F10.1**	-1.388114144
**BRAT1**	-1.387529924
**AC012363.4**	-1.387381653
**TMPO-AS1**	-1.384004953
**AC004490.1**	-1.382292268
**NUP153-AS1**	-1.377157695
**VIM2P**	-1.376823364
**TPSG1**	-1.37536817
**SUPT20H**	-1.374913102
**RP5-991G20.1**	-1.362081739
**PLEKHJ1**	-1.360688028
**RP11-467K18.2**	-1.357924817
**RP11-420L9.5**	-1.354828477
**RP11-394B2.1**	-1.35368258
**RAP2C-AS1**	-1.35196321
**TTN**	-1.34943287

**JAKMIP2-AS1**	-1.346522148
**TLCD3A**	-1.334625511
**RP11-830F9.5**	-1.333434094
**ZNHIT2**	-1.332954272
**RP11-16C18.3**	-1.331012413
**DDB2**	-1.324740599
**ENTPD1-AS1**	-1.315211246
**RP11-95O2.5**	-1.312487328
**AC009133.21**	-1.311384419
**C9orf16**	-1.3099921
**GPR39**	-1.309744726
**RP11-845C23.3**	-1.308856081
**RP5-1103B4.3**	-1.308071776
**CEP250-AS1**	-1.306884911
**CTD-3032J10.2**	-1.303188754
**VIM**	-1.300610331
**DCHS1-AS1**	-1.295031929
**RP11-4N23.4**	-1.294340739
**RP11-558B7.1**	-1.285723657
**ZNF862**	-1.28154806
**CTB-187M2.2**	-1.276706919
**RP11-234O6.2**	-1.275422803
**RP11-463O12.3**	-1.273017068
**GLB1L2**	-1.27183381
**ZNF276**	-1.26811891
**RP11-300E4.2**	-1.265479056
**THBS2**	-1.265036881
**RP11-226E21.4**	-1.262253053

**SKP2**	-1.260296258
**DNAJC11**	-1.259481045
**RP11-544A12.4**	-1.255558463
**RP11-379K22.3**	-1.254393831
**FAM169A**	-1.253637739
**NCAPD3**	-1.24737902
**MCPH1-AS1**	-1.247097425
**RP11-513M16.8**	-1.247007866
**SPRN**	-1.243754524
**RP4-678D15.1**	-1.243350753
**ZNF710-AS1**	-1.236762863
**TIGD7**	-1.230742559
**MAP1B**	-1.230652771
**GAS2**	-1.229394091
**PSMC1**	-1.228399045
**DCAF1**	-1.227994142
**AC005943.6**	-1.227201057
**ACADVL**	-1.220453794
**RP11-483P21.2**	-1.22012425
**NDUFC1**	-1.219727483
**RP1-261G23.7**	-1.210368329
**TSSK2**	-1.208141519
**SMIM15**	-1.194794932
**SEMA4G**	-1.192197525
**STXBP5-AS1**	-1.191071783
**VAC14-AS1**	-1.184135709
**INE2**	-1.183377028

**RP4-734G22.3**	-1.183006117
**CTD-2555O16.4**	-1.168330161
**AC083884.8**	-1.167241525
**ASB8**	-1.164707904
**RP11-551L14.4**	-1.159808913
**LINC00867**	-1.159339612
**LINC01917**	-1.157973458
**DNAAF1**	-1.157354605
**RP11-7I15.3**	-1.155454175
**NPB**	-1.15354881
**AE000658.22**	-1.150203882
**ADGRL1**	-1.149301967
**SMIM4**	-1.145447792
**TMEM213**	-1.141972736
**KLC1**	-1.133205883
**GFAP**	-1.130647819
**ZHX3**	-1.126334404
**LYRM2**	-1.124945263
**MOCS1**	-1.124827807
**USP47**	-1.123153233
**DIS3**	-1.122420711
**TMED1**	-1.118522711
**ADCY7**	-1.110686308
**CTC-563A5.5**	-1.107141277
**RP11-265N6.2**	-1.106171127
**RNA5SP216**	-1.10515329
**RP11-110I1.6**	-1.100526563
**NLRC3**	1.101772922

**AKAP8**	1.103417151
**MAST3-AS1**	1.106526887
**HDAC7**	1.108076059
**CTD-2105E13.15**	1.132437903
**BBIP1**	1.134331568
**BAIAP2L2**	1.138792728
**KMO**	1.141381008
**CPM**	1.142023544
**AC002398.12**	1.142414841
**AC005330.2**	1.143203912
**CCDC183-AS1**	1.148226269
**AC114271.2**	1.150110452
**AP006621.8**	1.156723826
**TBCE**	1.157128875
**PPIB**	1.160889984
**MTFR1**	1.162373642
**ERAP1**	1.162412251
**RP11-152H18.3**	1.16253289
**RP11-787I22.3**	1.163026368
**RP11-286N22.14**	1.166295462
**CTD-2015H6.3**	1.167117985
**RNF169**	1.17151375
**RP11-50D9.3**	1.179951193
**RP11-530C5.1**	1.181101671
**PHKG1**	1.181854459
**CCNC**	1.182448529
**NTPCR**	1.189241766
**RPL10**	1.189635013
**MIR1282**	1.19445267
**NCOR1**	1.198176753
**CBR4**	1.202353927
**LINC00602**	1.203513365
**AC011558.5**	1.211008825
**ENAH**	1.214388596
**PDAP1**	1.217852417
**FLNB-AS1**	1.219614457
**PRRC2C**	1.221578658
**ZFYVE16**	1.223031581
**HOOK2**	1.226108558
**RP11-108L7.4**	1.229057872
**STAT1**	1.229259082
**RP11-573M3.3**	1.230319414
**LLNLR-285B5.1**	1.233329463
**SIX5**	1.233519995
**RIMKLBP2**	1.236021577
**RP11-256L6.3**	1.242806288
**ANKS1B**	1.243040284

**EPHA5**	1.245934014
**SLC16A8**	1.248306086
**RP11-344B5.4**	1.261725877
**RGS11**	1.275891124
**EMILIN3**	1.279564169
**MIR34AHG**	1.284424477
**GNB4**	1.293389861
**UBR5**	1.297113004
**POLDIP2**	1.298926031
**ZSWIM1**	1.30266727
**PAPLN**	1.310924985
**THAP7-AS1**	1.314790321
**GPR107**	1.316505684
**JMJD4**	1.316639886
**SRSF11**	1.319149182
**COA1**	1.321066368
**MYO5B**	1.323969552
**RP11-10A14.3**	1.324499809
**C10orf95**	1.32607817
**AF127577.10**	1.332672566
**MAVS**	1.332696839
**RP11-326A19.5**	1.335293648
**RP11-102N12.3**	1.335344124
**RP11-81K13.1**	1.339238972
**RPL30**	1.339698361
**TCEAL2**	1.34138264
**STK25**	1.341970112
**PSMG4**	1.343525566
**SHPRH**	1.343923766
**TMEM97**	1.344386625
**CARM1**	1.347250634
**MT-CYB**	1.351657505
**RP11-672L10.2**	1.354269046
**PCNX2**	1.359806379
**RAD21**	1.363929297
**DAAM1**	1.366436435
**PNISR**	1.366744969
**SLC19A1**	1.366975755
**GNG12-AS1**	1.367014465
**NCMAP-DT**	1.369901978
**CLDN22**	1.372977513
**USP45**	1.374847491
**TRIP11**	1.380272255
**CCND2-AS1**	1.381739114
**MARCHF7**	1.385250417
**PEF1**	1.38569829
**BDNF**	1.390349439

**TXLNA**	1.393836831
**RAB13**	1.394910629
**XX-15A10.1**	1.399139169
**RNASEH1P1**	1.402972712
**OPN1SW**	1.408578351
**ZBED5**	1.40861761
**ZNF551**	1.408742929
**ITPR1**	1.409240828
**RP11-632F7.4**	1.411143511
**POMGNT1**	1.411599265
**ZNF789**	1.412828993
**RP11-446H18.5**	1.415298822
**FAM13A**	1.416221949
**CTD-2240E14.4**	1.416341606
**RBBP4**	1.419482856
**RP11-23P13.6**	1.421790336
**RP11-319G6.1**	1.423805004
**RP11-152K4.2**	1.427231738
**BID**	1.429027824
**BTG3**	1.43386118
**CTB-79E8.2**	1.436607226
**CARHSP1**	1.438416983
**ANGPTL6**	1.440027078
**IFI16**	1.440305562
**RP4-742C19.13**	1.446702518
**KRR1**	1.453098765
**IGSF8**	1.456191524
**IRF1-AS1**	1.457075867
**LINC00960**	1.458340192
**RAPGEF3**	1.460125648
**PPIAP53**	1.476495733
**SPRYD4**	1.480985161
**POLR1B**	1.487773935
**CTB-176F20.3**	1.492574786
**SPON2**	1.493214319
**ARHGAP5**	1.494607823
**NSUN7**	1.496918598
**LRRC57**	1.500435785
**RP11-104H15.8**	1.503226425
**CTD-2017F17.2**	1.504458982
**RP11-573D15.9**	1.510058222
**RP11-152P23.2**	1.513867028
**EVA1B**	1.520677414
**RP11-108P20.3**	1.521031639
**ARSG**	1.522103525
**SPATA1**	1.522842149
**FKBP14-AS1**	1.525600438

**PIK3IP1**	1.532993622
**CASC11**	1.537469346
**RP1-12G14.7**	1.538264804
**MMP24**	1.540065311
**ZNF436**	1.543163368
**RP11-535A19.1**	1.549154556
**RP11-542C16.1**	1.549383071
**CTD-2008L17.2**	1.54953199
**ZNF142**	1.56688087
**ACOXL**	1.567044551
**RP1-138B7.5**	1.569150656
**COA3**	1.571013842
**CDKN2AIP**	1.577752995
**CLU**	1.578289561
**NKTR**	1.580135402
**RP3-412A9.16**	1.580258303
**BEAN1**	1.583136794
**IL21R**	1.586839939
**SHROOM3-AS1**	1.59664491
**MAL2-AS1**	1.601404993
**RP5-858B6.3**	1.605585711
**LINC02029**	1.606691944
**RP11-361L15.5**	1.610055687
**MT-TC**	1.613096755
**CCDC13-AS1**	1.613322354
**RP11-864I4.1**	1.614923535
**RP4-614O4.13**	1.621215828
**GIGYF2**	1.630515127
**EPN2**	1.633803843
**SMARCC2**	1.634113952
**EAF1**	1.639027808
**MT-TY**	1.640172124
**CTD-3193O13.12**	1.648796967
**FZD4**	1.649763639
**RP11-394J1.2**	1.660580929
**NAV2**	1.662832789
**PMM1**	1.663454628
**HEBP2**	1.666701537
**ZSCAN18**	1.670008168
**LIN7A**	1.673420485
**AC096574.5**	1.680191605
**BMP6**	1.680912869
**LTO1**	1.685787587
**MICAL1**	1.689249633
**ERMP1**	1.696405788
**PLD4**	1.701843927
**MT-RNR2**	1.707980422

**PNMA2**	1.712588828
**GCNT1**	1.726441456
**SLC25A1P5**	1.73280831
**RP11-6G22.1**	1.736292228
**ZNF461**	1.737133655
**RELL2**	1.737416339
**CTB-47B11.3**	1.739857028
**PCOLCE-AS1**	1.741327897
**MALAT1**	1.748399073
**NOP9**	1.74971537
**FMNL1**	1.752680145
**ADORA2B**	1.75958098
**MAFK**	1.776647187
**DENND1C**	1.776807294
**CTC-558O2.2**	1.804242147
**NECAB1**	1.805871361
**WDR54**	1.809479556
**NUDT3**	1.812339213
**RPS28**	1.815514355
**PDXP-DT**	1.83234368
**ZNF732**	1.845660231
**RP13-143G15.4**	1.851730342
**RUSF1**	1.863196042
**FAM234A**	1.874942315
**ELK4**	1.876393462
**USP22**	1.878400771
**ZNF575**	1.87970636
**LPIN2**	1.882683009
**RP11-96D1.8**	1.886823728
**CARD14**	1.896410477
**FAS**	1.901569446
**ICMT**	1.905020701
**ZMAT5**	1.909918169
**RGP1**	1.910590367
**LIPH**	1.917387253
**CLOCK**	1.919739563
**ZC3H10**	1.921011865
**SIDT2**	1.921243135
**BAZ2A**	1.922047329
**CD4**	1.925301497
**PAPPA**	1.931132639
**RP11-52L5.6**	1.934417206
**KATNA1**	1.934931468
**LSM4**	1.93809114
**PBXIP1**	1.940057754
**FBXO25**	1.944388028
**SLC7A6OS**	1.95598256

**RNF168**	1.956200414
**MACROH2A1**	1.960402204
**RP11-304C12.5**	1.960415857
**MAPK7**	1.965229341
**PDIA3**	1.965487073
**APBB2**	1.97465752
**NDUFV1**	1.97650938
**DIABLO**	1.976982505
**LDLRAD4**	1.978960146
**SCG2**	1.986380135
**WDR3**	1.989003424
**RP11-545P7.4**	1.99060538
**TINAG**	1.992454397
**MYL12-AS1**	1.998221694
**AC104532.4**	2.000511938
**RP5-881L22.5**	2.002889495
**GAS6-AS1**	2.008265544
**MT-TL1**	2.008905988
**HCFC1R1**	2.012678415
**RPL37A**	2.013716491
**ATP5PD**	2.01949262
**FXYD1**	2.02150988
**CSP2**	2.027476198
**ACAD8**	2.030870669
**CAPN12**	2.036017382
**YJEFN3**	2.043453289
**KCNH1**	2.050989496
**KLF9**	2.052398603
**CDCA7L**	2.070571788
**DLD**	2.08742768
**CTD-2325A15.5**	2.092176135
**RP11-130L8.1**	2.099695868
**CLMAT3**	2.100008464
**RP11-69I8.3**	2.101212857
**RP1-37C10.7**	2.104450589
**COL4A2**	2.11136585
**MUC3A**	2.112537765
**CENPS**	2.123287839
**COL4A1**	2.144365177
**SKI**	2.145950665
**ZNF790**	2.149820519
**FIBIN**	2.151235653
**MLXP1**	2.153219799
**MTERF4**	2.155922603
**NEFH**	2.157330392
**RP11-386G11.3**	2.158480419
**C17orf100**	2.160870006

**P2RX6**	2.164471491
**LINC02669**	2.166377513
**CTD-2516F10.2**	2.171491421
**KLF2P2**	2.171981262
**LPIN3**	2.177099065
**CMIP**	2.178477184
**RFPL1S**	2.179066643
**SYT1**	2.188388778
**MAML3**	2.188508569
**RP11-588H23.3**	2.193002872
**RP4-686C3.7**	2.196091777
**OGFRL1**	2.205911564
**COL4A2-AS1**	2.210913252
**RP11-627G18.1**	2.214559716
**AC002057.2**	2.21742661
**NMT1**	2.219142394
**ASPDH**	2.223162602
**MT-ND2**	2.223833302
**HRNR**	2.226068355
**RP11-290L1.2**	2.228322955
**RP3-330O12.5**	2.230599078
**FSTL1**	2.238440772
**TBX10**	2.241181031
**RAB6C**	2.243929606
**RP11-118B23.6**	2.245130636
**CYP2U1-AS1**	2.250565552
**C16orf70**	2.263066727
**SWT1**	2.272274179
**LUC7L3**	2.274288357
**C1orf159**	2.2765261
**PARPBP**	2.290433113
**ECHDC2**	2.295123922
**SPIDR**	2.306266668
**MDK**	2.312621872
**RP11-416N2.4**	2.318840387
**PHLPP2**	2.337763113
**MT-ND1**	2.343965587
**RP11-425A6.6**	2.347037476
**ERI2**	2.351272262
**RP11-301N24.6**	2.359695529
**UQCC2**	2.364067431
**P4HA2-AS1**	2.375523877
**SF3B2**	2.377994675
**RP11-307C12.12**	2.381053775
**FOSL2**	2.383782392
**AC005625.1**	2.385259396
**HNRNPA2B1**	2.388278203

**DESI1**	2.389381978
**LA16c-314G4.4**	2.403709779
**CTD-2162K18.3**	2.404346028
**MMP24OS**	2.407838718
**ISLR**	2.408760773
**MYH9**	2.409998027
**RP11-981G7.6**	2.414559959
**HSPG2**	2.418871193
**MFF-DT**	2.426282297
**NUDT8**	2.434910523
**LIPA**	2.436761344
**TPM1-AS**	2.437198307
**ABHD15**	2.442527966
**RP5-881P19.8**	2.443090189
**RP11-211G3.3**	2.453188059
**PRKG1**	2.454790565
**HS1BP3**	2.484404725
**CAVIN1**	2.490113536
**BLNK**	2.490763922
**RP11-156L14.1**	2.496386536
**CCDC152**	2.498681275
**MORN1**	2.49914406
**RP11-241K3.5**	2.50179562
**MTHFD2**	2.504798792
**SPARC**	2.507352522
**TSEN54**	2.515995422
**PTRH1**	2.518825246
**KRT8P26**	2.523156358
**SNRPD2**	2.526236425
**ZFP14**	2.531713192
**PCOLCE**	2.533316287
**MTERF2**	2.535890081
**AP000697.6**	2.540868522
**TP73-AS1**	2.543334894
**RPL29**	2.544131738
**TMEM120B**	2.544600073
**LZTS2**	2.549931788
**RP11-667F14.1**	2.561570602
**ZNF483**	2.563298324
**AC024592.9**	2.569603544
**LINCR-0001**	2.570660521
**ACTN1**	2.573474276
**LINC01597**	2.578301194
**PRICKLE2-DT**	2.59388045
**BDNF-AS**	2.597325185
**KAZN**	2.602157405
**RP11-84A19.3**	2.602578582

**COL5A1**	2.603510166
**ZNF273**	2.610263272
**RP11-108P20.2**	2.612923726
**ZNF718**	2.627963986
**MIR122HG**	2.629639445
**CYP1B1-AS1**	2.633825465
**CTD-2544N14.3**	2.638476771
**AL132709.1**	2.654576575
**ENG**	2.65583913
**MPHOSPH6P1**	2.657192925
**LINC02334**	2.658199092
**TSPEAR-AS2**	2.660420852
**CPNE1**	2.670844119
**PRUNE2**	2.680416902
**RTRAF**	2.681816292
**ANO8**	2.696735058
**CTA-992D9.8**	2.697219246
**RP1-197B17.3**	2.697626218
**RP11-183J19.1**	2.700683115
**RP11-589G9.1**	2.70070312
**COL3A1**	2.706990867
**CTB-13F3.1**	2.713985473
**CYP8B1**	2.718964781
**RP11-254F19.5**	2.719046333
**DDX54**	2.728363193
**KRT7**	2.736416606
**PCAT1**	2.746683919
**ATP8B4**	2.752513812
**RP11-70C1.3**	2.752930439
**HSPB7**	2.760404133
**COL11A1**	2.761419983
**MYO10**	2.763708149
**RP5-1148A21.3**	2.765710773
**ERCC2**	2.768718462
**GLRX5P2**	2.768972259
**KRT18**	2.772704306
**OR2AT4**	2.774338862
**ADCYAP1**	2.774863031
**CTB-102L5.9**	2.777471479
**MBNL1**	2.782295661
**HIVEP3**	2.783138214
**ITGA3**	2.787167113
**UTY**	2.790753258
**PRPH**	2.792050876
**AMD1P4**	2.792898747
**RIMS4**	2.793096085
**KCNA7**	2.798072771

**TNS1**	2.802025713
**BBS10**	2.804001007
**GATB**	2.805701708
**HSD17B12**	2.808643859
**NKG7**	2.815172403
**MDM2**	2.82021078
**CTA-941F9.10**	2.822407018
**RP11-265D17.2**	2.825385873
**MYC**	2.827883731
**ARRDC3-AS1**	2.828521591
**RP11-615I2.2**	2.838594552
**SLC52A1**	2.840641755
**RP11-672L10.3**	2.841703489
**RP11-370B11.4**	2.84990589
**PLAC9**	2.866685302
**RPL22**	2.874635696
**MTRNR2L8**	2.879671804
**COL9A2**	2.881978652
**CTB-60B18.12**	2.889103276
**MYO18B**	2.889911347
**INF2**	2.895190894
**SPATA32**	2.895701058
**B4GALT1-AS1**	2.897523473
**APOE**	2.898410072
**SMPD3**	2.900306308
**RP11-641A6.5**	2.901710647
**GADD45B**	2.906134999
**MYRFL**	2.909722402
**SIAH3**	2.910287405
**TRPM3**	2.915810378
**PSMD4**	2.917962896
**ID3**	2.919310908
**HSPB1**	2.92061074
**MORN4**	2.921741727
**DINOL**	2.923290552
**RP11-42O4.2**	2.92729711
**CALD1**	2.929396164
**AC005281.2**	2.935302235
**FIP1L1**	2.935355872
**RP11-205M5.3**	2.939923175
**CCN2**	2.943580263
**ARHGEF16**	2.945217104
**BBOX1**	2.956831177
**LOXL1**	2.963189844
**NKAIN4**	2.968000886
**BAG3**	2.983294723
**GS1-124K5.4**	2.993049807

**LINC02254**	2.997130634
**OPN5**	3.00435207
**RP11-92C4.3**	3.004570287
**USP32**	3.009394653
**IGFBP3**	3.028322606
**RSPH10B2**	3.034809153
**RP11-666A8.9**	3.03502174
**AC104809.2**	3.035677765
**TPM1**	3.037386678
**RP11-460N20.8**	3.038396269
**TMC3-AS1**	3.048531332
**RP11-893F2.15**	3.048651526
**FLJ16779**	3.049565785
**RP11-1085N6.5**	3.057585552
**RPS21P4**	3.057962153
**IFNWP19**	3.05799718
**RP11-221G19.1**	3.058654346
**RP11-428F8.2**	3.062616883
**EPB41**	3.062864788
**HDAC9**	3.063532725
**PCED1B-AS1**	3.067269927
**NFATC3**	3.077713885
**RP13-129E14.1**	3.080406915
**FRG1CP**	3.082636366
**ITGA5**	3.087515926
**SPAG4**	3.088260575
**SPTLC2**	3.099952214
**PINK1-AS**	3.101443918
**CHRNB1**	3.10145947
**RP11-797H7.5**	3.114530071
**LDLRAD2**	3.115290455
**ACTN4**	3.118038071
**LIF-AS2**	3.120395219
**D2HGDH**	3.12379875
**NEAT1**	3.124511052
**CTB-60B18.18**	3.133781707
**PLK3**	3.140009614
**XGY2**	3.140018551
**COLEC12**	3.14156055
**ZNF56**	3.141840413
**RP11-122G18.11**	3.14300618
**RP11-96D1.10**	3.14463129
**RP11-268F1.3**	3.145476439
**C8orf88**	3.157693252
**CHMP2B**	3.163865298
**RP11-6E9.4**	3.170969771
**XACT**	3.172710591

**RNF141**	3.184612508
**PLEKHG3**	3.185199304
**MYLK-AS1**	3.188116115
**TMEM245**	3.193089778
**AMTN**	3.19687679
**MTCL1**	3.198269243
**CDKN2B-AS1**	3.206061482
**ST7L**	3.21357096
**RP11-834C11.11**	3.218074929
**SPTB**	3.226331661
**HOATZ**	3.228269696
**PTP4A1**	3.232736679
**SLC8B1**	3.233174072
**GPR17**	3.235504022
**RP11-67L14.2**	3.240869046
**TMBIM4**	3.247103484
**OR7E12P**	3.251506485
**IGFL2-AS1**	3.252144216
**BRI3**	3.257757345
**IL1B**	3.264611175
**RP11-430H10.3**	3.266797578
**BNIP3P9**	3.276887522
**RP11-369E15.3**	3.279460014
**MT-TP**	3.280831565
**RP11-17A4.2**	3.286514794
**TTLL10-AS1**	3.287774106
**LINC00963**	3.301608282
**ZFY**	3.30638901
**CASC19**	3.310537008
**RP11-1077A2.2**	3.311975809
**TCFL5**	3.312209856
**OPN3**	3.313981709
**SAP30L-AS1**	3.328987671
**PAPPA-AS1**	3.329689432
**GFM1**	3.329886702
**AP001189.4**	3.34243581
**CTA-109P11.4**	3.343921056
**PITX2**	3.353241223
**BCL6-AS1**	3.355138307
**UBE2N**	3.356383881
**RP11-331G2.8**	3.367143181
**CSN3**	3.378900864
**ZP3**	3.393770876
**CTC-296K1.3**	3.394821146
**RP11-144I2.1**	3.39536688
**RP11-554D14.4**	3.399070536
**PTPN14**	3.40263991

**PPFIBP2**	3.417345442
**IFNLR1**	3.433898608
**RP11-135D11.2**	3.446003498
**PLXDC2**	3.458006865
**RP11-225H22.7**	3.46304418
**BRF2**	3.465227929
**CYP11A1**	3.469493906
**RP11-245K15.2**	3.477750905
**LINC02458**	3.483889524
**RP11-1110F20.1**	3.485442747
**SNHG8**	3.486663149
**FABP6-AS1**	3.48907558
**THUMPD1**	3.490270868
**PLEKHG1**	3.495744708
**CLDN4**	3.532763344
**RP11-12M5.3**	3.538988296
**CTD-3193O13.1**	3.544823373
**NCEH1**	3.555551199
**GULP1**	3.578760177
**NPR3**	3.580833532
**ASB16-AS1**	3.605424718
**RP3-413H6.3**	3.606090463
**NOC2L**	3.607559209
**RP11-810O3.2**	3.622273401
**CTB-85C5.2**	3.633443875
**TBPL1**	3.642111228
**CTD-2175A23.1**	3.657909549
**SRFBP1**	3.678495039
**CXCL14**	3.686064267
**CSPG4P12**	3.695240399
**RP11-299G20.2**	3.706672499
**RP11-893F2.5**	3.715328667
**PRICKLE3**	3.724736808
**HKDC1**	3.725818443
**GCSAM**	3.735095596
**RP11-460I13.2**	3.811389468
**RP1-259A10.2**	3.816084738
**BET1**	3.817804752
**CTD-2265O21.3**	3.8220185
**PRR27**	3.822975652
**RBMS1**	3.827441763
**TGFB3-AS1**	3.867014543
**SCGB3A2**	3.869801632
**RP11-90C4.1**	3.879473712
**TPM2**	3.897063365
**YIF1B**	3.932091126
**ARHGAP44-AS1**	3.988877917

**RP11-213H15.1**	4.020352951
**RP11-381K20.2**	4.034112427
**TARID**	4.042857201
**THBS1-AS1**	4.043866957
**LMX1A-AS2**	4.046458961
**RPL32P32**	4.065001703
**KNOP1P5**	4.109397866
**RP11-794P6.6**	4.120526491
**LINC02139**	4.127489327
**RP11-437J2.4**	4.146656168
**MAP3K14**	4.150775927
**VAX1**	4.16281856
**CTD-2561J22.5**	4.16599057
**AC003099.2**	4.205982611
**RP11-710F7.2**	4.211054407
**TRPA1**	4.226391341
**ANKS1A**	4.234972939
**IGFL2**	4.26541263
**SLC13A5**	4.265448614
**FSIP1**	4.27132178
**HDDC2**	4.285249167
**RP5-850O15.3**	4.32232585
**EYA4**	4.367567164
**RP11-720L2.4**	4.367706303
**GDF5-AS1**	4.426800126
**KRT8**	4.498839368
**RP11-629N8.5**	4.503813553
**GPR132**	4.517647338
**RP11-244F12.2**	4.533281473
**KLRF2**	4.53891912
**HELLPAR**	4.58246037
**SNCA**	4.589440546
**RP11-347D21.5**	4.676781371
**MYL9**	4.716115965
**HLCS**	4.743836665
**CBX5**	4.871170613
**LOXL1-AS1**	4.907459399
**RP11-242G5.1**	4.925400994
**KLRK1-AS1**	4.931968239
**ACTA2-AS1**	4.960988259
**RP11-510J16.5**	4.995969735
**TEX26-AS1**	5.019514765
**ACTC1**	5.047017436
**COL1A1**	5.049983509
**STMP1**	5.118489027
**CTD-2207P18.1**	5.125710322
**CLIC6**	5.307961339

**CTB-147C22.8**	5.354924023
**ANKRD45**	5.387686521
**HPS3**	5.471398709
**POLR2B**	5.537813154
**FEM1B**	5.663206186
**SMC5-DT**	5.688829488
**RP11-814P5.1**	5.769242146
**BLCAP**	5.804832265
**CTD-2369P2.8**	5.837948502
**RP11-307B6.3**	5.862895399
**RP11-125B2.1**	5.901735262
**RP11-233G1.8**	6.138641186
**RP11-25K24.3**	6.146520537
**TMEM139-AS1**	6.22162233
**CTC-327F10.4**	6.333512786
**PCSK7**	6.42212054
**KRT7-AS**	6.736197011
**IGFBP7-AS1**	7.219069517

Supplementary Table 3(a): Dysregulated Genes in homozygous *PINK1* mutant DA neurons. This table lists genes that exhibit significant dysregulation (log2 Fold Change (FC) > 1.1 and False Discovery Rate (FDR) < 0.05) in homozygous *PINK1* mutant DA neurons, compared to control neurons. Each gene is identified by its gene symbol, along with the log2FC value.

**Supplementary figure 3(b)**	
**Gene Id**	log2(FC)
**NLRC3**	1.101772922
**AKAP8**	1.103417151
**MAST3-AS1**	1.106526887
**HDAC7**	1.108076059
**CTD-2105E13.15**	1.132437903
**BBIP1**	1.134331568
**BAIAP2L2**	1.138792728
**KMO**	1.141381008
**CPM**	1.142023544
**AC002398.12**	1.142414841
**AC005330.2**	1.143203912
**CCDC183-AS1**	1.148226269
**AC114271.2**	1.150110452
**AP006621.8**	1.156723826
**TBCE**	1.157128875
**PPIB**	1.160889984
**MTFR1**	1.162373642
**ERAP1**	1.162412251
**RP11-152H18.3**	1.16253289
**RP11-787I22.3**	1.163026368
**RP11-286N22.14**	1.166295462

**CTD-2015H6.3**	1.167117985
**RNF169**	1.17151375
**RP11-50D9.3**	1.179951193
**RP11-530C5.1**	1.181101671
**PHKG1**	1.181854459
**CCNC**	1.182448529
**NTPCR**	1.189241766
**RPL10**	1.189635013
**MIR1282**	1.19445267
**NCOR1**	1.198176753
**CBR4**	1.202353927
**LINC00602**	1.203513365
**AC011558.5**	1.211008825
**ENAH**	1.214388596
**PDAP1**	1.217852417
**FLNB-AS1**	1.219614457
**PRRC2C**	1.221578658
**ZFYVE16**	1.223031581
**HOOK2**	1.226108558
**RP11-108L7.4**	1.229057872
**STAT1**	1.229259082
**RP11-573M3.3**	1.230319414
**LLNLR-285B5.1**	1.233329463
**SIX5**	1.233519995
**RIMKLBP2**	1.236021577
**RP11-256L6.3**	1.242806288
**ANKS1B**	1.243040284
**EPHA5**	1.245934014
**SLC16A8**	1.248306086
**RP11-344B5.4**	1.261725877
**RGS11**	1.275891124
**EMILIN3**	1.279564169
**MIR34AHG**	1.284424477
**GNB4**	1.293389861
**UBR5**	1.297113004
**POLDIP2**	1.298926031
**ZSWIM1**	1.30266727
**PAPLN**	1.310924985
**THAP7-AS1**	1.314790321
**GPR107**	1.316505684
**JMJD4**	1.316639886
**SRSF11**	1.319149182
**COA1**	1.321066368
**MYO5B**	1.323969552
**RP11-10A14.3**	1.324499809
**C10orf95**	1.32607817
**AF127577.10**	1.332672566

**MAVS**	1.332696839
**RP11-326A19.5**	1.335293648
**RP11-102N12.3**	1.335344124
**RP11-81K13.1**	1.339238972
**RPL30**	1.339698361
**TCEAL2**	1.34138264
**STK25**	1.341970112
**PSMG4**	1.343525566
**SHPRH**	1.343923766
**TMEM97**	1.344386625
**CARM1**	1.347250634
**MT-CYB**	1.351657505
**RP11-672L10.2**	1.354269046
**PCNX2**	1.359806379
**RAD21**	1.363929297
**DAAM1**	1.366436435
**PNISR**	1.366744969
**SLC19A1**	1.366975755
**GNG12-AS1**	1.367014465
**NCMAP-DT**	1.369901978
**CLDN22**	1.372977513
**USP45**	1.374847491
**TRIP11**	1.380272255
**CCND2-AS1**	1.381739114
**MARCHF7**	1.385250417
**PEF1**	1.38569829
**BDNF**	1.390349439
**TXLNA**	1.393836831
**RAB13**	1.394910629
**XX-15A10.1**	1.399139169
**RNASEH1P1**	1.402972712
**OPN1SW**	1.408578351
**ZBED5**	1.40861761
**ZNF551**	1.408742929
**ITPR1**	1.409240828
**RP11-632F7.4**	1.411143511
**POMGNT1**	1.411599265
**ZNF789**	1.412828993
**RP11-446H18.5**	1.415298822
**FAM13A**	1.416221949
**CTD-2240E14.4**	1.416341606
**RBBP4**	1.419482856
**RP11-23P13.6**	1.421790336
**RP11-319G6.1**	1.423805004
**RP11-152K4.2**	1.427231738
**BID**	1.429027824
**BTG3**	1.43386118

**CTB-79E8.2**	1.436607226
**CARHSP1**	1.438416983
**ANGPTL6**	1.440027078
**IFI16**	1.440305562
**RP4-742C19.13**	1.446702518
**KRR1**	1.453098765
**IGSF8**	1.456191524
**IRF1-AS1**	1.457075867
**LINC00960**	1.458340192
**RAPGEF3**	1.460125648
**PPIAP53**	1.476495733
**SPRYD4**	1.480985161
**POLR1B**	1.487773935
**CTB-176F20.3**	1.492574786
**SPON2**	1.493214319
**ARHGAP5**	1.494607823
**NSUN7**	1.496918598
**LRRC57**	1.500435785
**RP11-104H15.8**	1.503226425
**CTD-2017F17.2**	1.504458982
**RP11-573D15.9**	1.510058222
**RP11-152P23.2**	1.513867028
**EVA1B**	1.520677414
**RP11-108P20.3**	1.521031639
**ARSG**	1.522103525
**SPATA1**	1.522842149
**FKBP14-AS1**	1.525600438
**PIK3IP1**	1.532993622
**CASC11**	1.537469346
**RP1-12G14.7**	1.538264804
**MMP24**	1.540065311
**ZNF436**	1.543163368
**RP11-535A19.1**	1.549154556
**RP11-542C16.1**	1.549383071
**CTD-2008L17.2**	1.54953199
**ZNF142**	1.56688087
**ACOXL**	1.567044551
**RP1-138B7.5**	1.569150656
**COA3**	1.571013842
**CDKN2AIP**	1.577752995
**CLU**	1.578289561
**NKTR**	1.580135402
**RP3-412A9.16**	1.580258303
**BEAN1**	1.583136794
**IL21R**	1.586839939
**SHROOM3-AS1**	1.59664491
**MAL2-AS1**	1.601404993

**RP5-858B6.3**	1.605585711
**LINC02029**	1.606691944
**RP11-361L15.5**	1.610055687
**MT-TC**	1.613096755
**CCDC13-AS1**	1.613322354
**RP11-864I4.1**	1.614923535
**RP4-614O4.13**	1.621215828
**GIGYF2**	1.630515127
**EPN2**	1.633803843
**SMARCC2**	1.634113952
**EAF1**	1.639027808
**MT-TY**	1.640172124
**CTD-3193O13.12**	1.648796967
**FZD4**	1.649763639
**RP11-394J1.2**	1.660580929
**NAV2**	1.662832789
**PMM1**	1.663454628
**HEBP2**	1.666701537
**ZSCAN18**	1.670008168
**LIN7A**	1.673420485
**AC096574.5**	1.680191605
**BMP6**	1.680912869
**LTO1**	1.685787587
**MICAL1**	1.689249633
**ERMP1**	1.696405788
**PLD4**	1.701843927
**MT-RNR2**	1.707980422
**PNMA2**	1.712588828
**GCNT1**	1.726441456
**SLC25A1P5**	1.73280831
**RP11-6G22.1**	1.736292228
**ZNF461**	1.737133655
**RELL2**	1.737416339
**CTB-47B11.3**	1.739857028
**PCOLCE-AS1**	1.741327897
**MALAT1**	1.748399073
**NOP9**	1.74971537
**FMNL1**	1.752680145
**ADORA2B**	1.75958098
**MAFK**	1.776647187
**DENND1C**	1.776807294
**CTC-558O2.2**	1.804242147
**NECAB1**	1.805871361
**WDR54**	1.809479556
**NUDT3**	1.812339213
**RPS28**	1.815514355
**PDXP-DT**	1.83234368

**ZNF732**	1.845660231
**RP13-143G15.4**	1.851730342
**RUSF1**	1.863196042
**FAM234A**	1.874942315
**ELK4**	1.876393462
**USP22**	1.878400771
**ZNF575**	1.87970636
**LPIN2**	1.882683009
**RP11-96D1.8**	1.886823728
**CARD14**	1.896410477
**FAS**	1.901569446
**ICMT**	1.905020701
**ZMAT5**	1.909918169
**RGP1**	1.910590367
**LIPH**	1.917387253
**CLOCK**	1.919739563
**ZC3H10**	1.921011865
**SIDT2**	1.921243135
**BAZ2A**	1.922047329
**CD4**	1.925301497
**PAPPA**	1.931132639
**RP11-52L5.6**	1.934417206
**KATNA1**	1.934931468
**LSM4**	1.93809114
**PBXIP1**	1.940057754
**FBXO25**	1.944388028
**SLC7A6OS**	1.95598256
**RNF168**	1.956200414
**MACROH2A1**	1.960402204
**RP11-304C12.5**	1.960415857
**MAPK7**	1.965229341
**PDIA3**	1.965487073
**APBB2**	1.97465752
**NDUFV1**	1.97650938
**DIABLO**	1.976982505
**LDLRAD4**	1.978960146
**SCG2**	1.986380135
**WDR3**	1.989003424
**RP11-545P7.4**	1.99060538
**TINAG**	1.992454397
**MYL12-AS1**	1.998221694
**AC104532.4**	2.000511938
**RP5-881L22.5**	2.002889495
**GAS6-AS1**	2.008265544
**MT-TL1**	2.008905988
**HCFC1R1**	2.012678415
**RPL37A**	2.013716491

**ATP5PD**	2.01949262
**FXYD1**	2.02150988
**CSP2**	2.027476198
**ACAD8**	2.030870669
**CAPN12**	2.036017382
**YJEFN3**	2.043453289
**KCNH1**	2.050989496
**KLF9**	2.052398603
**CDCA7L**	2.070571788
**DLD**	2.08742768
**CTD-2325A15.5**	2.092176135
**RP11-130L8.1**	2.099695868
**CLMAT3**	2.100008464
**RP11-69I8.3**	2.101212857
**RP1-37C10.7**	2.104450589
**COL4A2**	2.11136585
**MUC3A**	2.112537765
**CENPS**	2.123287839
**COL4A1**	2.144365177
**SKI**	2.145950665
**ZNF790**	2.149820519
**FIBIN**	2.151235653
**MLXP1**	2.153219799
**MTERF4**	2.155922603
**NEFH**	2.157330392
**RP11-386G11.3**	2.158480419
**C17orf100**	2.160870006
**P2RX6**	2.164471491
**LINC02669**	2.166377513
**CTD-2516F10.2**	2.171491421
**KLF2P2**	2.171981262
**LPIN3**	2.177099065
**CMIP**	2.178477184
**RFPL1S**	2.179066643
**SYT1**	2.188388778
**MAML3**	2.188508569
**RP11-588H23.3**	2.193002872
**RP4-686C3.7**	2.196091777
**OGFRL1**	2.205911564
**COL4A2-AS1**	2.210913252
**RP11-627G18.1**	2.214559716
**AC002057.2**	2.21742661
**NMT1**	2.219142394
**ASPDH**	2.223162602
**MT-ND2**	2.223833302
**HRNR**	2.226068355
**RP11-290L1.2**	2.228322955

**RP3-330O12.5**	2.230599078
**FSTL1**	2.238440772
**TBX10**	2.241181031
**RAB6C**	2.243929606
**RP11-118B23.6**	2.245130636
**CYP2U1-AS1**	2.250565552
**C16orf70**	2.263066727
**SWT1**	2.272274179
**LUC7L3**	2.274288357
**C1orf159**	2.2765261
**PARPBP**	2.290433113
**ECHDC2**	2.295123922
**SPIDR**	2.306266668
**MDK**	2.312621872
**RP11-416N2.4**	2.318840387
**PHLPP2**	2.337763113
**MT-ND1**	2.343965587
**RP11-425A6.6**	2.347037476
**ERI2**	2.351272262
**RP11-301N24.6**	2.359695529
**UQCC2**	2.364067431
**P4HA2-AS1**	2.375523877
**SF3B2**	2.377994675
**RP11-307C12.12**	2.381053775
**FOSL2**	2.383782392
**AC005625.1**	2.385259396
**HNRNPA2B1**	2.388278203
**DESI1**	2.389381978
**LA16c-314G4.4**	2.403709779
**CTD-2162K18.3**	2.404346028
**MMP24OS**	2.407838718
**ISLR**	2.408760773
**MYH9**	2.409998027
**RP11-981G7.6**	2.414559959
**HSPG2**	2.418871193
**MFF-DT**	2.426282297
**NUDT8**	2.434910523
**LIPA**	2.436761344
**TPM1-AS**	2.437198307
**ABHD15**	2.442527966
**RP5-881P19.8**	2.443090189
**RP11-211G3.3**	2.453188059
**PRKG1**	2.454790565
**HS1BP3**	2.484404725
**CAVIN1**	2.490113536
**BLNK**	2.490763922
**RP11-156L14.1**	2.496386536

**CCDC152**	2.498681275
**MORN1**	2.49914406
**RP11-241K3.5**	2.50179562
**MTHFD2**	2.504798792
**SPARC**	2.507352522
**TSEN54**	2.515995422
**PTRH1**	2.518825246
**KRT8P26**	2.523156358
**SNRPD2**	2.526236425
**ZFP14**	2.531713192
**PCOLCE**	2.533316287
**MTERF2**	2.535890081
**AP000697.6**	2.540868522
**TP73-AS1**	2.543334894
**RPL29**	2.544131738
**TMEM120B**	2.544600073
**LZTS2**	2.549931788
**RP11-667F14.1**	2.561570602
**ZNF483**	2.563298324
**AC024592.9**	2.569603544
**LINCR-0001**	2.570660521
**ACTN1**	2.573474276
**LINC01597**	2.578301194
**PRICKLE2-DT**	2.59388045
**BDNF-AS**	2.597325185
**KAZN**	2.602157405
**RP11-84A19.3**	2.602578582
**COL5A1**	2.603510166
**ZNF273**	2.610263272
**RP11-108P20.2**	2.612923726
**ZNF718**	2.627963986
**MIR122HG**	2.629639445
**CYP1B1-AS1**	2.633825465
**CTD-2544N14.3**	2.638476771
**AL132709.1**	2.654576575
**ENG**	2.65583913
**MPHOSPH6P1**	2.657192925
**LINC02334**	2.658199092
**TSPEAR-AS2**	2.660420852
**CPNE1**	2.670844119
**PRUNE2**	2.680416902
**RTRAF**	2.681816292
**ANO8**	2.696735058
**CTA-992D9.8**	2.697219246
**RP1-197B17.3**	2.697626218
**RP11-183J19.1**	2.700683115
**RP11-589G9.1**	2.70070312

**COL3A1**	2.706990867
**CTB-13F3.1**	2.713985473
**CYP8B1**	2.718964781
**RP11-254F19.5**	2.719046333
**DDX54**	2.728363193
**KRT7**	2.736416606
**PCAT1**	2.746683919
**ATP8B4**	2.752513812
**RP11-70C1.3**	2.752930439
**HSPB7**	2.760404133
**COL11A1**	2.761419983
**MYO10**	2.763708149
**RP5-1148A21.3**	2.765710773
**ERCC2**	2.768718462
**GLRX5P2**	2.768972259
**KRT18**	2.772704306
**OR2AT4**	2.774338862
**ADCYAP1**	2.774863031
**CTB-102L5.9**	2.777471479
**MBNL1**	2.782295661
**HIVEP3**	2.783138214
**ITGA3**	2.787167113
**UTY**	2.790753258
**PRPH**	2.792050876
**AMD1P4**	2.792898747
**RIMS4**	2.793096085
**KCNA7**	2.798072771
**TNS1**	2.802025713
**BBS10**	2.804001007
**GATB**	2.805701708
**HSD17B12**	2.808643859
**NKG7**	2.815172403
**MDM2**	2.82021078
**CTA-941F9.10**	2.822407018
**RP11-265D17.2**	2.825385873
**MYC**	2.827883731
**ARRDC3-AS1**	2.828521591
**RP11-615I2.2**	2.838594552
**SLC52A1**	2.840641755
**RP11-672L10.3**	2.841703489
**RP11-370B11.4**	2.84990589
**PLAC9**	2.866685302
**RPL22**	2.874635696
**MTRNR2L8**	2.879671804
**COL9A2**	2.881978652
**CTB-60B18.12**	2.889103276
**MYO18B**	2.889911347

**INF2**	2.895190894
**SPATA32**	2.895701058
**B4GALT1-AS1**	2.897523473
**APOE**	2.898410072
**SMPD3**	2.900306308
**RP11-641A6.5**	2.901710647
**GADD45B**	2.906134999
**MYRFL**	2.909722402
**SIAH3**	2.910287405
**TRPM3**	2.915810378
**PSMD4**	2.917962896
**ID3**	2.919310908
**HSPB1**	2.92061074
**MORN4**	2.921741727
**DINOL**	2.923290552
**RP11-42O4.2**	2.92729711
**CALD1**	2.929396164
**AC005281.2**	2.935302235
**FIP1L1**	2.935355872
**RP11-205M5.3**	2.939923175
**CCN2**	2.943580263
**ARHGEF16**	2.945217104
**BBOX1**	2.956831177
**LOXL1**	2.963189844
**NKAIN4**	2.968000886
**BAG3**	2.983294723
**GS1-124K5.4**	2.993049807
**LINC02254**	2.997130634
**OPN5**	3.00435207
**RP11-92C4.3**	3.004570287
**USP32**	3.009394653
**IGFBP3**	3.028322606
**RSPH10B2**	3.034809153
**RP11-666A8.9**	3.03502174
**AC104809.2**	3.035677765
**TPM1**	3.037386678
**RP11-460N20.8**	3.038396269
**TMC3-AS1**	3.048531332
**RP11-893F2.15**	3.048651526
**FLJ16779**	3.049565785
**RP11-1085N6.5**	3.057585552
**RPS21P4**	3.057962153
**IFNWP19**	3.05799718
**RP11-221G19.1**	3.058654346
**RP11-428F8.2**	3.062616883
**EPB41**	3.062864788
**HDAC9**	3.063532725

**PCED1B-AS1**	3.067269927
**NFATC3**	3.077713885
**RP13-129E14.1**	3.080406915
**FRG1CP**	3.082636366
**ITGA5**	3.087515926
**SPAG4**	3.088260575
**SPTLC2**	3.099952214
**PINK1-AS**	3.101443918
**CHRNB1**	3.10145947
**RP11-797H7.5**	3.114530071
**LDLRAD2**	3.115290455
**ACTN4**	3.118038071
**LIF-AS2**	3.120395219
**D2HGDH**	3.12379875
**NEAT1**	3.124511052
**CTB-60B18.18**	3.133781707
**PLK3**	3.140009614
**XGY2**	3.140018551
**COLEC12**	3.14156055
**ZNF56**	3.141840413
**RP11-122G18.11**	3.14300618
**RP11-96D1.10**	3.14463129
**RP11-268F1.3**	3.145476439
**C8orf88**	3.157693252
**CHMP2B**	3.163865298
**RP11-6E9.4**	3.170969771
**XACT**	3.172710591
**RNF141**	3.184612508
**PLEKHG3**	3.185199304
**MYLK-AS1**	3.188116115
**TMEM245**	3.193089778
**AMTN**	3.19687679
**MTCL1**	3.198269243
**CDKN2B-AS1**	3.206061482
**ST7L**	3.21357096
**RP11-834C11.11**	3.218074929
**SPTB**	3.226331661
**HOATZ**	3.228269696
**PTP4A1**	3.232736679
**SLC8B1**	3.233174072
**GPR17**	3.235504022
**RP11-67L14.2**	3.240869046
**TMBIM4**	3.247103484
**OR7E12P**	3.251506485
**IGFL2-AS1**	3.252144216
**BRI3**	3.257757345
**IL1B**	3.264611175

**RP11-430H10.3**	3.266797578
**BNIP3P9**	3.276887522
**RP11-369E15.3**	3.279460014
**MT-TP**	3.280831565
**RP11-17A4.2**	3.286514794
**TTLL10-AS1**	3.287774106
**LINC00963**	3.301608282
**ZFY**	3.30638901
**CASC19**	3.310537008
**RP11-1077A2.2**	3.311975809
**TCFL5**	3.312209856
**OPN3**	3.313981709
**SAP30L-AS1**	3.328987671
**PAPPA-AS1**	3.329689432
**GFM1**	3.329886702
**AP001189.4**	3.34243581
**CTA-109P11.4**	3.343921056
**PITX2**	3.353241223
**BCL6-AS1**	3.355138307
**UBE2N**	3.356383881
**RP11-331G2.8**	3.367143181
**CSN3**	3.378900864
**ZP3**	3.393770876
**CTC-296K1.3**	3.394821146
**RP11-144I2.1**	3.39536688
**RP11-554D14.4**	3.399070536
**PTPN14**	3.40263991
**PPFIBP2**	3.417345442
**IFNLR1**	3.433898608
**RP11-135D11.2**	3.446003498
**PLXDC2**	3.458006865
**RP11-225H22.7**	3.46304418
**BRF2**	3.465227929
**CYP11A1**	3.469493906
**RP11-245K15.2**	3.477750905
**LINC02458**	3.483889524
**RP11-1110F20.1**	3.485442747
**SNHG8**	3.486663149
**FABP6-AS1**	3.48907558
**THUMPD1**	3.490270868
**PLEKHG1**	3.495744708
**CLDN4**	3.532763344
**RP11-12M5.3**	3.538988296
**CTD-3193O13.1**	3.544823373
**NCEH1**	3.555551199
**GULP1**	3.578760177
**NPR3**	3.580833532

**ASB16-AS1**	3.605424718
**RP3-413H6.3**	3.606090463
**NOC2L**	3.607559209
**RP11-810O3.2**	3.622273401
**CTB-85C5.2**	3.633443875
**TBPL1**	3.642111228
**CTD-2175A23.1**	3.657909549
**SRFBP1**	3.678495039
**CXCL14**	3.686064267
**CSPG4P12**	3.695240399
**RP11-299G20.2**	3.706672499
**RP11-893F2.5**	3.715328667
**PRICKLE3**	3.724736808
**HKDC1**	3.725818443
**GCSAM**	3.735095596
**RP11-460I13.2**	3.811389468
**RP1-259A10.2**	3.816084738
**BET1**	3.817804752
**CTD-2265O21.3**	3.8220185
**PRR27**	3.822975652
**RBMS1**	3.827441763
**TGFB3-AS1**	3.867014543
**SCGB3A2**	3.869801632
**RP11-90C4.1**	3.879473712
**TPM2**	3.897063365
**YIF1B**	3.932091126
**ARHGAP44-AS1**	3.988877917
**RP11-213H15.1**	4.020352951
**RP11-381K20.2**	4.034112427
**TARID**	4.042857201
**THBS1-AS1**	4.043866957
**LMX1A-AS2**	4.046458961
**RPL32P32**	4.065001703
**KNOP1P5**	4.109397866
**RP11-794P6.6**	4.120526491
**LINC02139**	4.127489327
**RP11-437J2.4**	4.146656168
**MAP3K14**	4.150775927
**VAX1**	4.16281856
**CTD-2561J22.5**	4.16599057
**AC003099.2**	4.205982611
**RP11-710F7.2**	4.211054407
**TRPA1**	4.226391341
**ANKS1A**	4.234972939
**IGFL2**	4.26541263
**SLC13A5**	4.265448614
**FSIP1**	4.27132178

**HDDC2**	4.285249167
**RP5-850O15.3**	4.32232585
**EYA4**	4.367567164
**RP11-720L2.4**	4.367706303
**GDF5-AS1**	4.426800126
**KRT8**	4.498839368
**RP11-629N8.5**	4.503813553
**GPR132**	4.517647338
**RP11-244F12.2**	4.533281473
**KLRF2**	4.53891912
**HELLPAR**	4.58246037
**SNCA**	4.589440546
**RP11-347D21.5**	4.676781371
**MYL9**	4.716115965
**HLCS**	4.743836665
**CBX5**	4.871170613
**LOXL1-AS1**	4.907459399
**RP11-242G5.1**	4.925400994
**KLRK1-AS1**	4.931968239
**ACTA2-AS1**	4.960988259
**RP11-510J16.5**	4.995969735
**TEX26-AS1**	5.019514765
**ACTC1**	5.047017436
**COL1A1**	5.049983509
**STMP1**	5.118489027
**CTD-2207P18.1**	5.125710322
**CLIC6**	5.307961339
**CTB-147C22.8**	5.354924023
**ANKRD45**	5.387686521
**HPS3**	5.471398709
**POLR2B**	5.537813154
**FEM1B**	5.663206186
**SMC5-DT**	5.688829488
**RP11-814P5.1**	5.769242146
**BLCAP**	5.804832265
**CTD-2369P2.8**	5.837948502
**RP11-307B6.3**	5.862895399
**RP11-125B2.1**	5.901735262
**RP11-233G1.8**	6.138641186
**RP11-25K24.3**	6.146520537
**TMEM139-AS1**	6.22162233
**CTC-327F10.4**	6.333512786
**PCSK7**	6.42212054
**KRT7-AS**	6.736197011
**IGFBP7-AS1**	7.219069517

Supplementary Table 3(b): Upregulated Genes in homozygous *PINK1* mutant DA neurons. This table lists genes that exhibit significant upregulation (log2 Fold Change (FC) >

1.1 and False Discovery Rate (FDR) < 0.05) in homozygous *PINK1* mutant DA neurons, compared to control neurons. Each gene is identified by its gene symbol, along with the log2FC value.

**Supplementary table 3(c)**	
**Gene Id**	log2FC
**EXOC6**	-5.405677811
**RP11-482D24.3**	-5.367352939
**ENSG10010137930.1**	-5.248384302
**RP11-547I7.1**	-5.166396542
**ENSG10010137683.1**	-4.967944006
**OFD1**	-4.932170129
**ZDHHC8**	-4.81656645
**RP11-482D24.2**	-4.788751405
**HOXA10-AS**	-4.737610219
**FAM172A**	-4.667594178
**RP11-284F21.10**	-4.661955343
**SOX21-AS1**	-4.53269563
**RP5-872K7.8**	-4.47535043
**LINC00621**	-4.470249015
**HOXB3**	-4.456810712
**LRBA**	-4.448099466
**AC005481.5**	-4.444310576
**AKAP13**	-4.384201174
**ACBD6**	-4.383208303
**LINC00944**	-4.377312506
**MEIS1-AS2**	-4.36367608
**RP1-18D14.7**	-4.307743908
**LIMS2**	-4.276989966
**ZEB2-AS1**	-4.206327087
**RP11-435O5.5**	-4.149354867

**GATA3-AS1**	-4.113744612
**RP3-514A23.4**	-4.104586363
**RHCG**	-4.092463152
**KIF7**	-4.079503323
**RP11-380I10.4**	-4.069238513
**SLC32A1**	-3.973862774
**HOXA2**	-3.936908545
**DELEC1**	-3.910349581
**POU3F4**	-3.884091678
**URM1**	-3.865603573
**LINC01361**	-3.865501862
**TSTD2**	-3.859747855
**AFDN-DT**	-3.844297846
**HOXA10**	-3.784836866
**HADHA**	-3.737598232
**LINC01798**	-3.715473375
**RP11-1000B6.9**	-3.631283444
**CELSR2**	-3.60533905
**LCOR**	-3.58793148
**CTD-2510F5.4**	-3.587037687
**GALR1**	-3.582803918
**CTD-2015C24.1**	-3.519658418
**RP5-1055C14.9**	-3.492764621
**NT5DC4**	-3.492600461
**FGFBP3**	-3.492158813
**SYN2**	-3.47484052
**RP13-238F13.3**	-3.46448606
**RP4-694A7.2**	-3.451959167

**LINC00928**	-3.438777125
**AC007563.5**	-3.437027425
**RP11-234G16.4**	-3.424187602
**ERBIN**	-3.361193628
**LHX5**	-3.356075821
**USP17L2**	-3.336023484
**RP11-422N16.3**	-3.302803594
**RP5-998C11.1**	-3.249298511
**IDH3A**	-3.243871753
**HAGLR**	-3.235353262
**AC004160.4**	-3.16883494
**FOXD1-AS1**	-3.163236244
**SGO1-AS1**	-3.153066636
**TNFRSF25**	-3.138905842
**CCDC103**	-3.117872692
**CTD-3094K11.3**	-3.102041908
**RP11-49K24.3**	-3.045958054
**GRM5-AS1**	-3.040868375
**CTD-2636A23.2**	-3.008085421
**ITFG2**	-3.00008304
**RP11-154I21.1**	-2.982308184
**AC053503.6**	-2.964477215
**LCORL**	-2.957792165
**TPT1-AS1**	-2.891596238
**ZEB2**	-2.880904446
**EFCC1**	-2.868937274
**RP11-435D7.3**	-2.865945028

**HEPN1**	-2.861179174
**AC004538.3**	-2.844629779
**TCP1**	-2.843503936
**GS1-72M22.1**	-2.831304593
**GRM3-AS1**	-2.818479734
**PAX8-AS1**	-2.814002022
**HOXB-AS2**	-2.811603443
**HOXA-AS2**	-2.811335911
**SHH**	-2.808489808
**FABP7**	-2.802614056
**DCDC2**	-2.799484904
**POLG**	-2.78756239
**RGMB-AS1**	-2.787089215
**NTRK2**	-2.775797652
**ITGB8-AS1**	-2.766493329
**NDP-AS1**	-2.765199889
**NR2F1-AS1**	-2.763152457
**TMEM161B-AS1**	-2.748374894
**RP11-128A17.1**	-2.744780231
**ALDH3A2**	-2.730970418
**AC008746.3**	-2.701855604
**MYO1E**	-2.695526131
**ALG12**	-2.69499968
**RP11-318N11.1**	-2.690228776
**ITGAL**	-2.687425281
**XXyac-YX65C7_A.2**	-2.685200994
**RP11-6O2.4**	-2.681412094

**MEIS1**	-2.680962078
**RP11-397O8.7**	-2.676086712
**HOXB-AS1**	-2.634142482
**SRPX2**	-2.633934072
**RP5-955M13.4**	-2.63093198
**KAAG1**	-2.626448693
**GNAS-AS1**	-2.61715628
**AC068057.1**	-2.614221252
**BAHCC1**	-2.590416791
**CLCC1**	-2.58891711
**INHBE**	-2.571038095
**PRC1-AS1**	-2.555298914
**CTD-2215L10.1**	-2.553744081
**CHRM5**	-2.553374003
**AP001469.5**	-2.546906994
**ATP2C2-AS1**	-2.546278414
**ERRFI1**	-2.515778119
**KLB**	-2.509337439
**RP11-234G16.6**	-2.49254543
**TNC**	-2.481627596
**RP11-342C2.2**	-2.481241795
**INTS8**	-2.477342187
**SNRPGP10**	-2.473217088
**RP4-555D20.1**	-2.465348952
**RP11-379F12.4**	-2.454432233
**RP4-671O14.5**	-2.448293563
**RP11-34F13.2**	-2.444457479
**NR2F2**	-2.412018329

**SMAP1**	-2.401022957
**DCHS2**	-2.38783771
**POU3F3**	-2.379342656
**RP11-25K19.1**	-2.348399576
**CD44-AS1**	-2.328554833
**HAS2-AS1**	-2.321843434
**MPEG1**	-2.318784777
**SHC3**	-2.295326501
**AC093390.1**	-2.285503444
**RP11-481J2.3**	-2.276651568
**FGF13-AS1**	-2.268449924
**RP11-73E17.2**	-2.25845021
**AGGF1**	-2.247423565
**LINC00511**	-2.24419447
**FADS1**	-2.243273682
**RP11-275G7.2**	-2.240574476
**RP11-567G24.3**	-2.230205256
**RP11-427I6.5**	-2.226937484
**TUBB1**	-2.217759433
**CDR1**	-2.207470185
**DENND1A**	-2.207321747
**DAAM2-AS1**	-2.203858398
**NDUFA4L2**	-2.196034735
**SEMA4D**	-2.192404654
**CCP110**	-2.190661968
**RP11-552F3.9**	-2.182009615
**TTC23**	-2.170338299
**FGF13**	-2.166618132

**CACNA2D3-AS1**	-2.159830106
**RP11-290O12.2**	-2.159710777
**PER3**	-2.15208
**TRIM59**	-2.149556662
**OIP5-AS1**	-2.146956083
**ADCY3**	-2.142214922
**MCF2L2**	-2.140972603
**RP11-353N14.7**	-2.125339567
**TECR**	-2.121119584
**NES**	-2.11547064
**ZBTB20-AS1**	-2.109206998
**RP11-1055B8.8**	-2.105608641
**RP11-342D11.2**	-2.097240455
**ZFPM1**	-2.097237153
**TMEM186**	-2.091871119
**RP11-12J10.4**	-2.08921809
**GS1-34D21.1**	-2.087204473
**NRXN2-AS1**	-2.086311431
**KLHL34**	-2.084804521
**NCOA3**	-2.074279078
**DUX4L50**	-2.069508311
**YIF1A**	-2.063822847
**RP11-732A19.6**	-2.063333641
**MGC32805**	-2.060389639
**RP11-284H19.1**	-2.056241377
**CTD-2353F22.1**	-2.05610727
**SEMA6A-AS1**	-2.029407728
**C12orf65**	-2.027357786

**CASK-AS1**	-2.013473357
**RP11-4N23.1**	-2.012276066
**RSRC1**	-2.010808952
**RP11-325J6.2**	-2.00895762
**LINC00200**	-2.004225397
**PPP1R12B**	-1.994164249
**RP11-74H8.1**	-1.983357744
**RP11-989F5.1**	-1.980946108
**NTN1**	-1.980533609
**RECQL4**	-1.979913214
**CENPN-AS1**	-1.977706346
**MID1IP1-AS1**	-1.967032922
**PAH**	-1.957575006
**UROD**	-1.944929105
**CTC-463N11.4**	-1.944188952
**RP5-896L10.1**	-1.939755313
**PIGZ**	-1.932125548
**IFT20**	-1.930138735
**MARCKS**	-1.927761164
**CERT1**	-1.923998984
**AC008067.2**	-1.917443162
**SYTL4**	-1.916865439
**SLC25A26**	-1.915555777
**DDX20**	-1.914403415
**AC007405.8**	-1.913536738
**FANCD2OS**	-1.91236296
**RP11-435O5.4**	-1.911262701

**GDE1**	-1.898249648
**TMEM51-AS1**	-1.887727563
**SPATA46**	-1.884228722
**RP11-344B2.2**	-1.883716523
**CEP57**	-1.88251036
**QKI**	-1.873659524
**AC007326.9**	-1.872458471
**TACC3**	-1.869742425
**NADK2-AS1**	-1.863998027
**RP11-360L9.7**	-1.86177615
**PAQR4**	-1.856890005
**LRRC41**	-1.856288747
**AKAP9**	-1.850491502
**CFAP44**	-1.848558667
**LRRC7-AS1**	-1.847206664
**WDFY3-AS2**	-1.834772042
**C11orf42**	-1.825892433
**RP11-261N11.8**	-1.823527648
**RP11-196H14.2**	-1.821015205
**NUP62**	-1.810415462
**RP11-1140I5.2**	-1.799626862
**KCNK12**	-1.796774402
**RP11-144F15.1**	-1.793759229
**AC037445.1**	-1.78668077
**EXOSC9**	-1.785808311
**Z82214.2**	-1.784634992
**PRORP**	-1.781794512
**RP11-146F11.1**	-1.776484324

**RNF2P1**	-1.773281463
**TSIX**	-1.772727856
**MARS1**	-1.76503967
**RP11-113H14.3**	-1.761487488
**RP11-626E13.1**	-1.75857086
**RP11-9N12.2**	-1.757342337
**PXN**	-1.751678669
**VIM-AS1**	-1.743751247
**PSMB7**	-1.741414653
**RP11-862L9.3**	-1.735828003
**TRAPPC2**	-1.733061626
**NPAS2-AS1**	-1.731763503
**AATK**	-1.725398037
**AC009480.3**	-1.721713207
**RP11-272L14.3**	-1.720218372
**SGPP2**	-1.716989441
**RP11-124N14.3**	-1.716560911
**AP1S2**	-1.703997389
**RP4-541C22.5**	-1.701593302
**CAB39**	-1.69444934
**RP11-5O23.2**	-1.678151434
**CTC-250P22.2**	-1.665079862
**RP11-315D16.4**	-1.653531577
**RP11-95F22.1**	-1.64507492
**FAM86B1**	-1.643548397
**CTD-2653D5.1**	-1.642446441
**PTPRN**	-1.633004345
**RP11-192H23.5**	-1.631211507

**MST1**	-1.630447071
**IGFBP5**	-1.620998284
**SKIDA1**	-1.619459095
**ASPM**	-1.618738099
**NR2F1**	-1.608404406
**CTA-363E6.6**	-1.603788501
**CTD-2555O16.2**	-1.600662634
**RP11-296L22.8**	-1.58741101
**ITGBL1**	-1.58321056
**ACMSD**	-1.576895224
**CTSB**	-1.57385692
**LYPD1**	-1.569996638
**GSE1**	-1.568252021
**UCHL5**	-1.559158778
**RP4-784A16.2**	-1.554964156
**RNF217-AS1**	-1.553566257
**KATNAL2**	-1.55120219
**AC009133.15**	-1.550733296
**RP11-573D15.8**	-1.55024837
**IDI2-AS1**	-1.545901822
**YPEL4**	-1.543473289
**DHX38**	-1.542257871
**IPPK**	-1.531977424
**SCYL3**	-1.523823614
**PCA3**	-1.520343006
**A1BG-AS1**	-1.51989502
**VPS35L**	-1.516810967
**RP11-755H23.1**	-1.514184137
**RP11-945C19.4**	-1.511123906

**RP11-103J8.1**	-1.510963479
**FN1**	-1.505857367
**RP11-780K2.1**	-1.50316886
**TRIM37**	-1.499339593
**RP11-149P14.2**	-1.499148109
**ZMYM4-AS1**	-1.498777665
**SHLD2**	-1.498166137
**RBIS**	-1.494806969
**NDUFB9**	-1.494171269
**CTD-2619J13.9**	-1.493687218
**WDFY3-AS1**	-1.490616093
**RP11-536C5.7**	-1.486316703
**RP11-33O4.3**	-1.481858329
**AC016738.4**	-1.477671711
**CNTFR-AS1**	-1.466194436
**SCN1A-AS1**	-1.4594642
**RP5-966M1.7**	-1.456741341
**FAM198B-AS1**	-1.4566521
**AF196970.3**	-1.454603555
**WWTR1-AS1**	-1.449905388
**MYCL-AS1**	-1.447398922
**SLC9A7**	-1.446262262
**UBXN6**	-1.445558016
**FADS2**	-1.441221591
**RP3-512E2.2**	-1.441064537
**LRRC8C-DT**	-1.43612079
**AF131216.7**	-1.430898934
**XXbac-B562F10.11**	-1.429010678

**RP11-229P13.23**	-1.415318256
**NCAM1-AS1**	-1.414294024
**RP11-59C5.3**	-1.407149532
**ATXN1L**	-1.405164118
**AC022154.7**	-1.398143853
**LUCAT1**	-1.393360068
**RP11-204E9.3**	-1.391901128
**RP11-815J21.2**	-1.390725067
**GAD1**	-1.390658084
**LA16c-395F10.1**	-1.388114144
**BRAT1**	-1.387529924
**AC012363.4**	-1.387381653
**TMPO-AS1**	-1.384004953
**AC004490.1**	-1.382292268
**NUP153-AS1**	-1.377157695
**VIM2P**	-1.376823364
**TPSG1**	-1.37536817
**SUPT20H**	-1.374913102
**RP5-991G20.1**	-1.362081739
**PLEKHJ1**	-1.360688028
**RP11-467K18.2**	-1.357924817
**RP11-420L9.5**	-1.354828477
**RP11-394B2.1**	-1.35368258
**RAP2C-AS1**	-1.35196321
**TTN**	-1.34943287
**JAKMIP2-AS1**	-1.346522148
**TLCD3A**	-1.334625511
**RP11-830F9.5**	-1.333434094

**ZNHIT2**	-1.332954272
**RP11-16C18.3**	-1.331012413
**DDB2**	-1.324740599
**ENTPD1-AS1**	-1.315211246
**RP11-95O2.5**	-1.312487328
**AC009133.21**	-1.311384419
**C9orf16**	-1.3099921
**GPR39**	-1.309744726
**RP11-845C23.3**	-1.308856081
**RP5-1103B4.3**	-1.308071776
**CEP250-AS1**	-1.306884911
**CTD-3032J10.2**	-1.303188754
**VIM**	-1.300610331
**DCHS1-AS1**	-1.295031929
**RP11-4N23.4**	-1.294340739
**RP11-558B7.1**	-1.285723657
**ZNF862**	-1.28154806
**CTB-187M2.2**	-1.276706919
**RP11-234O6.2**	-1.275422803
**RP11-463O12.3**	-1.273017068
**GLB1L2**	-1.27183381
**ZNF276**	-1.26811891
**RP11-300E4.2**	-1.265479056
**THBS2**	-1.265036881
**RP11-226E21.4**	-1.262253053
**SKP2**	-1.260296258
**DNAJC11**	-1.259481045
**RP11-544A12.4**	-1.255558463

**RP11-379K22.3**	-1.254393831
**FAM169A**	-1.253637739
**NCAPD3**	-1.24737902
**MCPH1-AS1**	-1.247097425
**RP11-513M16.8**	-1.247007866
**SPRN**	-1.243754524
**RP4-678D15.1**	-1.243350753
**ZNF710-AS1**	-1.236762863
**TIGD7**	-1.230742559
**MAP1B**	-1.230652771
**GAS2**	-1.229394091
**PSMC1**	-1.228399045
**DCAF1**	-1.227994142
**AC005943.6**	-1.227201057
**ACADVL**	-1.220453794
**RP11-483P21.2**	-1.22012425
**NDUFC1**	-1.219727483
**RP1-261G23.7**	-1.210368329
**TSSK2**	-1.208141519
**SMIM15**	-1.194794932
**SEMA4G**	-1.192197525
**STXBP5-AS1**	-1.191071783
**VAC14-AS1**	-1.184135709
**INE2**	-1.183377028
**RP4-734G22.3**	-1.183006117
**CTD-2555O16.4**	-1.168330161
**AC083884.8**	-1.167241525

**ASB8**	-1.164707904
**RP11-551L14.4**	-1.159808913
**LINC00867**	-1.159339612
**LINC01917**	-1.157973458
**DNAAF1**	-1.157354605
**RP11-7I15.3**	-1.155454175
**NPB**	-1.15354881
**AE000658.22**	-1.150203882
**ADGRL1**	-1.149301967
**SMIM4**	-1.145447792
**TMEM213**	-1.141972736
**KLC1**	-1.133205883
**GFAP**	-1.130647819
**ZHX3**	-1.126334404
**LYRM2**	-1.124945263
**MOCS1**	-1.124827807
**USP47**	-1.123153233
**DIS3**	-1.122420711
**TMED1**	-1.118522711
**ADCY7**	-1.110686308
**CTC-563A5.5**	-1.107141277
**RP11-265N6.2**	-1.106171127
**RNA5SP216**	-1.10515329
**RP11-110I1.6**	-1.100526563

Supplementary Table 3(c): Downregulated Genes in homozygous *PINK1* mutant DA neurons. This table lists genes that exhibit significant downregulation (log2 Fold Change (FC) > 1.1 and False Discovery Rate (FDR) < 0.05) in homozygous *PINK1* mutant DA neurons, compared to control neurons. Each gene is identified by its gene symbol, along with the log2FC value.

Mutation	Disease status	Additional cell types	iPSC line name / notes	Sex	Age at sampling	Relationship with other subjects	Nationality / Ethnicity
PARK2 R275W/WT + PINK1 p.Try90Leu fs*12/WT	Affected	iPS	CENSOi024-A	Male	75	familial PD #156, #157, #72	Caucasian
homozygous PINK1 p.Try90Leufsx12+ het PARK2 Arg275Trp in exon 7	Affected	iPS	CENSOi023-A	Female	48	Sister of #72	

Supplementary Table 4: Downregulated Genes in homozygous *PINK1* mutant DA neurons. This table lists genes that exhibit significant downregulation (log2 Fold Change (FC) > 1.1 and False Discovery Rate (FDR) < 0.05) in homozygous *PINK1* mutant DA neurons, compared to control neurons. Each gene is identified by its gene symbol, along with the log2FC value.
